# Rock sponges (lithistid Demospongiae) of the Northeast Atlantic seamounts, with description of ten new species

**DOI:** 10.7717/peerj.8703

**Published:** 2020-04-07

**Authors:** Francisca C. Carvalho, Paco Cárdenas, Pilar Ríos, Javier Cristobo, Hans Tore Rapp, Joana R. Xavier

**Affiliations:** 1 Department of Biological Sciences and K.G. Jebsen Centre for Deep-Sea Research, Bergen University, Bergen, Norway; 2 Pharmacognosy, Department of Medicinal Chemistry, Uppsala University, Uppsala, Sweden; 3 Centro Oceanográfico de Gijón, Instituto Español de Oceanografia, Gijón, Spain; 4 Departamento de Zoología y Antropología Física, Universidad de Alcalá de Henares, Madrid, Spain; 5 CIIMAR-Interdisciplinary Centre of Marine and Environmental Research, Universidade do Porto, Matosinhos, Portugal

**Keywords:** Porifera, Deep-sea, Lithistids, Biodiversity, Tetractinellida, Bubarida, New species, Biogeography

## Abstract

**Background:**

Lithistid demosponges, also known as rock sponges, are a polyphyletic group of sponges which are widely distributed. In the Northeast Atlantic (NEA), 17 species are known and the current knowledge on their distribution is mainly restricted to the Macaronesian islands. In the Mediterranean Sea, 14 species are recorded and generally found in marine caves.

**Methods:**

Lithistids were sampled in nine NEA seamounts during the scientific expeditions *Seamount 1* (1987) and *Seamount 2* (1993) organized by the MNHN of Paris. Collected specimens were identified through the analyses of external and internal morphological characters using light and scanning electron microscopy, and compared with material from various museum collections as well as literature records.

**Results:**

A total of 68 specimens were analysed and attributed to 17 species across two orders, seven families, and seven genera, representing new records of distribution. Ten of these species are new to science, viz. *Neoschrammeniella inaequalis* sp. nov., *N. piserai* sp. nov., *N. pomponiae* sp. nov., *Discodermia arbor* sp. nov., *D. kellyae* sp. nov., *Macandrewia schusterae* sp. nov., *M. minima* sp. nov., *Exsuperantia levii* sp. nov., *Leiodermatium tuba* sp. nov. and *Siphonidium elongatus* sp. nov., and are here described and illustrated. New bathymetric records were also found for *D. ramifera*, *D. verrucosa* and *M. robusta*. The Meteor seamount group has a higher species richness (15 species) compared to the Lusitanian seamount group (six species). The majority of the species had their distribution restricted to one seamount, and ten are only known from a single locality, but this can be a result of sample bias.

**Discussion:**

The number of species shared between the seamounts and the Macaronesian islands is very reduced. The same pattern repeats between the NEA and Mediterranean Sea. This study demonstrates that NEA seamounts are ecosystems with a higher diversity of lithistids than previously thought, increasing the number of lithistids known to occur in the NEA and Mediterranean Sea from 26 to 36 species.

## Introduction

The class Demospongiae Sollas (1885) contains several groups of sponges artificially unified under the name ‘lithistid demosponges’ or ‘rock sponges.’ Lithistids produce hypersilicified spicules (desmas) (Pisera & Lévi, 2002a) that usually creates a very rigid skeleton. For a very long time, they were classified into an order, Lithistida (Schmidt, 1870), but more recently, several studies have shown the polyphyletic nature of this group (Cárdenas et al., 2011; Kelly & Pomponi, 1994; Pisera & Lévi, 2002a; Schuster et al., 2015). It is now acknowledge that this trait, i.e., is the desmas, has evolved independently multiple times (Schuster et al., 2015) and the 211 valid species currently recognized worldwide are distributed in three orders-Tetractinellida Marshall (1876), Sphaerocladina Schrammen (1924) and Bubarida Morrow & Cárdenas (2015), with the large majority belonging to the former order (Morrow & Cárdenas, 2015; Pisera & Lévi, 2002a; Schuster et al., 2015; Van Soest et al., 2019, WPD).

In the Northeast Atlantic (NEA), the current state of knowledge on lithistid sponges is mainly restricted to the Macaronesian islands. So far, 17 species have been described and recorded from the Azores (Carvalho & Pisera, 2019; Gray, 1859; Topsent, 1928, 1904, 1898, 1892), Madeira and Selvagens (Bowerbank, 1869; Carter, 1873; Carvalho & Pisera, 2019; Johnson, 1863), Canary Islands (Carvalho & Pisera, 2019; Cruz, 2002; Topsent, 1892), Portugal mainland (Schmidt, 1870) and Morocco (Lendenfeld, 1907), whereas in the Mediterranean Sea, 15 species have been reported (Maldonado et al., 2015; Manconi, Serusi & Pisera, 2006; Manconi & Serusi, 2008; Perez et al., 2004; Pisera & Vacelet, 2011; Pulitzer-Finali, 1972; Vacelet, 1969). They are commonly found on hard substrate at 110–1,700 m depth (Carter, 1873; Carvalho, Pomponi & Xavier, 2015; Topsent, 1928), whereas in the Mediterranean Sea they usually occur in shallower waters or in cave systems (Manconi & Serusi, 2008; Pisera & Vacelet, 2011). Although the knowledge on distribution for lithistids in the NEA has been increasing, there is no data regarding their occurrence on seamounts in the area.

These topographic features, which provide important habitats for both benthic and pelagic organisms, are very numerous and worldwide distributed (Yesson, 2011). In the NEA, examples include the Lusitanian Seamounts (Coral Patch, Ampere, Gorringe Bank, Hirondelle II, Josephine, Lion, Dragon, Unicorn and Seine), located near the Euro-African continental shelf, approximately 250 km from the Portuguese coast and the Meteor Seamounts (Great Meteor, Hyères, Irving, Cruiser, Plato, Tyro and Atlantis), situated in the central part of the North Atlantic, close to the Mid-Atlantic Ridge (MAR) and south of the Azores archipelago. These seamounts have evoked interest for research in the late 19th and early 20th Century, and several scientific expeditions took place, such as Josephine (1869), Challenger (1873) and numerous Prince Albert I of Monaco expeditions. Late in the 20th and early 21st Centuries, new efforts aiming to explore the benthic fauna of these seamounts were undertaken. Two of these expeditions—*Seamount 1* and *Seamount 2*—organized by the Natural History Museum of Paris (MNHN), surveyed various of the Lusitanian and Meteor seamounts at depths above 1,000 m (Bouchet & Métivier, 1988; Gofas, 1993). These expeditions resulted in the discovery and description of several species of various taxonomic groups, such as brachiopods (Logan, 1998), bryozoans (Berning, Harmelin & Bader, 2017; Souto, Berning & Ostrovsky, 2016), bivalves (Dijkstra & Gofas, 2004), corals (Molodtsova & Shirshov, 2011), cirripeds (Young, 2001), hydrozoans (Ramil, Vervoort & Ansín, 1998), polychaetes (Gillet & Dauvin, 2003; Paxton & Gillet, 2004) and gastropods (Gofas, 2007) greatly advancing the understanding of the biogeographic patterns and the biodiversity of these ecosystems. However, several taxonomic groups, including sponges, remain scarcely documented in the literature for these ecosystems (Cárdenas et al., 2018; Cristobo et al., 2015; Lévi & Vacelet, 1958; Topsent, 1928; Xavier & Van Soest, 2007).

In this study, we describe the lithistid demosponges collected during the French expeditions *Seamount 1* and *Seamount 2*. New records of geographic distribution are reported, ten new species for science are described and illustrated, and the diversity and biogeographic patterns discussed. An identification key of all lithistid species reported for the NEA and Mediterranean is also provided.

## Materials and Methods

**Figure 1:**
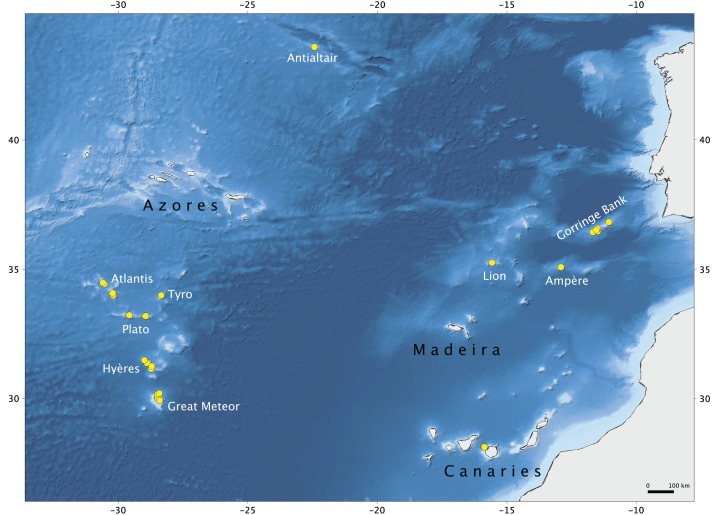
Map of the study area. Seamounts of the Northeast Atlantic and stations of the *Seamount 1* and *Seamount 2* campaigns where lithistid demosponges were collected. Map produced with the software QGIS Development Team (2019); bathymetry obtained from GEBCO Compilation Group (2019).

The material examined in this study was collected during *Seamount 1* and *Seamount 2* scientific expeditions undertaken by the MNHN of Paris to several NEA seamounts (Fig. 1; Supplemental Material S1). The main aims of these campaigns were to study the patterns of faunal diversity and endemism found on isolated seamounts in comparison to continental areas and the relation with the dispersal capacity of the various taxonomic groups. The *Seamount 1* campaign, coordinated by Dr. Philippe Bouchet, took place in 1987 onboard of the research vessel *L. Noroît*, and explored the Galicia Banks and the Lusitanian Seamounts (Gorringe, Josephine, Ampère, Lion and Seine) (Bouchet & Métivier, 1988). The second campaign, *Seamount 2*, this time lead by Dr. Serge Gofas, explored the Meteor Seamounts group (Great Meteor, Hyères, Irving, Cruiser, Plato, Atlantis and Tyro) and the Antialtair Seamount on board of the RV *L. Suroît*, sampling 165 stations also at depths above 1,000 m (Gofas, 1993). Lithistids were collected in 10 stations on *Seamount 1* (11%) and in 42 stations on *Seamount 2* (32%) between 280 and 1,035 m depth using various sampling gears (beam trawl (CP), epibenthic dredge (DE) and Warén dredge (DW)), and preserved in formalin onboard. The specimens examined are deposited in the ‘zoothèque’ of the MNHN in Paris, and stored at room temperature in ethanol 70%. Detailed information regarding the collection of the specimens studied here, is deposited in PANGAEA^®^ Data Publisher (www.pangaea.de) under the digital object identifier (DOI): https://doi.pangaea.de/10.1594/PANGAEA.896492.

The specimens were analysed through the use of Light Microscopy (LM) and Scanning Electron Microscopy (SEM). For light microscopy, cross sections and slides of loose spicules were mounted in Canada Balsam^®^ Sigma–Aldrich or Eukit^®^ Sigma–Aldrich following standards procedures (Boury-Esnault & Rutzler, 1997). In addition, a few specimens, representative of each species, were selected and prepared for SEM. For this purpose, pieces of both the ectosome and choanosome of the sponge were excised and then either directly mounted or digested in nitric acid, washed several times with distilled water and then fixed in ethanol. The spicules were then placed on a stub and covered with gold-paladium. Thirty spicules of each spicule type were measured using the Leica Application Suite (LAS v. 4.5), for individual specimens. Minimum, mean and maximum values are presented for the measurements obtained for each analysed specimen. For the higher taxa classification, we followed the revised Demospongiae classification (Morrow & Cárdenas, 2015).

Due to the formalin fixation, we were not able to extract DNA for molecular analysis, and any attempts to barcode the mitochondrial COI gene, including the mini-barcode protocol used in other tetractinellids (Cárdenas & Moore, 2017) were unsuccessful.

The electronic version of this article in PorTable Document Format (PDF) will represent a published work according to the International Commission on Zoological Nomenclature (ICZN), and hence the new names contained in the electronic version are effectively published under that Code from the electronic edition alone. This published work and the nomenclatural acts it contains have been registered in ZooBank, the online registration system for the ICZN. The ZooBank Life Science Identifiers (LSIDs) can be resolved and the associated information viewed through any standard web browser by appending the LSID to the prefix http://zoobank.org/. The LSID for this publication is: urn:lsid:zoobank.org:pub:A0DA0236-4579-47A4-8BE4-E68803C2EC8F. The online version of this work is archived and available from the following digital repositories: PeerJ, PubMed Central and CLOCKSS.

## Results

**Figure 2:**
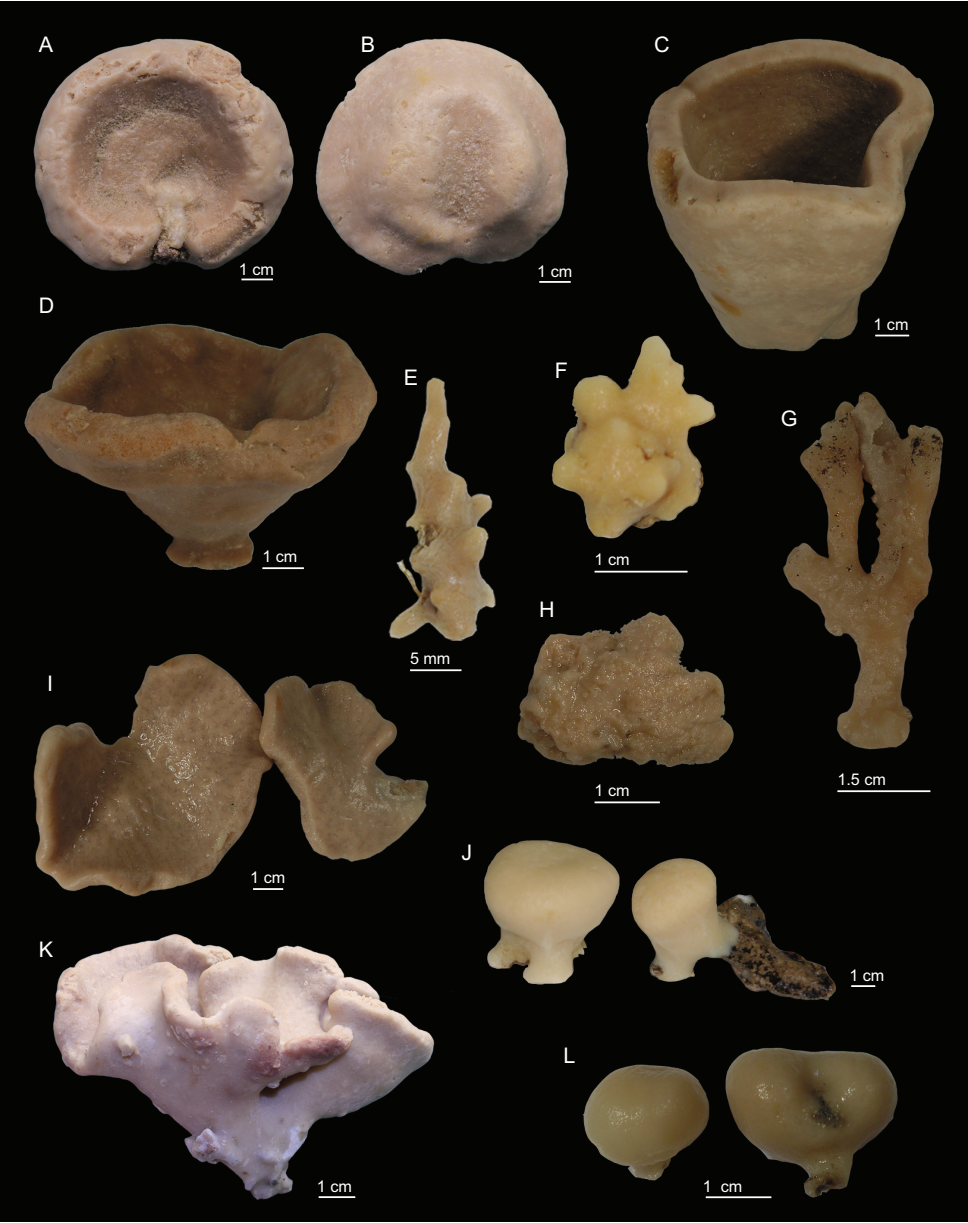
Specimens collected during *Seamount 1* and *Seamount 2* expeditions. (A) Top view of *Neoschrammeniella inaequalis* sp. nov., holotype MNHN-IP-2018-84, (B) bottom view of *N. inaequalis* sp. nov., holotype MNHN-IP-2018-84. (C) *N. piserai* sp. nov., holotype MNHN-IP-2008-234. (D) *N. pomponiae* sp. nov., holotype MNHN-IP-2008-233. (E) *Discodermia ramifera* Topsent, 1892, specimen MNHN-IP-2008-213. (F) *D. verrucosa* Topsent, 1928, specimen MNHN-IP-2008-205. (G) *D. arbor* sp. nov., holotype MNHN-IP-2008-211. (H) *D. kellyae* sp. nov., holotype MNHN-IP-2008-208. (I) *Macandrewia* cf. *azorica*, specimen MNHN-IP-2008-220. (J) *M. robusta* Topsent, 1904, specimens MNHN-IP-2008-216. (K) *M. schusterae* sp. nov., holotype MNHN-IP-2018-87. (L) *M. minima* sp. nov., holotype MNHN-IP-2008-222.

**Figure 3:**
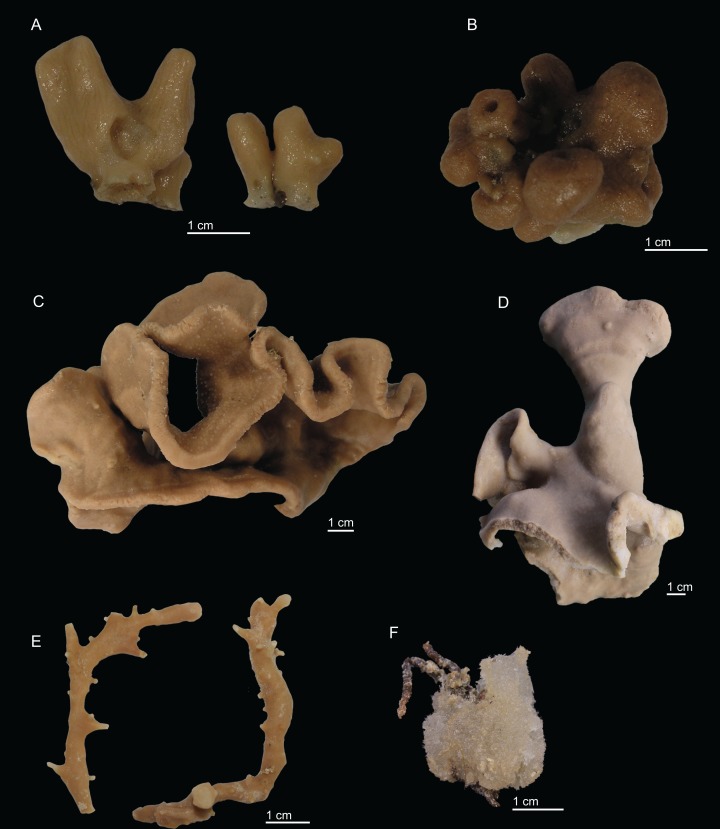
Specimens collected during *Seamount 1* and *Seamount 2* expeditions. (A) *Exsuperantia archipelagus* Carvalho & Pisera (2019), specimen MNHN-IP-2008-196. (B) *E. levii* sp. nov., holotype MNHN-IP-2008-201. (C) *Leiodermatium lynceus* Schmidt (1870), specimen MNHN-IP-2008-239. (D) *L. tuba* sp. nov., holotype MNHN-IP-2018-72. (E) *Siphonidium elongatus* sp. nov., holotype MNHN-IP-2008-236. (F) *Petromica (Petromica) grimaldii* Topsent, 1898, MNHN-IP-2018-92.

In this study we analysed 68 specimens, collected between 280 and 1,035 m depth on eight NEA seamounts, and assigned them to 17 species distributed across two orders, seven families, and seven genera (Figs. 2–3). Of these, ten species are new for science—*Neoschrammeniella inaequalis* **sp. nov**., *N. piserai* **sp. nov***., N. pomponiae* **sp. nov.,** *Discodermia arbor* **sp. nov.,** *D. kellyae* **sp. nov.,** *Macandrewia schusterae* **sp. nov.,** *M. minima* **sp. nov.,** *Exsuperantia levii* **sp. nov.,** *Leiodermatium tuba* **sp. nov.** and *Siphonidium elongatus* **sp. nov** (see below descriptions and illustrations). All analysed material is described and illustrated below and compared with additional specimens from various museum collections (MNHN, HBOI, RMNH and DOP). An identification key for all lithistid species recorded to date for the NEA and MED is also provided. All new species described here have the taxonomic authority restricted to the first and last author.

## Systematic Index

Phylum Porifera Grant, 1836

Class Demospongiae Sollas, 1885

Subclass Heteroscleromorpha Cárdenas, Pérez & Boury-Esnault, 2012

Order Tetractinellida Marshall, 1876

Suborder Astrophorina Sollas, 1887

Family Corallistidae Sollas, 1888

**Genus *Neoschrammeniella*** Pisera & Lévi, 2002b

Species *Neoschrammeniella inaequalis* **sp. nov.**

Species *Neoschrammeniella piserai* **sp. nov.**

Species *Neoschrammeniella pomponiae* **sp. nov.**

Family Theonellidae Lendenfeld, 1903

**Genus *Discodermia*** du Bocage, 1869

Species *Discodermia ramifera* Topsent, 1892

Species *Discodermia* cf. *ramifera* Topsent, 1892

Species *Discodermia verrucosa* Topsent, 1928

Species *Discodermia arbor* **sp. nov.**

Species *Discodermia kellyae* **sp. nov.**

Family Macandrewiidae Schrammen, 1924

**Genus *Macandrewia*** Gray, 1859

Species *Macandrewia* cf. *azorica* Gray, 1859

Species *Macandrewia robusta* Topsent, 1904

Species *Macandrewia schusterae* **sp. nov.**

Species *Macandrewia minima* **sp. nov.**

Family Phymaraphiniidae Schrammen, 1924

**Genus *Exsuperantia*** Özdikmen, 2009

Species *Exsuperantia archipelagus* Carvalho & Pisera, 2019

Species *Exsuperantia levii* **sp. nov.**

Suborder Spirophorina Bergquist & Hogg, 1969

Family Azoricidae Sollas, 1888

**Genus *Leiodermatium*** Schmidt, 1870

Species *Leiodermatium lynceus* Schmidt, 1870

Species *Leiodermatium tuba* **sp. nov.**

Family Siphonidiidae Lendenfeld, 1903

**Genus *Siphonidium*** Schmidt, 1879

Species *Siphonidium elongatus* **sp. nov.**

Order Bubarida Morrow & Cárdenas, 2015

Family Desmanthidae Topsent, 1893

**Genus *Petromica*** Topsent, 1898

Subgenus *Petromica* (*Petromica*) Topsent, 1898

Species *Petromica* (*Petromica*) *grimaldii* Topsent, 1898

**Species descriptions**

**Order TETRACTINELLIDA Marshall, 1876**

**Suborder ASTROPHORINA Sollas, 1887**

**Family CORALLISTIDAE Sollas, 1888**

**Genus *Neoschrammeniella*Pisera & Lévi, 2002b**

**Synonymy.** *Iouea* sensu Lévi & Lévi, 1988: 248.

**Diagnosis.** Corallistidae with smooth dichotriaenes and two to three types of microscleres: metasters, amphiasters/streptasters and/or spirasters (emended after Kelly, 2007; Pisera & Lévi, 2002b; Pisera & Vacelet, 2011; Schlacher-Hoenlinger, Pisera & Hooper, 2005).

**Definition.** Polymorphic Corallistidae, shallow cup-shaped or deep vase-shaped; surface can be smooth or rugose; ectosomal megascleres are smooth dichotriaenes; choanosomal megascleres are dicranoclone desmas with different types of ornamentation, varying from poorly to extremely tuberculated in different species; diactines are frequently present in the ectosome and triaenes are rare; microscleres are metasters, amphiaster/streptaster and/or acanthose spirasters (type I covered by short blunt rays, and type II irregular with short blunt rays only on the edges), but the number and type of microscleres varies between species (emended after Kelly, 2007; Pisera & Lévi, 2002b; Pisera & Vacelet, 2011; Schlacher-Hoenlinger, Pisera & Hooper, 2005).

**Type species.** *Neoschrammeniella moreti* Lévi & Lévi, 1988 (type by monotypy).

***Neoschrammeniella inaequalis* sp. nov.**

**Figure 4:**
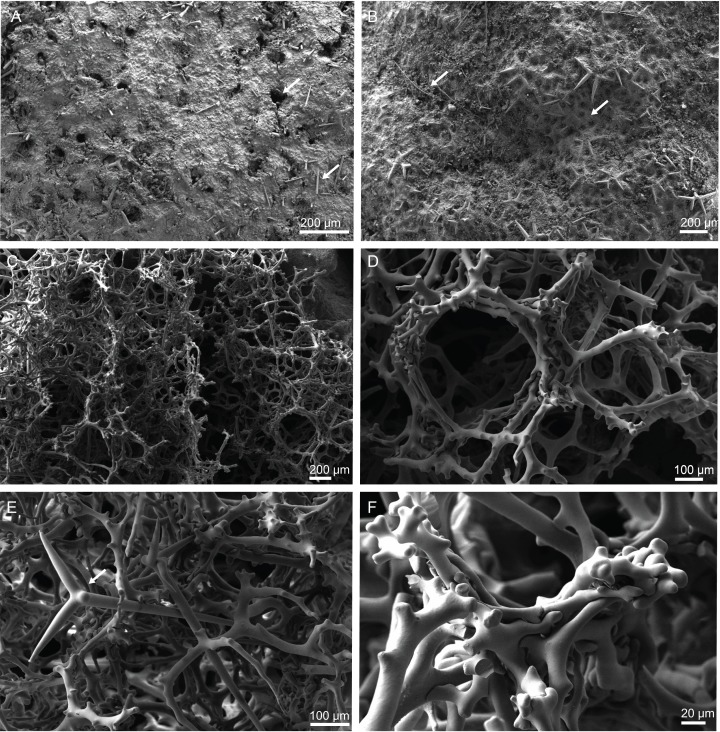
Surface and skeleton of *Neoschrammeniella inaequalis* sp. nov., holotype MNHN-IP-2018-84. (A) Upper surface, showing the openings and some oxeas, (B) lower surface, showing oxeas and small openings, (C) overview of choanosomal desmas, (D) dicranoclones desmas, (E) plagiotriaenes crossing the desmas, (F) detail of the ornamentation of the desmas and zygosis.

**Figure 5:**
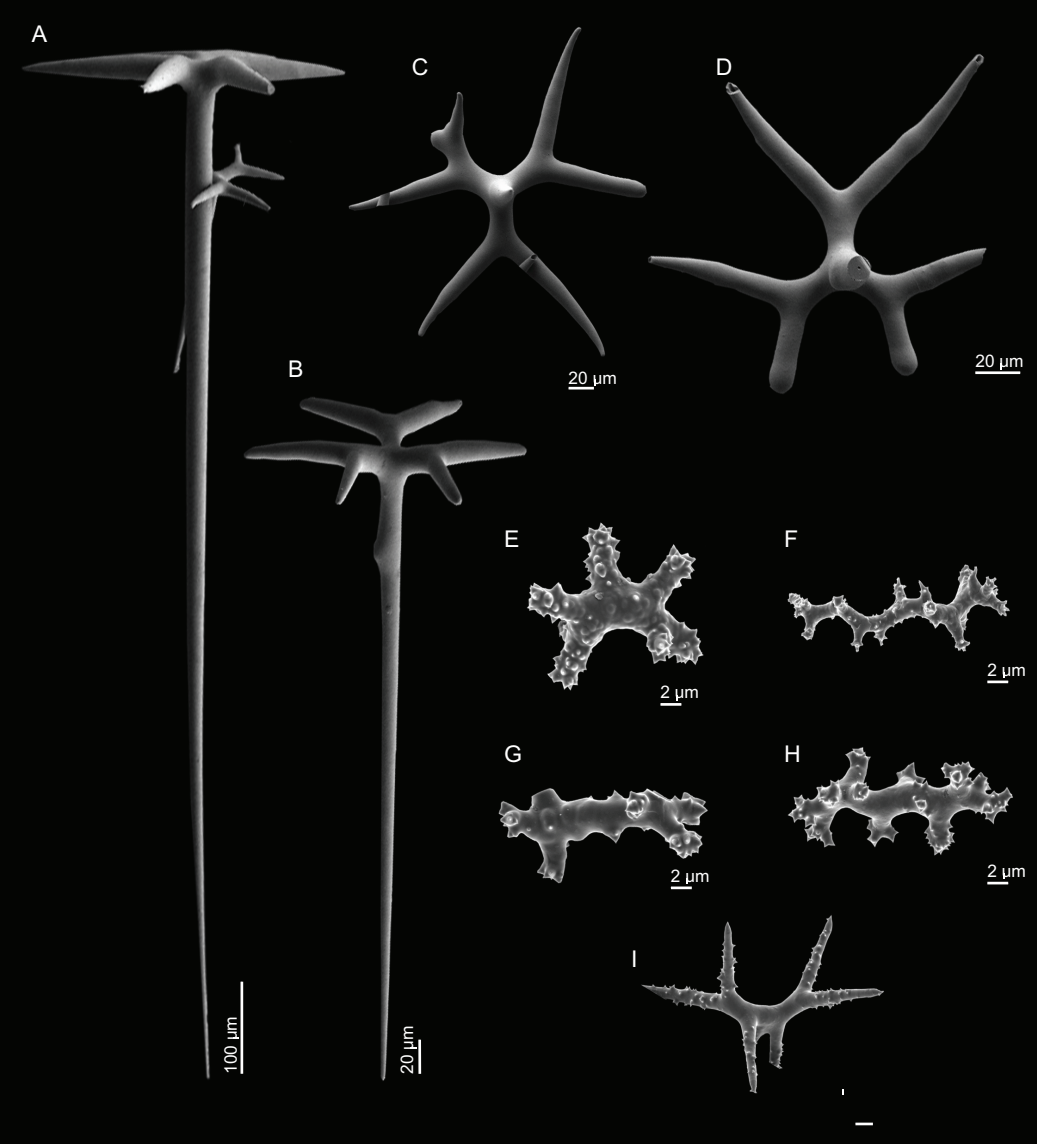
Spicules of *Neoschrammeniella inaequalis* sp. nov., holotype MNHN-IP-2018-84. (A) Two dichotriaenes with different size classes, (B) small dichotriaene with a protuberance in the rhabdome, (C) and (D) irregular cladomes, (E)–(H) variation of spirasters, (I) metaster.

Figures 2A–2B, 4–5 and Table 1

**Table 1:** Comparative table of external morphology and spicular micrometries of all *Neoschrammeniella* species recorded in the North Atlantic Ocean. Spicule measurements (*n* = 30 unless stated otherwise) are presented as minimum–mean–maximum. Data compiled from the original descriptions, or subsequent re-descriptions of type material (marked with numbers).

	Habitus	Size	Dicranoclones	Dichotriaenes	Oxeas	Spirasters	Metasters	Locality
^1^*N. bowerbankii* Holotype BMNH 69.11.60.1 (PZS 1862)	–	–	–	Cladome: 319–397 µm in diameter; rhabdome 487–939 µm length	–	20.2–23.7 × 7.0–11.7 µm (as spiraster type I)	28.3–39.3 × 19.6–32.7 µm (as spiraster type II)	Madeira (depth unknown)
^2^*N.bowerbankii* (Johnson, 1863)	Cup-shaped to contornated lamellate masses with thick walls; colour white	80 × 60 × 60 mm in size	290–402 µm in size	Cladome: 176–323 µm; rhabdome: 223–513 µm	340–820 × 1.5–2.5 µm	Short arms, 17–24 × 7.06–11.1 µm in size (as spiraster type I)	Long arms, 26.2–39.2 × 18.5–23.9 µm (as spiraster type II)	Mediterranean Sea (20–22 m)
*N. inaequalis* sp. nov. (Holotype MNHN-2018-84)	Flattened cup-shape, with a concave center; both surfaces are smooth; colour light brown	73 × 64 × 29 mm in size; walls, 14–17 mm thick	354–576–975 × 12–25–39 µm (*n* = 12)	Cladome: smooth, very irregular, 118–233–406 µm; rhabdome: long with a round tip, 136–432–1211 × 9–18–31 µm	Large, thin, curved, 670–1144 × 5.2–7.8–13.4 µm (*n* = 5)	Short with thick arms, very abundant, 12.1–18.5–26.6 µm	Long and thin arms, 14.6–31.6–47.9 µm	Gorringe Seamount (605–675 m depth)
*N. inaequalis* sp. nov. (Paratype MNHN-2018-85)	Small, ball shaped with a concave top; both surfaces are smooth; colour light brown	34 mm diameter, 20 mm height	308–431–575 × 21–34–49 µm (*n* = 15)	Cladome: 158–298–463 µm; rhabdome: 221–550–1228 × 13–23–38 µm	Large, thin, curved 449–1034 × 5–7–10 µm (*n* = 8)	10.4–20.3–26.1 µm	15.1–32.7–47.6 µm (*n* = 17)	Gorringe Seamount (605–675 m depth)
*N. piserai* sp. nov. (Holotype MNHN-IP-2008-234)	Large cup-rectangular sponge attached to the substrate by the entire lower base; both surfaces smooth; colour beige	69 mm in diameter at the top, and 43 mm at the base, 98 mm height; walls, 11 mm thick	280–428–522 × 16–25–37 µm (*n* = 6)	Cladome: smooth, 153–244–389 µm; rhabdome: long with a round tip, 198–366–535 × 10–19–33 µm	Not present	Short with thick arms, very abundant, 14.7–18.7–23.7 µm; some very irregular, rhab-like, 13.5–17.8–23.1 µm	Long and thin arms, 18.9–30.7–41.5 µm	Plato Seamount (695 m depth)
*N*. *pomponiae* sp. nov. (Holotype MNHN-IP-2008-233)	Cup-rounded shape	54 × 81 mm in size with a small pedicel, 23 mm in size; walls, 11 mm thick	185–427–666 × 18–39–88 µm (*n* = 13)	Cladome: 157–274–374 µm; rhabdome: 239–478–684 × 11–21–37 µm (*n* = 17)	Large, thick, 1,455–1,643 × 17–18 µm (*n* = 2)	Very abundant, 10.7–18.9–35.8 µm	16.2–27.6–39.3 µm	Hyères Seamount (480 m depth)

**Notes:**

1 Information provided by Prof. A. Pisera, 2019, personal communication.

2 Re-description in Pisera & Vacelet (2011).

‘–’ no information/not mentioned.

Urn:lsid:zoobank.org:act:8A516D9B-5351-47AF-8EC2-7EBC44166D35

**Holotype.** MNHN-IP-2018-84 (1988-09-26, Gorringe Seamount, beam trawl, CP28, 36°38′N, 11°29.8′W, 605–675 m, *Seamount 1* campaign).

**Paratype.** MNHN-IP-2018-85 (1988-09-26, Gorringe Seamount, beam trawl, CP28, 36°38′N, 11°29.8′W, 605–675 m, *Seamount 1* campaign).

**Other material.** MNHN IP-2018-86 (1988-09-24, Gorringe Seamount, beam trawl, DW21, 36°34.9′N, 11°28.4′W, 460–480 m, *Seamount 1* campaign).

**Comparative material examined.** *Neoschrammeniella bowerbankii* (Johnson, 1863) (HBOM 003:00592, Madeira), *N. bowerbankii* (HBOM 003:00810, Madeira), *N. piserai* sp. nov. (MNHN-IP-2008-234, Plato Seamount), *N. pomponiae* sp. nov. (MNHN-IP-2008-233, Hyères Seamount).

**Diagnosis.** Cup-shaped *Neoschrammeniella* with rounded edges and smooth surfaces; dicranoclone desmas of vine-like appearance; irregular dichotriaenes.

**Description (holotype MNHN-IP-2018-84).** Massive, flattened cup-shaped, with a concave centre, 73 mm length, 29 mm high and 64 mm wide (Fig. 2A); top surface is smooth with some oxeas perforating the surface and several small openings evenly distributed; walls are rounded and thick, 14–17 mm wide; bottom surface is also smooth, full of little openings dispersed throughout the entire surface, 31–56 μm in diameter, and some oxeas (Fig. 2B); colour is light brown in ethanol; the smooth surfaces could indicate that these specimens were not attached to any substrate, and therefore had a free living mode (Fig. 2B).

**Skeleton.** Ectosomal skeleton composed of smooth dichotriaenes of variable shape and size, along with a dense layer of microscleres (Figs. 4A and 4B); long-shafted triaenes or under-developed dichotriaenes, can also be observed (Fig. 4E); choanosomal skeleton is made of an irregular and loose network of dicranoclone desmas (Figs. 4C and 4D), spirasters and metasters; oxeas can be observed crossing the skeleton and projecting the surface.

**Spicules (holotype MNHN-IP-2018-84).**

1. **Dicranoclones**, smooth, irregular, slender, of vine-like appearance, 354–576–975 × 12–25–39 μm in size; clones can have few to several tubercles, that are smooth or slightly rugose (Figs. 4C–4F);
2. **Oxeas**, large, thin, curved, 670–1,144 × 5.2–7.8–13.4 μm in size (Figs. 4A and 4B);
3. **Dichotriaenes**, have a smooth cladome, that can be very irregular, having rounded or pointed tips, or clades of unequal size, 118–233–406 μm in diameter (Figs. 5A–5D); rhabdome is either short or long, and has a rounded tip, 136–432–1,211 × 9–18–31 μm in size (Fig. 5A); small branches or protuberances can be observed on the rhabdome, but they are uncommon (Fig. 5B);
4. **Spirasters**, with short and thick arms, mainly spiny on the arms, 12.1–18.5–26.6 μm in size (Figs. 5E–5H);
5. **Metasters**, less abundant, covered by spines, with long and thin arms, 14.6–31.6–47.9 μm in size (Fig. 5I).

**Distribution.** *N. inaequalis* sp. nov. was found in the Gorringe Seamount between 460 and 675 m depth.

**Etymology.** From the latin *inaequalis* = unequal, due to the uneven and irregular cladomes of the dichotriaenes.

**Remarks.** *N. inaequalis* sp. nov. is a distinct species due to (1) the growth form, being flattened cup-shaped with a concave center; (2) the fact that both surfaces were completely smooth may indicate that the sponge is free-living, i.e., not attached to the substrate; (3) triaenes can be present, although rare, being the second time this kind of spicule is reported for the genus (see illustration of the redescription of *N. moreti* (Lévi & Lévi, 1988)) in *Systema Porifera* (Pisera & Lévi, 2002b); (4) the vine-like desmas also resemble the desmas found in the genus *Isabella* (Carvalho, Pomponi & Xavier, 2015; Ekins et al., 2016; Schlacher-Hoenlinger, Pisera & Hooper, 2005); (5) the shape and ornamentation of desmas are distinct from the other *Neoschrammeniella* species (see descriptions below and Remarks under *N. pomponiae* sp. nov.). It is also important to note that this species presents dichotriaenes very variable in size and shape (cladomes are irregular and unequal, and rhabdomes can present small protuberances or branches), so far only found in *Isabella* spp. (Carvalho, Pomponi & Xavier, 2015; Schlacher-Hoenlinger, Pisera & Hooper, 2005). These irregularities can be attributed to a pathologic development.

***Neoschrammeniella piserai* sp. nov.**

**Figure 6:**
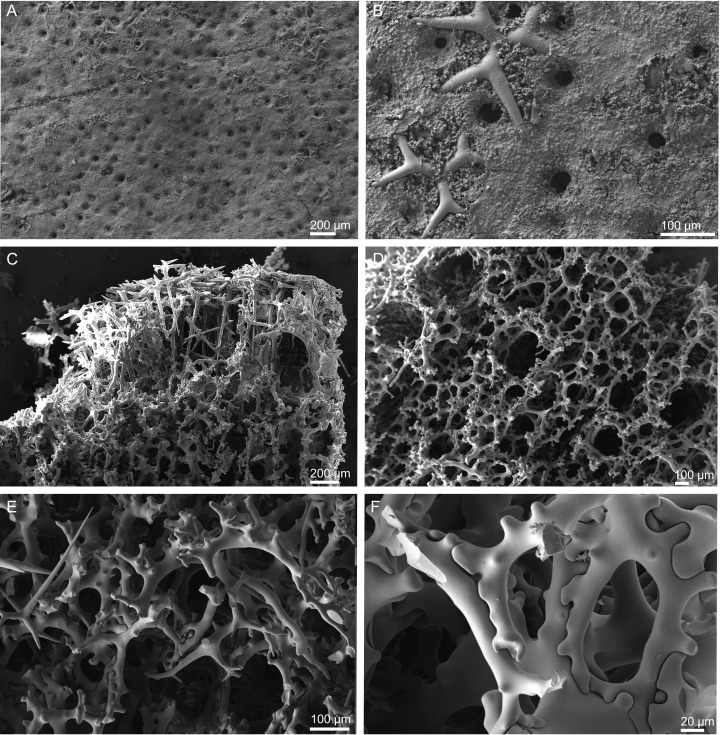
Surface and skeleton of *Neoschrammeniella piserai* sp. nov., holotype MNHN-IP-2008-234. (A) Overview of the surface with several openings, (B) close up of the surface where dichotriaenes are surrounded by a large number of microscleres, (C) overview of the skeleton showing the separation of the ectosome, made by a layer of dichotriaenes, and the choanosome composed of desmas, (D) dicranoclone desmas, (E) detail of dicranclone desmas, (F) zygosis and detail on the sculpture of the desmas.

**Figure 7:**
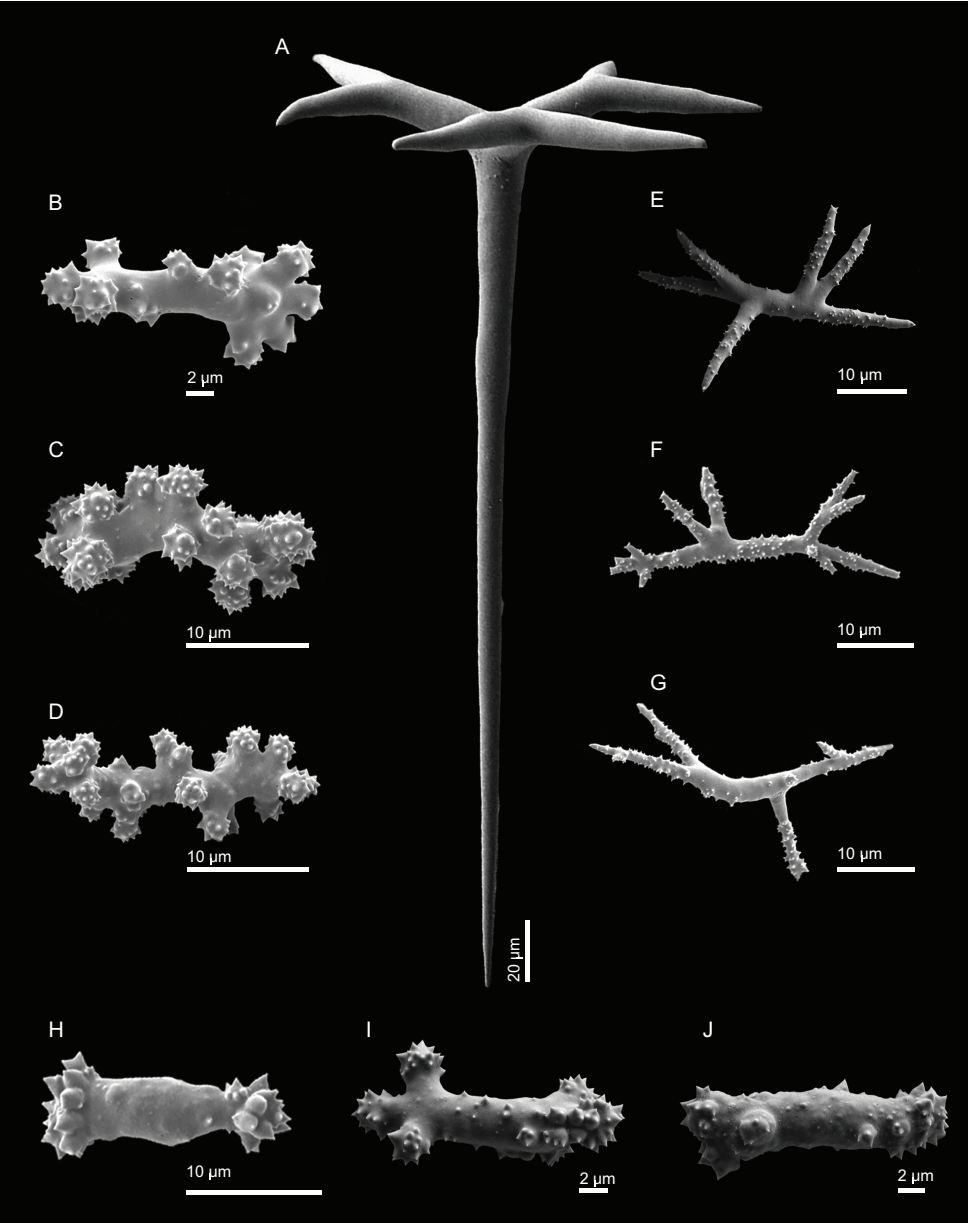
Spicules of *Neoschrammeniella piserai* sp. nov., holotype MNHN-IP-2008-234. (A) Dichotriaene, (B)–(D) spirasters, (E)–(G) mestasters, (H)–(J) underdeveloped spirasters.

Figures 2C, 6–7 and Table 1

Urn:lsid:zoobank.org:act:77F1F52E-28C9-43C0-A501-1ADAD03241A5

**Holotype.** MNHN-IP-2008-234 (1993-01-31, Plato Seamount, epibenthic Warén dredge, DW241, 33°12′N, 28°59′W, 695 m, *Seamount 2* campaign).

**Comparative material examined.** *N. bowerbankii* (HBOM 003:00592, Madeira), *N. bowerbankii* (HBOM 003:00810, Madeira), *N. inaequalis* sp. nov. (holotype MNHN-IP-2018-84 and paratype MNHN-IP-2018-85, Gorringe Seamount), *N. pomponiae* sp. nov. (holotype MNHN-IP-2008-233, Hyères Seamount).

**Diagnosis.** Cup rectangular shaped *Neoschrammeniella* fixed to the substratum by the entire base; oxeas not present.

**Description (holotype MNHN-IP-2008-234).** Large cup-rectangular sponge, 98 mm height and 69 mm width on top; the sponge was attached to the substratum by the entire base, which has 43 mm in diameter; walls are 11 mm thick (Fig. 2C); surfaces are smooth with visible subdermal water canals and openings evenly distributed on both surfaces, 20–44 μm in diameter (Figs. 6A and 6B), colour beige in ethanol.

**Skeleton.** Ectosomal skeleton is made of a layer of dichotriaenes perpendicular to the surface, and a dense layer of numerous microscleres (Fig. 6C); choanosomal skeleton has a net of compact dicranoclone desmas with several metasters and spirasters spread out through the tissue.

**Spicules (holotype MNHN-IP-2008-234).**

1. **Dicranoclones**, irregular, usually smooth, 280–428–522 × 16–25–37 μm in size; the rays of the desmas have several ramifications and some tubercles, that are usually smooth (some can have a rugosity) (Figs. 6C–6F).
2. **Dichotriaenes**, with a smooth cladome, 153–244–389 μm in diameter; rhabdome has a rounded tip and 198–366–535 × 10–19–33 μm in size (Fig. 7A).
3. **Metasters**, covered by spines, with long and thin arms, 18.9–30.7–41.5 μm in size (Figs. 7E–7G).
4. **Spirasters**, spiny, with short and thick arms, very abundant, 14.7–18.7–23.7 μm in size (Figs. 7B–7D); some can present an irregular shape, i.e., rhabd-like with spiny tips, scarce, 13.5–17.8–23.1 μm in size (Figs. 7H–7J) (see “Remarks”).

**Distribution.** *N. piserai* sp. nov. is only known from its type locality, Plato Seamount (695 m depth).

**Etymology**. Named after Professor Andrzej Pisera from the Institute of Paleobiology Warszawa (ZPAL), in recognition of his outstanding contributions on the taxonomy of both fossil and extant lithistid sponges.

**Remarks.** The peculiar external morphology (cup-rectangular shape) of *N. piserai* sp. nov., together with the smooth surface, the ornamentation of the desmas are the features that differentiate this new species from the other NEA and MED *Neoschrammeniella* species (Table 2; Remarks under *N. pomponiae* sp. nov.). One could also not observe oxeas on this species, a spicule type that was found in other *Neoschrammeniella* spp. from the NEA and MED. Some spirasters presented an irregular shape. They were rhabd-like with spiny tips (Figs. 7H–7J) and they had approximately the same size as the typical spirasters. Since these underdeveloped spirasters were scarce we decided to include them in the same category of spirasters, but analyses of new material may show that they belong to a different category.

**Table 2:** Comparative table of external morphology and spicular micrometries of all *Discodermia* species recorded in the North Atlantic Ocean and Mediterranean Sea. Spicule measurements (*n* = 30 unless stated otherwise) are presented as minimum–mean–maximum. Data compiled from the original descriptions, or subsequent re-descriptions of type material (marked with numbers).

	Habitus	Size	Tetraclones	Discotriaenes	Oxeas	Acanthomicroxeas	Acanthorhabds	Locality
^1^*D. polydiscus* (Bowerbank, 1869) (Holotype BMNH 40.10.23.12)	Small irregular mushroom shaped, with strongly concave upper side; short stem and slightly expanded attachment base	25 × 20 mm large, 18 mm high	Regular massive with strongly branched and tuberculated zygomes and smooth rays; 300–450 µm in size and 100–110 µm thick	Cladome: round to oval, 250–350 µm in diameter; rhabdome: short and conical, 87–108 µm	Present	Slender, fusiform and slightly curved or bent acanthoxeas (spines are hook-like), 38–59 µm long, 2.4–4 µm thick	Fusiform, massive, 15– 22 µm long, 2–4.5 µm thick	St. Vincent Island, Caribbean (depth unknown)
*D. inscripta* (Schmidt, 1879**)** (unknown type)	^2^*Incertae sedis* (type material is deciduous: ectosomal discotriaenes and microscleres were not found)							
^3^*D. dissoluta* Schmidt, 1880 (HBOM 003:01093)	Cluster of knobby fingers; colour is purple brown in exterior and cream-coloured in interior when alive	200 mm diameter, 50 mm tall and 10 mm in diameter	Smooth, regular, with a weak zygosis, 475–525 µm in size	Cladome: round, concave, smooth (except growth lines), 203–294 µm in diameter; rhabdome: short, delicate and conical	Curved oxeas/styles, 500–530 × 9–10 µm	Fusiform, 41.6–68.0 × 5.5–6.1 µm in size	Fusiform with pointed tips, 15.1–18.9 × 4.3–5.2 µm in size	Florida (81 m depth)
^4^*D. ramifera* Topsent, 1892 (Holotype)	Sponge more and less elongated with several finger-like extensions; water canals visible under the surface; smooth surface; colour is white in ethanol	1–15 mm wide; finger-like extensions 2–20 × 2–3 mm	Desmas rays full of tubercles in the extremities	Whole or barely lobed, 300 µm diameter	Present	Numerous, fusiform, spiny, curved, seldom centrotylotes, 40–45 µm long	Very abundant, thorny, often curved, 20–25 µm long	Azores (318 m depth)
*D. ramifera* (specimen MNHN-IP-2008-213)	Small, elongated to branching shape sponge; colour is beige to light yellow	15–29 mm high and 3–10 mm thick	182–328–470 × 24–32–48 µm in size (*n* = 19)	Cladome: very variable in shape, 124–160–213 µm in diameter (*n* = 16); rhabdome: 23–32–40 × 8–10–14 µm (*n* = 9)	Present (all broken)	Slightly curved, thorny, 22.8–27.6–32.6 × 1.0–1.5–1.8 µm (*n* = 15)	Thorny with blunt tips, 3.9–10.3–13.9 × 1.1–1.4–1.9 µm (*n* = 19)	Great Meteor Seamount (320 m depth)
*D*. cf. *ramifera* (specimen MNHN-IP-2008-210)	Small, elongated; colour is beige	20 high and 10 mm thick (fragment)	400–455–534 × 30–51–82 (*n* = 20)	Cladome: 195–328–560 µm; rhabdome: 20–42–68 × 9.5–20.3–37.9 µm (*n* = 16)	Present	24.6–39.0–59.8 × 1.8–3.3–5.4 µm	15.2–20.2–24.2 × 2.1–2.9–4.4 µm	Atlantis Seamount (420 m depth)
^5^*D. verrucosa* Topsent, 1928 (Holotype MNHN DT 1199)	Cup-shaped with rounded edges and numerous warts; irregular contour and a depressed center; short pedicel laterally compressed; colour is grey-yellow in ethanol	35–38 mm high and 58 mm wide	Skeleton is very solid and regular, desmas are robust and have a complex zygosis; Protoclad with tubercles and 60 µm; deuteroclad has several cylindrical nodules intended for zygosis	Cladome: flat, variable shapes, 360–400 µm on average (can vary between 200 and 560 µm); Rhabdome: conical shape, simple, 100 µm long	Slightly curved, bigger than 1 mm, rarely exceeding 7 µm width	Numerous, fusiform, spiny, slightly sharp, 43–52 × 3–3.5 µm	More abundant than microxeas, 15–17 × 2–2.8 µm	Gran Canaria (400 m depth)
*D. verrucosa* (specimen MNHN-IP-2008-205)	Spherical polymorphic with several rounded protuberances; colour varies from whitish to light brown	15–20 high and 12–13 mm wide	106–170–278 (*n* = 19) × 19–34–46 µm in size	Cladome: 102–153–222 µm in diameter (*n* = 17); rhabdome: 15–25–47 × 5–8–13 µm (*n* = 9)	Broken	22.8–35.2–53.5 × 1.3–2.2–3.9 µm	7.5–12.9–19.0 × 1.2–1.6–3.0 µm	Atlantis Seamount (338 m depth)
^6^*D. polymorpha* Pisera & Vacelet, 2011 (Holotype ZPAL Pf.21/1)	Small and polymorphic, nearly spherical to irregular masses with protuberances; can be attached to the by a short pedicel or the entire surface	Up to 57 mm in diameter	Irregular skeleton; desmas are smooth with poorly branches tips, 370–718 µm in diameter	Cladome: very variable in shape, 174–366 µm in diameter; rhabdome: 60–65 µm long	Not present	Spinous, very variable, 24.8–68.3 × 1.66–3.78 µm	Very variable, cylindrical to fusiform, 13.20–37.20 × 1.85–4.25 µm	3PPs Cave, Marseille area, France (3–20 m depth)
^7^*D. adhaerens* Van Soest, Meesters & Becking, 2014 (Holotype RMNH Por. 9241)	Thinly to massively encrusting limestone rockwalls with a smooth surface; colour is bright orange	Several dm^2^ in lateral expansion, 2–3 mm thick	Large, robust, with arms heavily tuberculated, 320–428–520 µm long and 40–66 µm thick	Discs: 130–202–350 × 100–155–280 µm; Rhabds 24–34–41 µm	Thin, curved, with wispy endings, 670–795–910 × 5–6.3–7 µm	Not present	15–20–25 µm (as acanthomicrorhabds)	Bonaire (146 m depth)
*D. arbor* sp. nov. (Holotype MNHN-IP-2008-211)	Massive discodermia of tree like appearance, with a long stem and three branches; surface is smooth; colour is beige in ethanol	Full sponge length is 58 mm; stem is 15 mm high and 7.5–12 mm wide and branches are 13–28 mm long	Usually with the arms tuberculated, but can be smooth; very strong zygoses;181–392–567 × 15–36–56 µm in size	Cladome: 148–256–396 µm in diameter; rhabdome: 34–53–71 × 15–21–24 µm (*n* = 9)	Not present	Slightly curved, spinous, with sharp tips, 24.1–35.1–50.1 × 1.4–2.3–3.5 µm	Covered by numerous spines, with unequal tips (blunt or sharp) 6.7–16.1–25.9 × 1.1–2.2–4.3 µm	Great Meteor Seamount (330 m depth)
*D. kellyae* sp. nov. (Holotype MNHN-IP-2008-208)	Massive sponge, polymorphic of bulb appearance, with large protuberances of round shape; surface is irregular with a crumble/rugose appearance; colour is beige to light brown	53 mm high and 31 mm wide	Large, compact, thick, 112–338–589 × 20–42–76 µm (*n* = 20)	Cladome: very variable in shape and size, 121–289–425 µm in diameter; rhabdome 36–81–142 × 13–31–44 µm	Strongyles, one tip rounded and the other one sharp, 418–444 × 6.0–7.9 µm in size (*n* = 2)	Straight or curved, with sharp tips, spinous, 16.7–43.2–66.5 × 1.5–2.5–3.7 µm	Spinous, with blunt tips, 5.3–13.3–24.9 × 1.2–2.1–3.7 µm	Plato Seamount (580 m depth)

**Notes:**

1 Redescription in Pisera & Lévi (2002c).

2 Pisera & Lévi (2002d).

3 This description was taken from Pisera & Pomponi (2015) since the species was poorly described in the original and no information on the spicules measurements was given.

4 Topsent (1892).

5 Topsent (1928).

6 Pisera & Vacelet (2011).

7 Van Soest, Meesters & Becking (2014).

***Neoschrammeniella pomponiae* sp. nov.**

**Figure 8:**
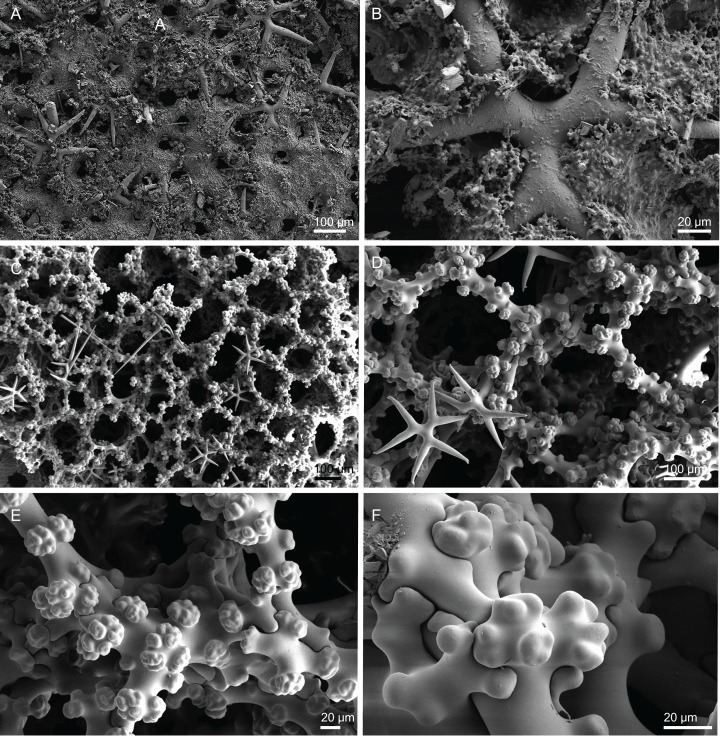
Surface and skeleton of *Neoschrammeniella pomponiae* sp. nov., holotype MNHN-IP-2008-233. (A) Surface showing several openings, dichotriaenes and some oxeas protruding the surface, (B) detail of the surface with a dichotriaene surrounded by numerous microscleres, (C) overview of the dicranoclone desmas, (D) choanosomal dicranoclone desmas, (E) detail of the sculpture of the desmas, (F) zygosis.

**Figure 9:**
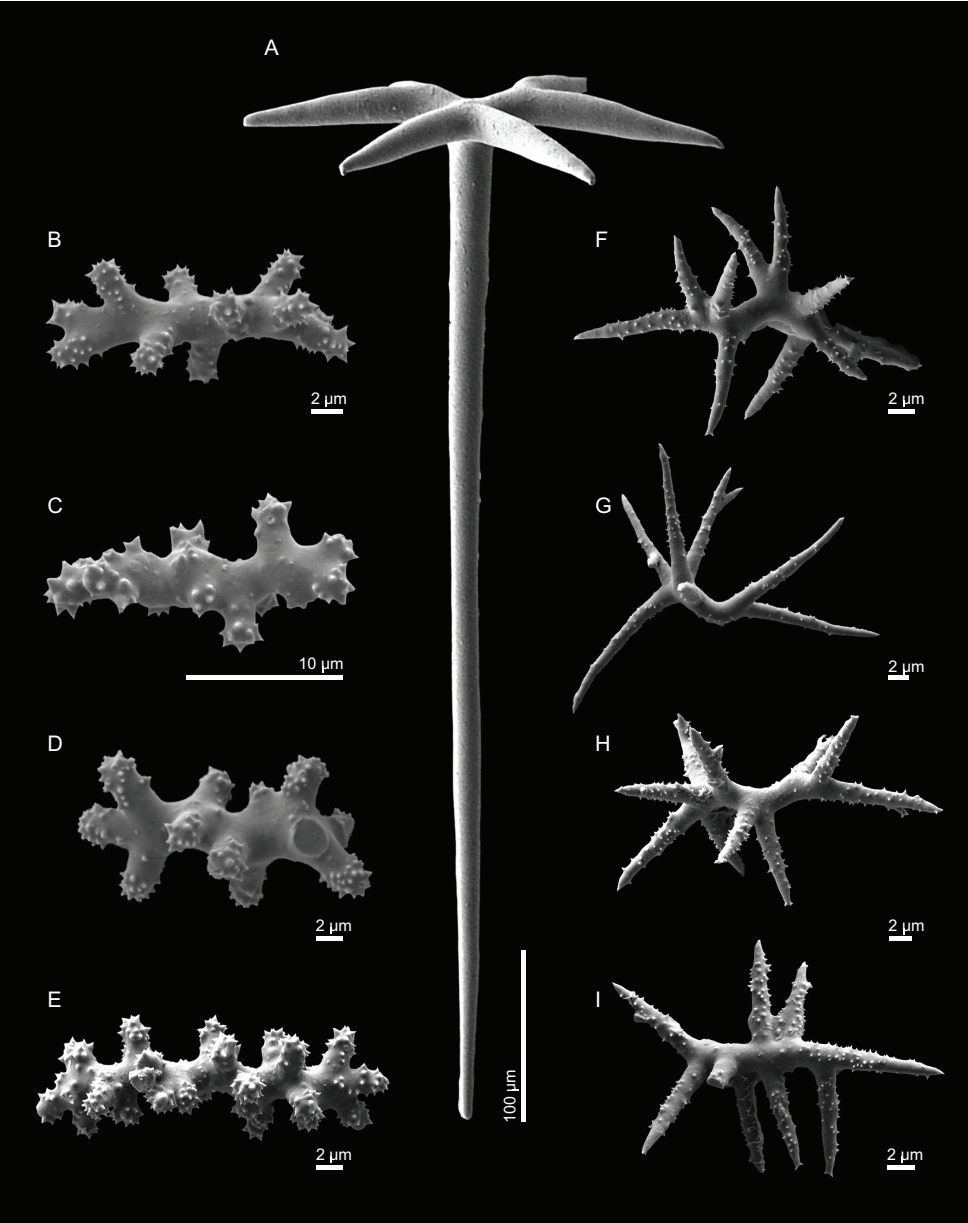
Spicules of *Neoschrammeniella pomponiae* sp. nov., holotype MNHN-IP-2008-233. (A) Smooth dichotriaene, (B)–(E) spirasters, (F)–(I) metaster.

Figures 2D, 8–9 and Table 1

urn:lsid:zoobank.org:act:2AA76193-B27E-491E-8E50-FE591786FA26

**Holotype**. MNHN-IP-2008-233 (1993-01-16, Hyères Seamount, epibenthic Warén dredge, DW182, 31°23′N, 28°54′W, 480 m, *Seamount 2* campaign).

**Comparative material examined.** *N. bowerbankii* (HBOM 003:00592, Madeira), *N. bowerbankii* (HBOM 003:00810, Madeira), *N. inaequalis* sp. nov. (holotype MNHN-IP-2008-84 and paratype MNHN-IP-2018-85, Gorringe Seamount), *N. piserai* sp. nov. (holotype MNHN-IP-2008-234, Plato Seamount).

**Diagnosis.** *Neoschrammeniella* with a cup-rounded shape and a rugose surface, fixed to the substratum by a small pedicel; dicranoclones are densely covered by numerous and ornamented tubercles with a rugose appearance.

**Description (holotype MNHN-IP-2008-233).** Large sponge, 54 mm height and 81 mm in diameter, with a small pedicel 23 mm wide; its external morphology resembles a bowl; walls are about 11 mm thick; the surfaces of the sponge are rugose, and hispid due to oxeas protruding the surface; openings are small and evenly spread on both surfaces, 40–87 μm in diameter; colour is brown in ethanol (Fig. 2D).

**Skeleton.** Ectosome is composed of a layer of dichotriaenes perpendicular to the surface that is covered by various microscleres (Figs. 8A and 8B); choanosome composed of a dense mesh of dicranoclone desmas, oxeas crossing the choanosome protruding the surface (Fig. 8A), and several microscleres spread through the skeleton.

**Spicules (holotype MNHN-IP-2008-233).**

1. **Dicranoclones**, compact, irregular and with the clones very tuberculated, 185–427–666 × 18–39–88 μm in size; rays of desmas are covered by numerous and ornamented tubercles that have a rugose appearance (Figs. 8C–8E); clones articulated into complex and intricate zygoses (Fig. 8F);
2. **Oxeas**, long, with sharp tips, 1455–1643 × 17–18 μm in size (Fig. 8A);
3. **Dichotriaenes**, with a smooth cladome, 157–274–374 μm in diameter and a long rhabdome with a blunt tip, 239–478–684 × 11–21– 37 μm in size (Fig. 9A);
4. **Spirasters**, very abundant, irregular, spiny, with short and thick arms, 10.7–18.9–35.8 μm in size (Figs. 9B–9E).
5. **Metasters**, less abundant, spiky, with long and thin arms, 16.2–27.6–39.3 μm in size (Figs. 9F–9I).

**Etymology.** Named after Dr. Shirley Pomponi from the Harbour Branch Oceanographic Institute (HBOI) in recognition of her valuable contributions to the knowledge of deep-sea sponges (including lithistids) of the North-western Atlantic Ocean and Caribbean.

**Distribution.** *N. pomponiae* sp. nov. is known from its type locality, Hyères Seamount, where it was collected at 480 m depth.

**Remarks.** The genus *Neoschrammeniella* was erected by Pisera & Lévi (2002b) to accommodate Corallistidae with smooth dichotriaenes and two to three types of microscleres. This genus is widely distributed, with records spanning the Southern Ocean, SW Pacific, Mediterranean Sea and NEA. Until now, six species were described and only one, *N. bowerbankii* (Johnson, 1863), was known to occur in the Mediterranean Sea (Pisera & Vacelet, 2011) and the NEA in the Madeira archipelago (Carvalho & Pisera, 2019; Johnson, 1863). In the present work, we described and illustrate three new species of *Neoschrammeniella*, that can mainly be distinguished by their habitus, sculpture of the desmas, presence or absence of oxeas, and, shape and size of the dichotriaenes. The external morphology of *N. pomponiae* sp. nov. resembling a bowl, contrasts with the cup-shaped to contorted lamellate masses with thick walls in *N. bowerbankii*, the flattened cup-shaped with a concave centre in *N. inaequalis* sp. nov. and the large cup-rectangular shape in *N. piserai* sp. nov. The sculpture of the desmas is also very distinct among all these species, while *N. bowerbankii* has very tuberculated dicranoclones divided into smaller and irregular lobes/tubercles (redescription in Pisera & Vacelet, 2011), *N. inaequalis* sp. nov. presents a distinct shape of desmas with vine-like appearance and few to several tubercles, *N. piserai* sp. nov. has irregular and compact dicranoclones that are usually smooth, and *N. pomponiae* sp. nov. has desmas densely covered by numerous and ornamented tubercles with a rugose appearance. Finally, *N. inaequalis* sp. nov. is the only one with very variable dichotriaenes either in size and shape, while *N. piserai* sp. nov. does not have oxeas, a type of megasclere present in the other three species.

**Family Theonellidae Lendenfeld, 1903**

**Genus *Discodermia*du Bocage, 1869**

**Synonymy.** *Collinella* Schmidt, 1879 (junior synonym); *Desmahabana* Alcolado & Gotera, 1986 (junior synonym).

**Diagnosis.** Theonellidae with discotriaenes exclusively as ectosomal megascleres and choanosomal tetraclone desmas; microscleres are acanthoxeas and acanthorhabds.

**Definition.** Polymorphic sponges, from massive irregular to cup-shaped, branched or cylindrical; ectosomal megascleres are smooth discotriaenes; choanosomal megascleres are tetraclone desmas (regular or irregular) that can be smooth or tuberculated, and oxeotes or stylotes; microscleres are acanthoxeas and acanthorhabds (Kelly, 2007; Pisera & Lévi, 2002c; Pisera & Vacelet, 2011).

**Type species.** *Dactylocalyx polydiscus* Bowerbank, 1869.

***Discodermia ramifera*Topsent, 1892**

**Figure 10:**
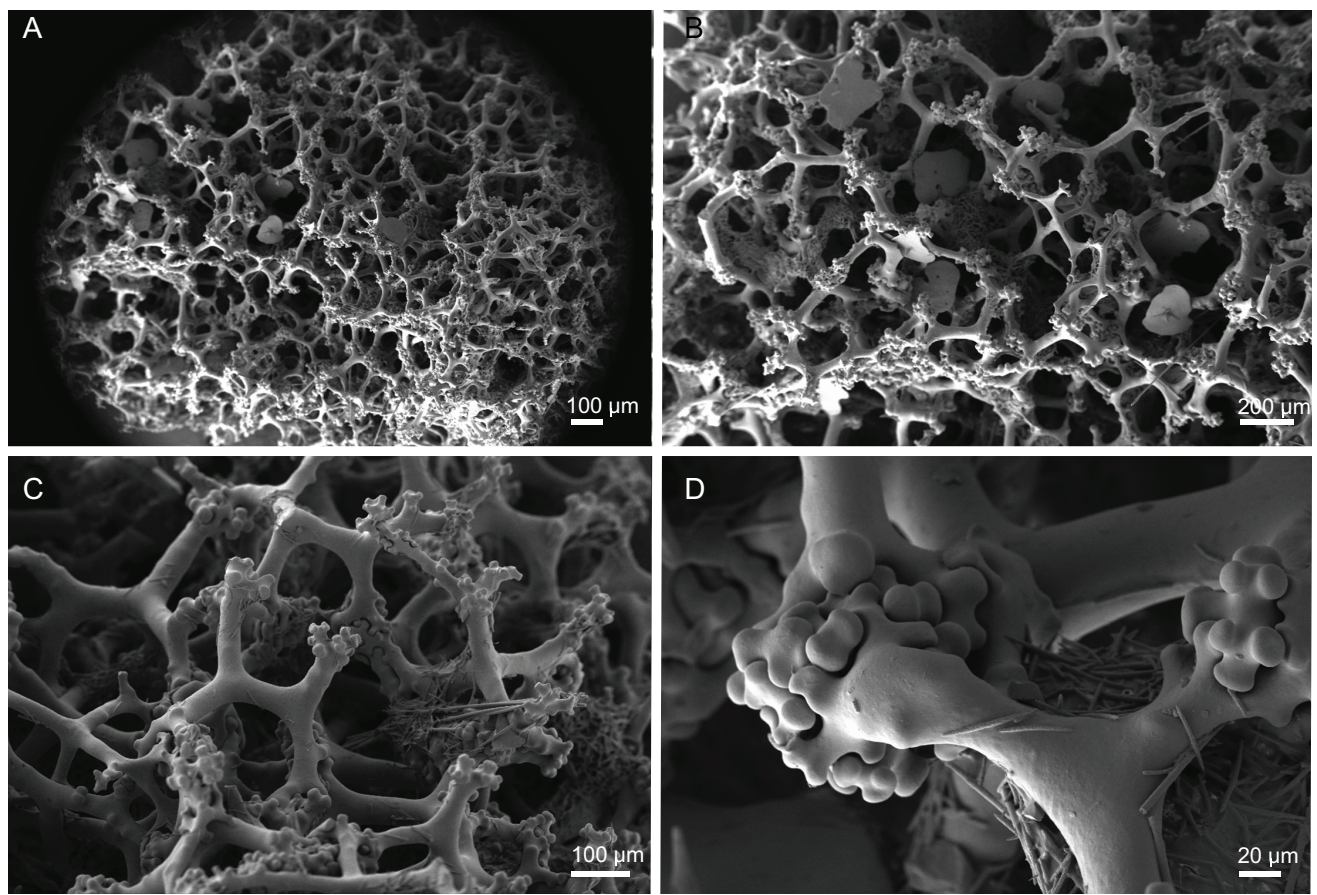
Skeleton of *Discodermia ramifera* Topsent, 1892, specimen MNHN-IP-2008-213. (A) Overview of choanosomal desmas, (B) tetraclone desmas and some discotriaenes, (C) detail of the smooth tetraclone desmas with tubercles in the zygome, (D) zygosis.

**Figure 11:**
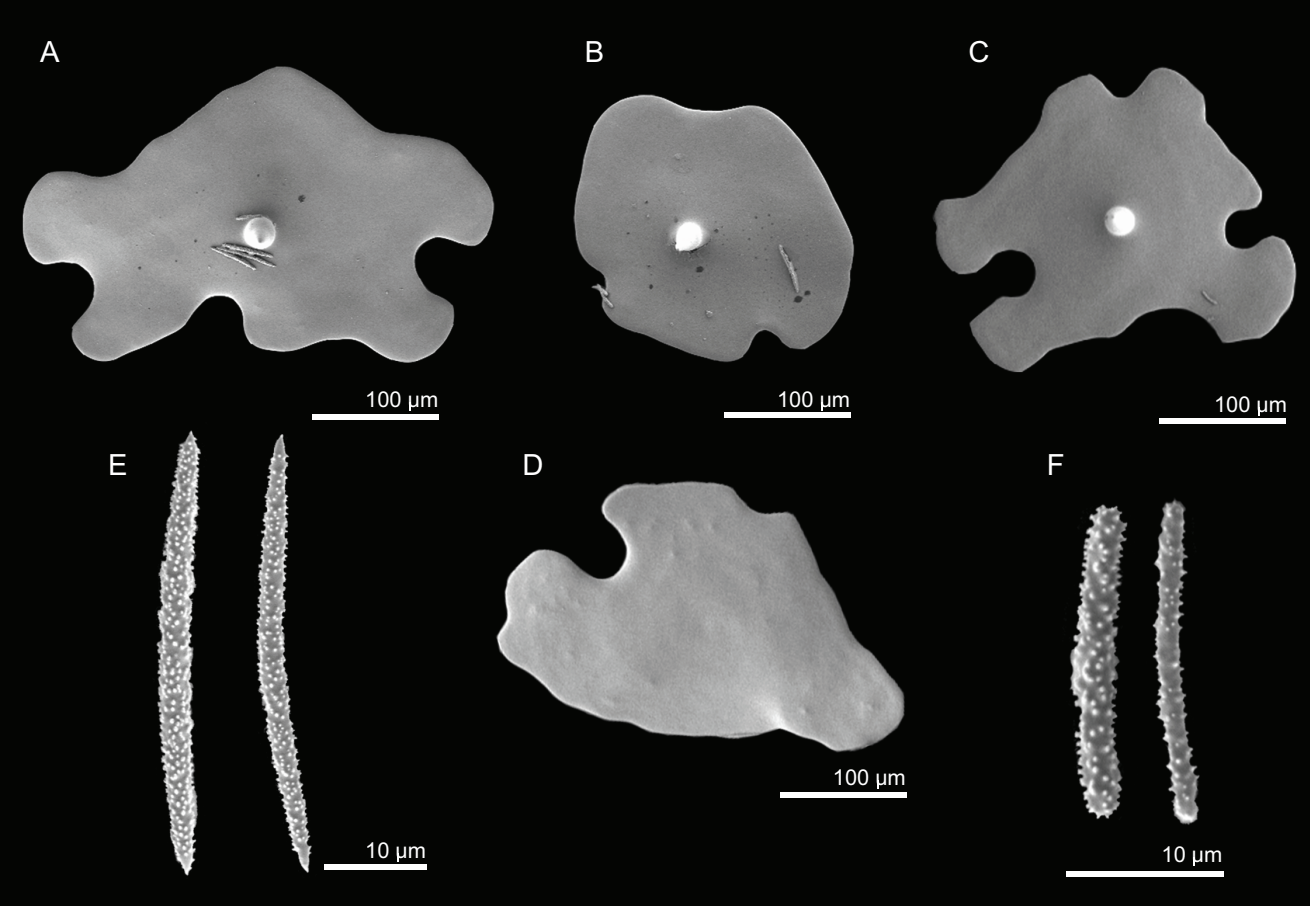
Spicules of *Discodermia ramifera* Topsent, 1892, MNHN-IP-2008-213. (A)–(C) Lower view of discotriaenes, (D) top view of discotriaene, (E) acanthomicroxeas, (F) acanthorhabds.

Figures 2E, 10–11 and Table 2

**Material examined.** MNHN-IP-2008-204 (1993-01-09, Meteor Seamount, beam trawl, CP138, 30°02′N, 28°29′W, 300 m), MNHN-IP-2008-207 (1993-01-10, Great Meteor Seamount, epibenthic dredge, DE140, 30°01′N, 28°28′W, 308 m), MNHN-IP-2008-213 (1993-01-11, Great Meteor Seamount, beam trawl, CP156, 29°56′N, 28°24′W, 320 m), MNHN-IP-2008-214 (1993-01-10, Great Meteor Seamount, beam trawl, CP144, 30°10′N, 28°29′W, 335 m). All from the *Seamount 2* campaign.

**Comparative material examined.** *Discodermia verrucosa* Topsent, 1928 (MNHN-IP-2008-205, Atlantis Seamount; MNHN-IP-2008-206, Plato Seamount; HBOM 003:00869, Madeira; HBOM 003:00870, Madeira; HBOM 003:00868, Selvagens; HBOM 003:00640, Canary Islands; RMNH6237, Selvagens), *D. kellyae* sp. nov. (holotype MNHN-IP-2008-208, Plato Seamount), *D. arbor* sp. nov. (holotype MNHN-IP-2008-211, Great Meteor Seamount).

**Diagnosis.** Small *Discodermia*, elongated to branching in shape, with smooth tetraclone desmas.

**Description (MNHN-IP-2008-213)**. Elongated and branched, small sponge, 15–29 mm high and 3–10 mm thick (Fig. 2E); surface is smooth and transparent, where it is possible to see the subdermal water canals, that gives a striated appearance to the sponge when observed under a magnifier; openings form a small elevation on the sponges’ surface; colour is beige to light yellow in ethanol.

**Skeleton.** Ectosome is composed of a layer of overlapping discotriaenes and abundant microscleres such as acanthomicroxeas and acanthorhabds, spread through this part of the skeleton; choanosomal skeleton has tetraclone desmas (Fig. 10), smooth oxeas and some microscleres spread through the entire sponge; desmas form an irregular and compact net on the choanosome but a loose mesh near the ectosome with big spaces between them; oxeas can be observed crossing the interior of the skeleton.

**Spicules (MNHN-IP-2008-213).**

1. **Tetraclone desmas**, with smooth rays (Figs. 10A–10C) and tuberculated zygoses (Fig. 10D); tubercles are generally smooth but in some cases one tubercle may be divided into various smaller tubercles; tetraclones are 182–328–470 × 24–32–48 µm in size;
2. **Discotriaenes**, very variable in shape, from round/oval to irregular and indented cladome; cladome can be flat or slightly concave, 124–160–213 µm diameter; rhabdome, short and conical, 23–32–40 µm × 8–10–14 µm in size (Figs. 11A–11D).
3. **Oxeas**, long, smooth with rounded extremities (Fig. 10C); the vast majority of oxeas were broken, thus measurements of these megascleres are not presented here.
4. **Acanthomicroxeas**, slightly curved with pointed ends, rarely centrotylotes, 23–28–33 × 1.0–1.5–1.8 µm in size (Fig. 11E).
5. **Acanthorhabds**, similar to microxeas with the exception they are smaller and have rounded tips, 3.9–10.3–13.9 × 1.1–1.4–1.9 µm in size (Fig. 11F).

**Distribution.** Specimens were collected at the Great Meteor Seamount between 300 and 335 m depth.

**Remarks.** *D. ramifera* was described by Topsent (1892) from material collected in the Azores (318 m depth), and later re-collected in the same archipelago at 98 m depth (Topsent, 1904). So far, these were the only records in the North Atlantic. Here we discover for the first time the presence of this species in the Great Meteor seamount (between 300 and 335 m depth). The specimens analysed in this work have a similar external morphology compared to the ones described by Topsent (i.e., small, elongated to branching sponge with finger-like extensions), and similar spicule composition. However, the spicules’ sizes are in general smaller from those presented in the original description (Table 2). Discotriaenes have a smaller cladome, 124–213 µm in the analysed material *versus* the 300 µm in diameter in the original description; acanthomicroxeas (22.8–32.6 µm *vs* 40–45 µm long) and acanthorhabds are also smaller (3.9–13.9 µm *vs* 20–25 µm long), but see Discussion for more details on these differences.

**Table 3:** Comparative table of external morphology and spicular micrometries of all *Macandrewia* species recorded in the North Atlantic. Spicule measurements (*n* = 30 unless stated otherwise) are presented as minimum–mean–maximum. Data compiled from the original descriptions, or subsequent re-descriptions of type material (marked with numbers).

	Habitus	Size	Desmas	Phyllotriaenes	Oxeas	Microxeas	Locality
^1^*Macandrewia azorica* Gray, 1859 (Holotype BMNH 1851.7.28.16)	Cyathiform to flabellate, with a short stem and undulating rounded margins; outer surface smooth, with small, irregular but evenly distributed pores, 37–58 µm in diameter	120 × 120 mm with a short stem, 30 mm long; walls 6–9 mm thick	Smooth, complex, strongly branched at the end with a loose terminal articulation, 255–438–724 × 8.5–19.0–30.8 µm in size (*n* = 22)*	Cladome: with strongly incised clades, 297–363–456 µm (*n* = 11) in diameter; rhabdome: conic and short, 157–163–167 × 17.5–19.9–22.2 µm in size (*n* = 3)*	Small, fusiform and thick, 532–652–780 × 10.5–15.1–19.4 µm (*n* = 8)*	Very common, fusiform, 38–55–96 × 2.5–3.9–7.9 µm*	S. Miguel island, Azores (depth unknown)
*M*. cf. *azorica* (MNHN-IP-2008-220)	Flabellate to undulate masses with thin lamellas; smooth surfaces; colour beige to light brown	67 × 50 mm in size; walls are rounded and undulate, 3–5 mm thick	212–281–343 × 16–34–51 µm (*n* = 24)	Cladome: very indented, 194–267–333 µm (*n* = 20); rhabdome: 62–99–129 × 11.6–14.4–17.8 µm (*n* = 12)	215–246–301 × 6.8–7.8–9.1 µm (*n* = 4)	33.3–55.0–83.6 × 2.5–3.9–5.1 µm	Atlantis Seamount (420 m depth)
^2^*M. clavatella* (Schmidt, 1870) (unknown type)	Obconic, seated on a short pedicel, summit flattened or depressed, or convexly rounded, bearing several oscules 0.25–1.0 mm in diameter; pores 0.035–0.04 mm in diameter, dispersed over the sides of the sponge; colour greyish-white	–	Usually smooth, 50–100 × 14–19 µm in size; tubercles are short and well rounded	130 µm in length.	Fusiform, slender, 390 × 13 µm in size	Fusiform, sometimes with an ellipsoidal centrotylus, usually curved, 55 × 4 µm, in size	Florida, U.S.A. (278–494 m depth)
^3^*M. robusta* Topsent, 1904 (unknow type)	Very hard sponges, simple in shape, with thick and short pedicel; top of the sponge can be curved or slightly depressed; water canals visible	–	Monocrepid, smooth, with short and thick tubercles, forming a very strong zygosis; 40 µm diameter	Cladome: scarcely ramified with very indented edges, 165–230 µm; rhabdome: conic, thick, 100–140 × 28–33 µm	Fusiform, slightly curved, 330–400 × 8–12 µm	Smooth, curve, thickened in the center, 20–60 × 4–7 µm	Azores (1,165 m depth)
*M. robusta* Topsent, 1904 (MNHN-IP-2008-216)	Ficiform to globular in shape, with a short and thick pedicel; surface smooth with openings and water canals visible to the naked eye; colour beige to light brown	18–20 mm high, 14–22 mm in diameter	248–362 (*n* = 2) × 17–22–31 µm (*n* = 22)	Cladome: variable in shape, indented on the edges, 154–228–309 µm (*n* = 20); rhabdome: 46–91–141 × 13–19–25 µm (*n* = 10)	203–329 × 7.2–8.2 µm (*n* = 3)	34.6–57.4–79.2 × 3.1–4.7–6.9 µm	Hyéres Seamount (705 m depth)
^3^*M. ramosa* Topsent, 1904 (unknow type)	Encrusting with an extensive base where it stands two or three trunks that are slender, subcylindrical, with the top divided into short and obtuse branches	–	Zygosis interlocks with rounded tubercles	Cladome: large, foliated, thin, fully divided, 80–120 µm; rhabdome: conic, 75 × 13 µm	Fusiform, 200–300 × 5–6 µm	Smooth, slightly curved, thickened in the center, 50–65 × 4–5 µm	Azores (1,360 m depth)
*M. schusterae* sp. nov. (Holotype MNHN-IP-2018-87)	Foliate macandrewia with thick and contorted lamellas, usually attached to the substrate by a large pedicel; surface are smooth; colour light brown to white	94 mm height, 142 mm wide at the top and 45 mm wide at the base; lamellas are 7–10 mm thick	Compact and irregular skeleton, with smooth, short and blunt clones, 301–386–463 × 10.2–19.9–39.2 µm (*n* = 27)	Cladome: incised especially in the edges, 177–304–420 µm; rhabdome: 67–119–178 × 13–21–26 µm (*n* = 13)	Smooth, round tips, 263–437–620 × 8.1–12.4–16.0 µm (*n* = 20)	Smooth, round tips, 43.8–67.9–95.2 × 2.5–4.3–7.7 µm	Gorringe Seamount (605–675 m depth)
*M. schusterae* sp. nov. (Paratype MNHN-IP-2018-88)	Foliate macandrewia with thick lamellas	107 mm height, 22 mm wide at the base and 145 mm at the top; lamellas are 7–9 mm thick	326–449–612 × 13.6–27.7–49.6 µm (*n* = 24)	Cladome: 187–325–457 µm; rhabdome: 94–138–207 × 12–22–31 µm (*n* = 21)	302–466–563 × 5.3–10.0–13.3 µm (*n* = 21)	53.6–74.0–109.8 × 3.8–6.0–8.3 µm	Gorringe Seamount (605–675 m depth)
*M. minima* sp. nov. (Holotype MNHN-IP-2008-222)	Round shape with a very small pedicel, smooth surface, pores are visible and scattered on the top; colour is white	15–20 mm height, 17–20 mm wide, 16–17 mm in diameter; base 6 mm wide	Compact and irregular skeleton; clones are robust, usually smooth in the center, 268–318–348 (*n* = 10) × 7–29–50 µm	Cladome: incised ornamented by tubercles, 136–222–284 µm; rhabdome: conic and short, 58–99–136 × 14–19–25 µm (*n* = 13)	Smooth, 197–251–316 × 7.5–11.9–16.2 µm (*n* = 4)	Often curved, tips are blunt, 25.9–48.3–74.2 × 3.1–4.4–7.0 µm	Great Meteor Seamount (615 m depth)

**Notes:**

1 Pisera & Lévi (2002e).

2 This description was taken from Sollas (1888) since the species was poorly described in the original description and spicules’ measurements were not given.

3 Topsent (1904).

* Measurements of spicules from the holotype presented here, were measured for this study, they were not taken from the redescription of the holotype.

‘–’ no information/not mentioned.

***Discodermia* cf. *ramifera*Topsent, 1892**

**Material.** MNHN-IP-2008-210 (1993-02-02, Atlantis Seamount, epibenthic Warén dredge, DW258, 34°00′N, 30°12′W, 420 m, *Seamount 2* campaign).

**Comparative material examined.** *D. ramifera* (MNHN-IP-2008-204, Great Meteor Seamount; MNHN-IP-2008-207, Great Meteor Seamount; MNHN-IP-2008-213, Great Meteor Seamount; MNHN-IP-2008-214, Great Meteor Seamount), *Discodermia verrucosa* Topsent, 1928 (MNHN-IP-2008-205, Atlantis Seamount; MNHN-IP-2008-206, Plato Seamount; HBOM 003:00869, Madeira; HBOM 003:00870, Madeira; HBOM 003:00868, Selvagens; HBOM 003:00640, Canary Islands; RMNH6237, Selvagens), *D. kellyae* sp. nov. (holotype MNHN-IP-2008-208, Plato Seamount), *D. arbor* sp. nov. (holotype MNHN-IP-2008-211, Great Meteor Seamount).

**Description (MNHN-IP-2008-210).** Small fragment, 20 × 10 mm in size, of elongated shape, with a smooth surface; subdermal water canals are visible, giving a striated appearance to the sponge; colour is beige in ethanol.

**Skeleton.** Ectosomal skeleton is formed by a layer of overlapped discotriaenes, and several microscleres spread through the surface; choanosome is formed by irregular tetraclone desmas, oxeas crossing the interior of the sponge and numerous microscleres spread through the interior of the sponge.

**Spicules (MNHN-IP-2008-210).**

1. **Tetraclone desmas**, irregular, with smooth clones and very tuberculated on the extremities, 400–455–534 × 30–51–82 µm in size; tubercles are smooth;
2. **Discotriaenes**, cladome varies from oval to indented in shape, usually flat, 195–328–560 µm in diameter; rhabdome is short, conical, with a blunt tip, 20–42–68 × 9.5–20.3–37.9 µm in size;
3. **Oxeas**, are present, but all of them were broken;
4. **Acanthomicroxeas**, very abundant, spinous, with sharp tips, 24.6–39.0–59.8 × 1.8–3.3–5.4 µm in size;
5. **Acanthorhabds**, small, abundant, spinous, with rounded extremities, 15.2–20.2–24.2 × 2.1–2.9–4.4 µm.

**Distribution.** This specimen was collected in the Atlantis Seamount at 420 m depth.

**Remarks.** Although the external morphology, type of spicules and desma ornamentation are in agreement with the description of *D. ramifera*, the spicules sizes of this specimen are significantly larger when compared to the ones found in the Great Meteor (Table 2). For this reason, we consider this species as *D*. cf. *ramifera*.

***Discodermia verrucosa*Topsent, 1928**

**Figure 12:**
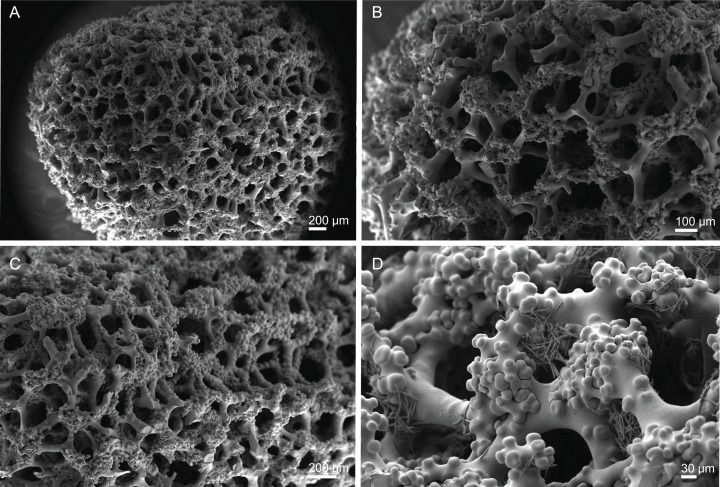
Skeleton of *Discodermia verrucosa* Topsent, 1928, specimen MNHN-IP-2008-205. (A) Overview of tetraclone desmas, (B) and (C) irregular and compact net of tetraclone desmas, (D) detail of the strongly tuberculated zygosis.

**Figure 13:**
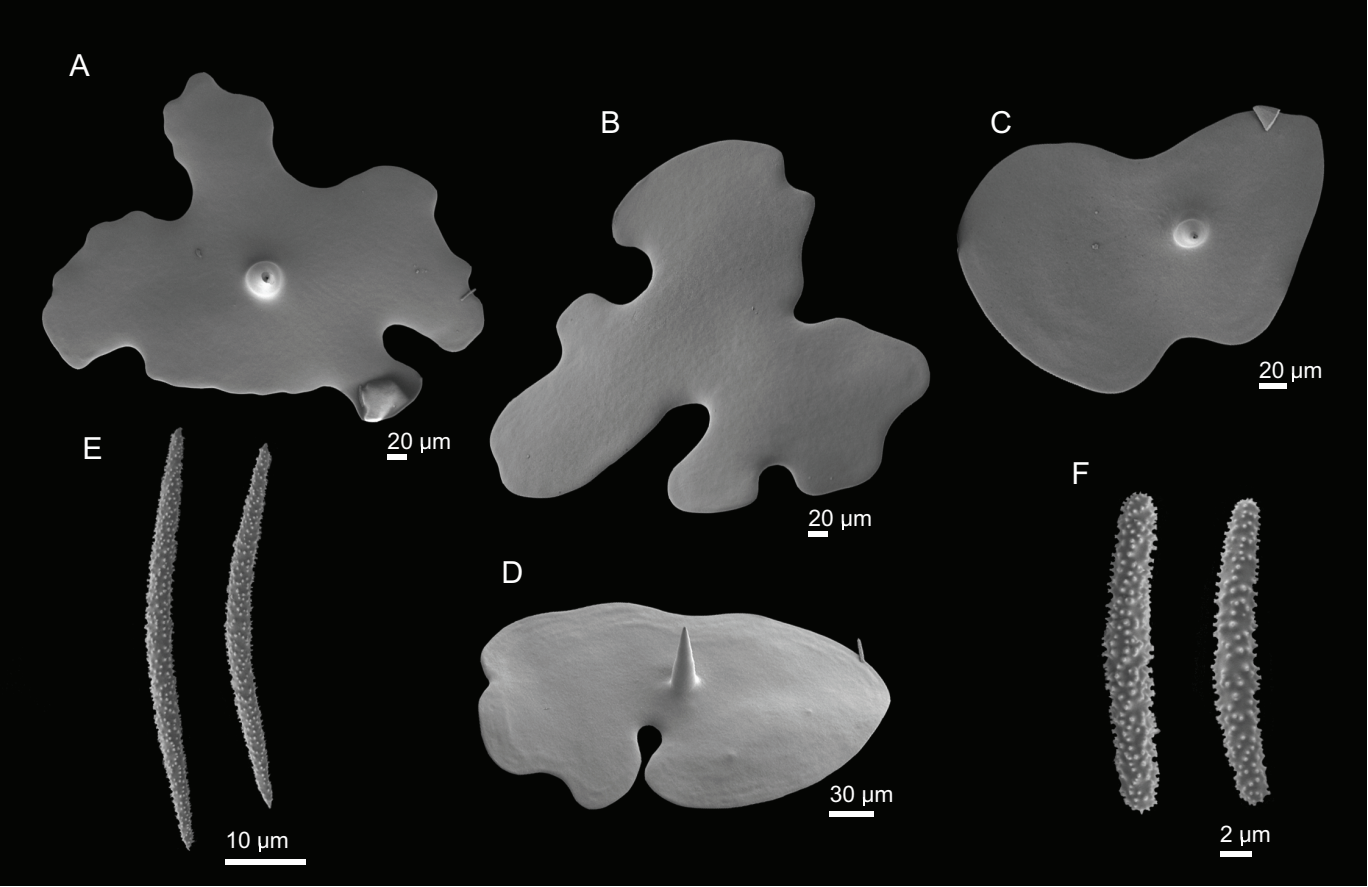
Spicules of *Discodermia verrucosa* Topsent, 1928, specimen MNHN-IP-2008-205. (A)–(D) Upper and lower view of discotriaenes, (E) acanthomicroxeas, (F) acanthorhabds.

Figures 2F, 12–13 and Table 2

**Material examined.** MNHN-IP-2008-205 (1993-02-02, Atlantis Seamount, beam trawl, CP257, 34°04′N, 30°15′W, 338 m), MNHN-IP-2008-206 (1993-02-01, Plato Seamount, epibenthic Warén dredge, DW246, 33°14′N, 29°36′W, 520 m). All from *Seamount 2* campaign.

**Comparative material examined.** *D. ramifera* (MNHN-IP-2008-204, Great Meteor Seamount; MNHN-IP-2008-207, Great Meteor Seamount; MNHN-IP-2008-213, Great Meteor Seamount; MNHN-IP-2008-214, Great Meteor Seamount), *D. kellyae* sp. nov. (holotype MNHN-IP-2008-208, Plato Seamount), *D. arbor* sp. nov. (holotype MNHN-IP-2008-211, Great Meteor Seamount).

**Diagnosis.** Cup-shaped to spherical sponges with numerous warts/protuberances, and extremely tuberculated tetraclone desmas (emended after Topsent, 1928).

**Description (MNHN-IP-2008-205)**. Spherical polymorphic sponge with several round protuberances, 15–20 mm high and 12–13 mm wide, with a rough surface (Fig. 2F); pores cannot be seen with naked eye; colour varies from whitish to light brown in ethanol.

**Skeleton.** Ectosome composed of a compact layer of discotriaenes, usually overlapping each other, numerous microscleres (acanthomicroxeas and acanthorhabds) spread through the surface, and oxeas perforating the sponges’ surface; occasionally, bundles of oxeas can be observed; choanosome with strongly tuberculated and compact tetraclone desmas (Fig. 12), forming an irregular net with dispersed microscleres in the interior of the sponge.

**Spicules (MNHN-IP-2008-205).**

1. **Tetraclone desmas**, large, robust, mostly with tubercles spread through the entire clone, although some parts can be smooth, 106–170–278 × 19–34–46 µm in size (Figs. 12A–12C); zygoses very robust and extremely tuberculate (Fig. 12D);
2. **Discotriaenes**, irregular in shape, from round to oval, often indented (Figs. 13A–13D); cladome smooth, slightly concave, 102–153–222 µm in diameter; rhabdome is short with a conical shape, 15–25–47 × 5–8–13 µm (Fig. 13D);
3. **Oxeas**, long, smooth with rounded ends; length not presented here because they were all broken due to their large size.
4. **Acanthomicroxeas**, spinous, slightly curved with pointed ends, 22.8–35.2–53.5 × 1.3–2.2–3.9 µm (Fig. 13E).
5. **Acanthorhabds**, cylindrical, spinous, with blunt tips, 7.5–12.9–19.0 × 1.2–1.6–3.0 µm in size (Fig. 13F).

**Distribution.** Specimens of *D. verrucosa* were found in Atlantis and Plato Seamounts between 338 and 580 m depth.

**Remarks.** *Discodermia verrucosa* was first found in the Canary Islands and described by Topsent (1928). The species differs from the *D. ramifera* on the habitus and sculpture of desmas. *D. verrucosa* has a cup to spherical shape with several rounded protuberances/warts and strongly tuberculated tetraclones. On the other hand, *D. ramifera* has an elongated to branching shape and smooth tetraclone desmas only tuberculated in the extremities. The specimens analysed in this study overall match the description of *D. verrucosa*, apart from two differences: (1) the discotriaenes are much smaller and (2) the microscleres present a wider size range when compared to the original description (see Table 2).

***Discodermia arbor* sp. nov.**

**Figure 14:**
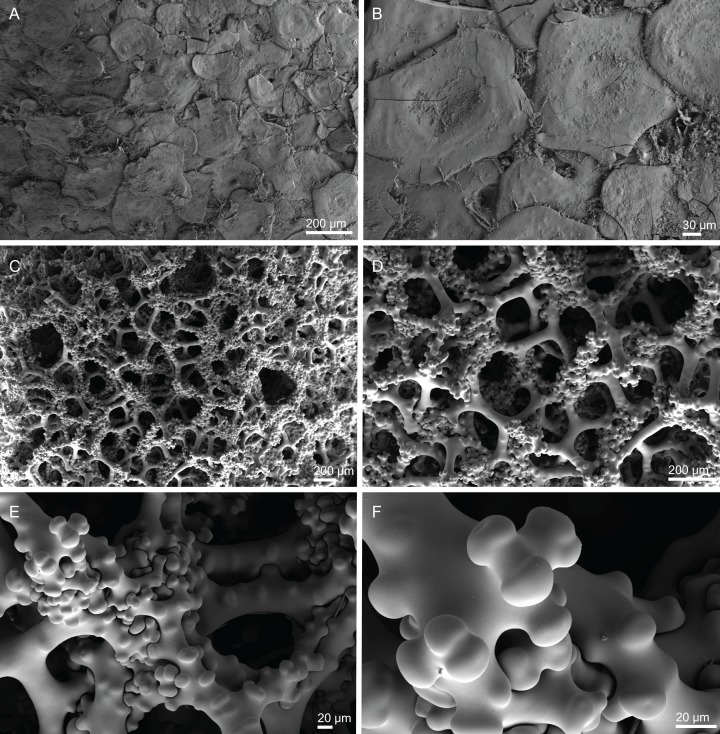
Surface and skeleton of *Discodermia arbor* sp. nov., holotype MNHN-IP-2008-211. (A) Overview of the surface, (B) detail of the surface showing the overlapped discotriaenes, (C) overview of choanosomal tetraclone desmas, (D) detail of tetraclone desmas, (E) complex zygoses between several desmas, (F) detail of the desmas ornamentation, showing smooth tubercles.

**Figure 15:**
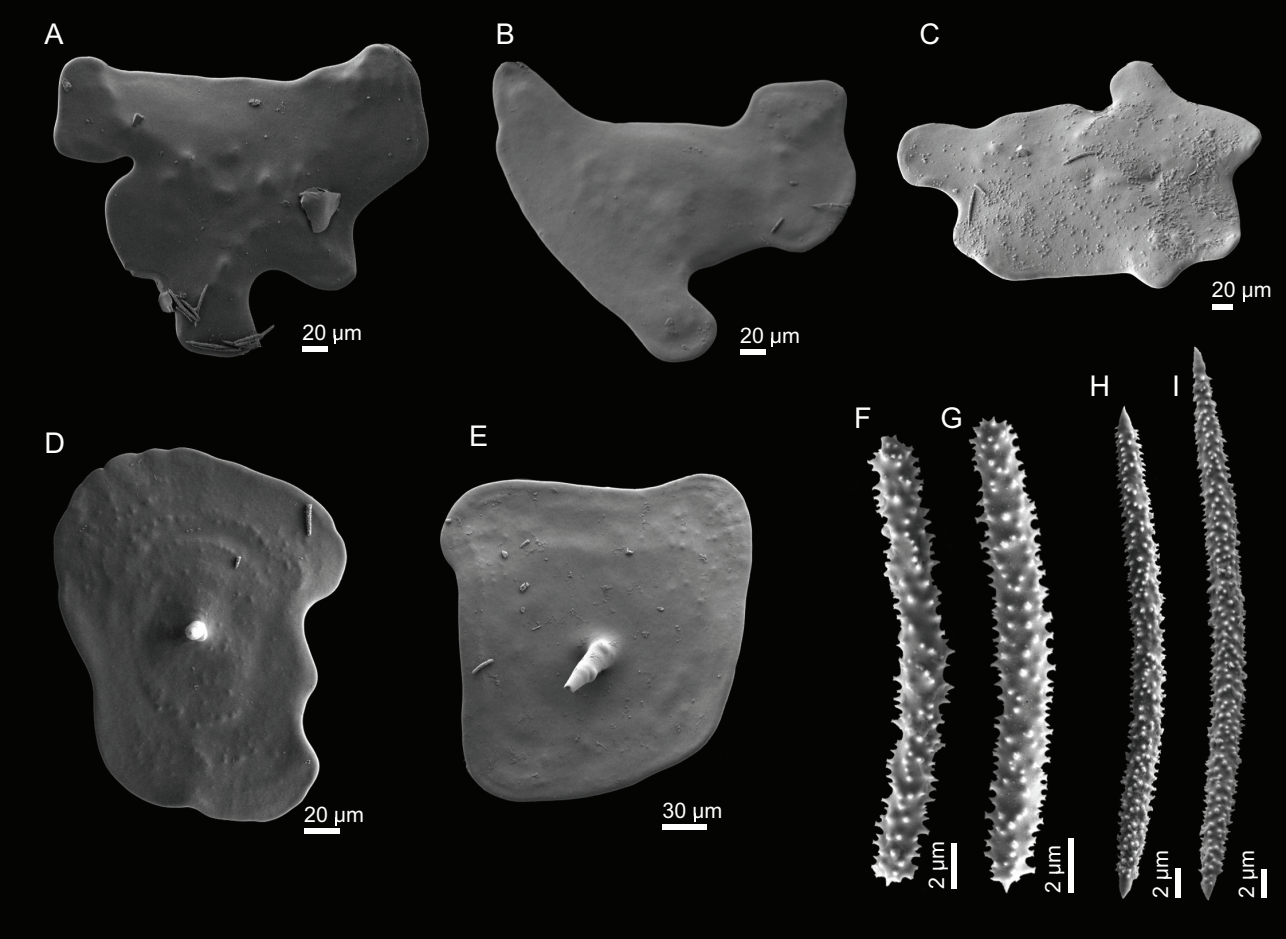
Spicules of *Discodermia arbor* sp. nov., holotype MNHN-IP-2008-211. (A)–(C) Top view of discotriaenes, (D) and (E) bottom view of discotriaenes showing the rhabdome, (F) and (G) acanthorhabds, (H) and (I) acanthomicroxeas.

Figures 2G, 14–15 and Table 2

Urn:lsid:zoobank.org:act:7A732A92-8D8B-4D73-97B1-CD53E9494121

**Holotype.** MHNH-IP-2008-211 (1993-01-11, Great Meteor Seamount, beam trawl, DW159, 29°44′N, 28°20′W, 330 m, *Seamount 2* campaign).

**Comparative material examined.** *D. ramifera* (MNHN-IP-2008-204, Great Meteor Seamount; MNHN-IP-2008-207, Great Meteor Seamount; MNHN-IP-2008-213, Great Meteor Seamount; MNHN-IP-2008-214, Great Meteor Seamount), *D. verrucosa* (MNHN-IP-2008-205, Atlantis Seamount; MNHN-IP-2008-206, Plato Seamount; HBOM 003:00869, Madeira; HBOM 003:00870, Madeira; HBOM 003:00868, Selvagens; HBOM 003:00640, Canary Islands; RMNH6237, Selvagens), *D. kellyae* sp. nov. (holotype MNHN-IP-2008-208, Plato Seamount).

**Diagnosis.** *Discodermia* of tree-like appearance; discotriaenes vary from square to circular shape and can also be indented.

**Description (holotype MHNH-IP-2008-211).** *Discodermia* of tree-like appearance (Fig. 2G), with a relatively long stem, 15 mm, where it extends on top into three branches; the stem is wider at the base, 12 mm, and thinner on top, 7.5 mm; branches are irregular and 13–28 mm long; surface is smooth but some rugosities/protuberances are visible; full sponge length is 58 mm; the sponge was attached to the substrate by the stem; colour is beige in ethanol.

**Skeleton.** Ectosome has a layer of overlapped discotriaenes of variables sizes (Figs. 14A and 14B) with numerous microscleres beneath them; choanosome is composed of an irregular net of tetraclone desmas (Figs. 14C and 14D) and spread microscleres; near the surface, tetraclones are more intricate, rugose, with very complex and strong zygoses near the water canals (Fig. 14C); in the interior part of the sponge, the tetraclones still form an intricate and irregular net, but there is more space between the desmas.

**Spicules (holotype MHNH-IP-2008-211).**

1. **Tetraclone desmas**, thick, irregular, ornamentation varies according with the location of the desmas, *i.e*, near the surface the clones have usually tubercles spread through the entire ray (Figs. 14C and 14D) while in the interior they are smoother; tubercles on the zygome are smooth and sometimes subdivided (Fig. 14F); zygoses are very complex and robust (Figs. 14E and 14F), giving a hard consistency to this sponge; tetraclones are 181–392–567 × 15–36–56 µm in size;
2. **Discotriaenes**, very variable in shape, from “square” to “circular” shape, or with indented cladomes (Figs. 15A–15E); cladome is smooth with some protuberances, 148–256–396 µm in diameter; rhabdome is relatively short with blunt tips, 34–53–71 × 15–21–24 µm in size;
3. **Acanthomicroxeas**, slightly curved, covered by numerous spines with sharp tips, 24.1–35.1–50.1 × 1.4–2.3–3.5 µm in size (Figs. 15H and 15I);
4. **Acanthorhabds**, small, with several spines, usually with blunt tips, but they can also be unequal and have a sharp tip in one of the extremities, 6.7–16.1–25.9 × 1.1–2.2–4.3 µm in size (Figs. 15F and 15G);

**Etymology.** From the latin *arbor* = tree; this *Discodermia* looks like a small tree.

**Distribution.** *D. arbor* sp. nov. is only know from the Great Meteor Seamount, where it was found at 330 m depth.

**Remarks.** *Discodermia arbor* sp. nov. is here described as a new species constituting the eighth *Discodermia* species reported to the North Atlantic and Mediterranean Sea. Its tree-like shape is very distinct from the other *Discodermia* spp. recorded for this area. Besides that, this species does not have oxeas, a spicule type that was reported in all *Discodermia* species in the North Atlantic except for *D. polymorpha* from the Mediterranean Sea. Although *D. arbor* sp. nov. shares the absence of oxeas with *D. polymorpha*, they have very different habitus, desmas ornamentation and size of microscleres (but see Remarks under *D. kellyae* sp. nov. for a more detailed comparison of all *Discodermia* species in the North Atlantic and Mediterranean Sea).

***Discodermia kellyae* sp. nov.**

**Figure 16:**
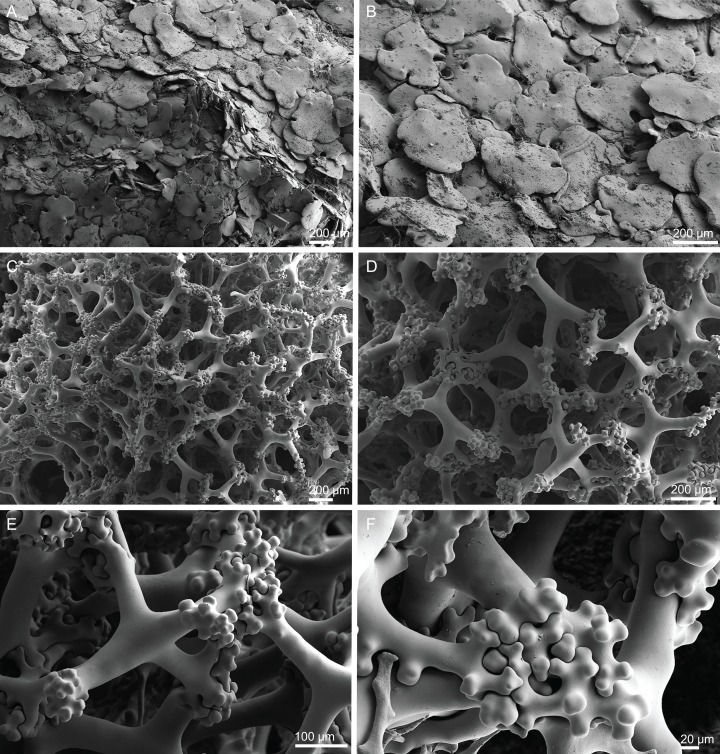
Surface and skeleton of *Discodermia kellyae* sp. nov., holotype MNHN-IP-2008-208. (A) Overview of the surface, (B) overlapped discotriaenes on the surface, (C) overview of choanosomal tetraclone desmas, (D) tetraclone demas, (E) detail of a tetraclone desma showing their sculpture and ornamentation, (F) detail of the zygosis.

**Figure 17:**
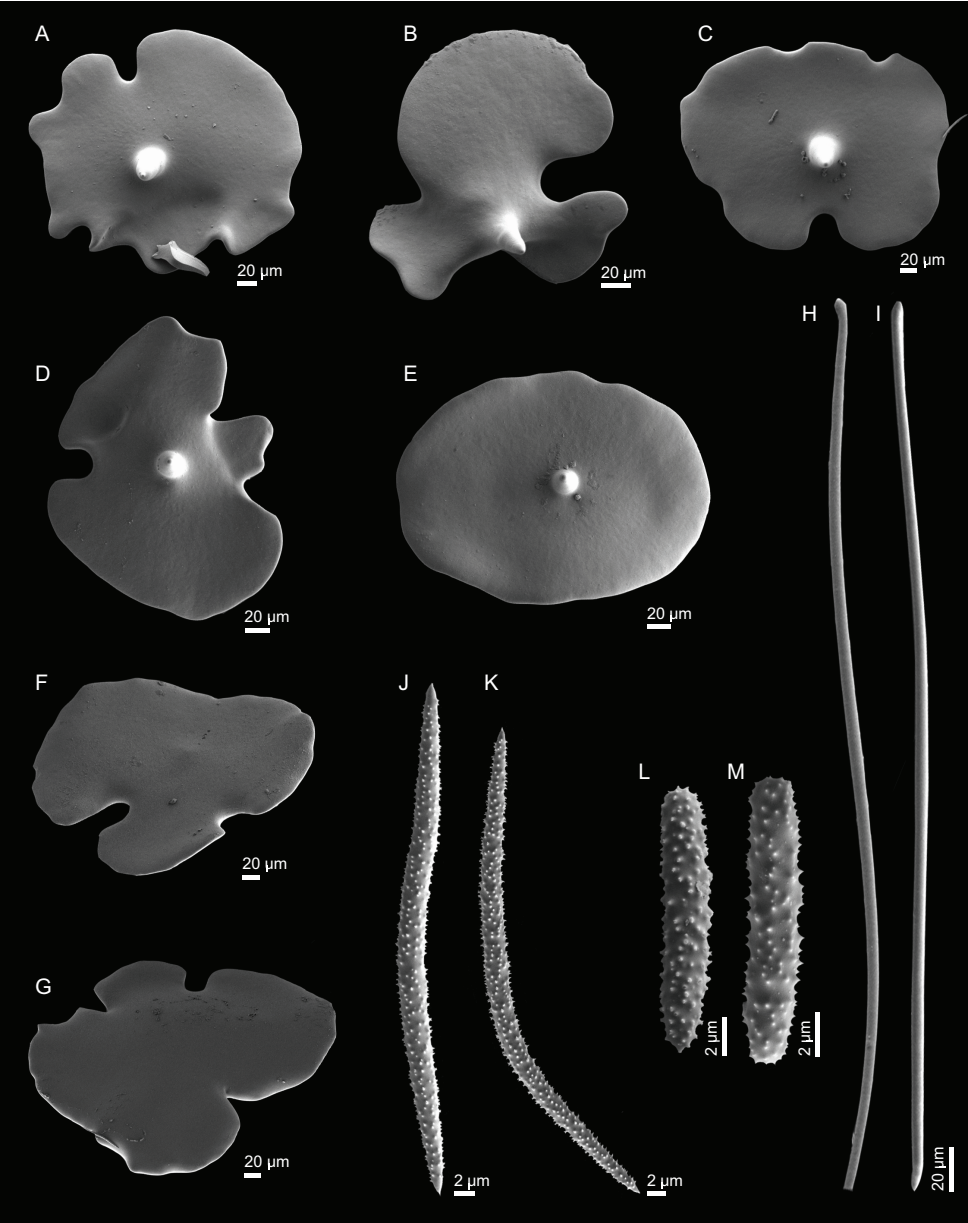
Spicules of *Discodermia kellyae* sp. nov., holotype MNHN-IP-2008-208. (A)–(E) Bottom view of discotriaenes, (F and G) top view of discotriaenes, (H and I) strongyles, (J and K) acanthomicroxeas, (L and M) acanthorhabds.

Figures 2H, 16–17 and Table 2

urn:lsid:zoobank.org:act:E7A06142-4AF7-404E-B369-B30240ADE5F4

**Holotype**. MNHN-IP-2008-208 (1993-02-03, Plato Seamount, beam trawl, DW247, 33°14’N, 29°35’W, 580 m, *Seamount 2* campaign).

**Comparative material examined.** *D. ramifera* (MNHN-IP-2008-204, Great Meteor Seamount; MNHN-IP-2008-207, Great Meteor Seamount; MNHN-IP-2008-213, Great Meteor Seamount; MNHN-IP-2008-214, Great Meteor Seamount), *D. verrucosa* (MNHN-IP-2008-205, Atlantis Seamount; MNHN-IP-2008-206, Plato Seamount; HBOM 003:00869, Madeira; HBOM 003:00870, Madeira; HBOM 003:00868, Selvagens; HBOM 003:00640, Canary Islands; RMNH6237, Selvagens), *D. arbor* sp. nov. (holotype MNHN-IP-2008-211, Great Meteor Seamount).

**Diagnosis.** Massive, spherical, irregular, *Discodermia* of bulb appearance, with smooth tetraclone desmas.

**Description (holotype MNHN-IP-2008-208).** Massive sponge, irregular appearance, with large protuberances of round shape, 53 mm high and 31 mm wide; surface is irregular with a rugose appearance; the basal part of the sponge is not evident, since there is no obvious mark in the sponge that shows where it was attached to the substrate; colour is beige to light brown in alcohol (Fig. 2H).

**Skeleton.** Ectosome is composed of a layer of overlapped discotriaenes (Figs. 16A and 16B) of different sizes with several microscleres spread through the surface; openings are surrounded by these microscleres; choanosome is composed by an irregular net of tetraclone desmas (Figs. 16C and 16D), forming large areas between them, usually near the ectosome; the rays of the tetraclones articulate into a complex zygosis; several microscleres and some strongyles are spread loosely in the choanosome.

**Spicules (holotype MNHN-IP-2008-208).**

1. **Tetraclone desmas**, compact, irregular, with smooth and thick clones, 112–338–589 × 20–42–76 µm in size (Figs. 16C and 16D); the termination of the clones has several tubercles, resulting in very complex and large zygoses (Figs. 16D–16F); tubercles of the clones are smooth (Fig. 16F).
2. **Discotriaenes**, irregular, with diverse shapes and sizes; cladomes vary from oval to indented discs, and they are either flat or concave, 121–289–425 µm in diameter (Figs. 16A, 16B and 17A–17G); rhabdome is also very variable in size, 36–78–119 × 13–30–44 µm, with a blunt or sharp tip.
3. **Strongyles**, with one of the tips rounded and the other one sharp, sometimes resembling a crochet needle, 418–444 × 6.0–7.9 µm in size (Figs. 17H and 17I);
4. **Acanthomicroxeas**, very abundant, long, straight to curved, covered by numerous spines, with sharp tips, 16.7–43.2–66.5 × 1.5–2.5–3.7 µm in size (Figs. 17J and 17K);
5. **Acanthorhabds**, very abundant, with blunt tips, covered by numerous spines, very variable in size, 5.3–13.3–24.9 × 1.2–2.1–3.7 µm (Figs. 17L and 17M).

**Etymology.** Named after Dr. Michelle Kelly from the National Institute of Water and Atmospheric Research (NIWA) in recognition of her work on taxonomy and systematics of Porifera, particularly on lithistid demosponges of New Zealand.

**Distribution.** *D. kellyae* sp. nov. is only known from its type locality, the Plato Seamount at 580 m depth.

**Remarks.** The identification of species belonging to the genus *Discodermia* is particularly challenging due to the few and very variable morphological characters used for the distinction of species (Pisera & Vacelet, 2011). Moreover, for some species we are limited to the original descriptions where detailed information of skeletal composition and spicule sizes, or images are lacking.

In the North Atlantic and Mediterranean Sea, a total of nine species have been described, including the two described species in this study (Table 2). Despite the high plasticity of morphological characters, the main differences between species are (1) habitus, (2) the sculpture and size of the desmas, (3) size and shape of the discotriaenes, and (4) size and shape of the microscleres. We propose *D. kellyae* sp. nov. as a new species based on (1) the habitus of this sponge: the polymorphic sponge of bulb appearance contrasts with the massively encrusting shape of *D. adhaerens*, the spherical to irregular masses in *D. polymorpha*, the cup-shaped with numerous warts/protuberances in *D. verrucosa*, the elongated with several finger-like extensions in *D. ramifera*, the tree-like shape of *D. arbor*, the cluster of knobby fingers in *D. dissoluta* and the irregular mushroom shape of *D. polydiscus*; (2) tetraclones of *D. kellyae* sp. nov. have similar ornamentation to the ones found in *D. ramifera* (tetraclones with smooth clones that are tuberculated in the zygomes), however, they are more compact and thicker (24–32–48 µm *vs* 20–42–76 µm) resembling the ones present in *D. verrucosa*; the other species have slender and smooth desmas without strong/complex zygoses; (3) the intraspecific size range of discotriaenes is usually wide, and similar between the different species, but in *D. kellyae* sp. nov. the size range of the cladomes is very large, 121–425 µm, and this can only be observed in *D. verrucosa* (200–560 µm) and *D. arbor* sp. nov. (148–396 µm); besides that, the shape of the rhabdome is also variable in *D. kellyae* sp. nov., where the tips of the rhabdomes can be blunt or sharp; (4) the size of the acanthomicroxeas in *D. kellyae* sp. nov. is larger (16.7–43.2–66.5 µm) compared to the other species, except when compared to *D. dissoluta* (41.6–68.0 µm; however, these values were taken from Pisera & Pomponi, 2015 where the authors presented a detailed description of the species, since in the original description, the species was poorly described and no measurements were given); (5) *D. kellyae* sp. nov., along with *D. arbor* sp. nov., are the only species with a wide acanthorhabds size range (5.3–13.3–24.9 µm and 6.7–16.1–25.9 µm, respectively) while the other species have a considerably narrower range (Table 2).

The species *D. inscripta* (Schmidt, 1879) was not included here for comparison because the type material was deciduous and the species is therefore considered *incertae sedis* (Pisera & Lévi, 2002d).

Family Macandrewiidae Schrammen, 1924

**Genus *Macandrewia*Gray, 1859**

**Diagnosis.** Macandrewiidae with phyllotriaenes/discotriaenes as ectosomal megascleres; choanosmal megascleres are oxeas and desmas with a triaenose crepsis; microscleres are microxeas (emended after Pisera & Lévi, 2002e).

**Definition.** Polymorphic Macandrewiidae; ectosomal spicules are dentate phyllotriaenes and/or discotriaenes; desmas are smooth with a triaenose (rarely monaxial) crepsis, and a terminal zygosis; oxeas are smooth; microscleres are microxeas (emended after Pisera & Lévi, 2002b).

**Type species.** *Macandrewia azorica* Gray, 1859 (type by monotypy).

***Macandrewia* cf. *azorica*Gray, 1859**

**Figure 18:**
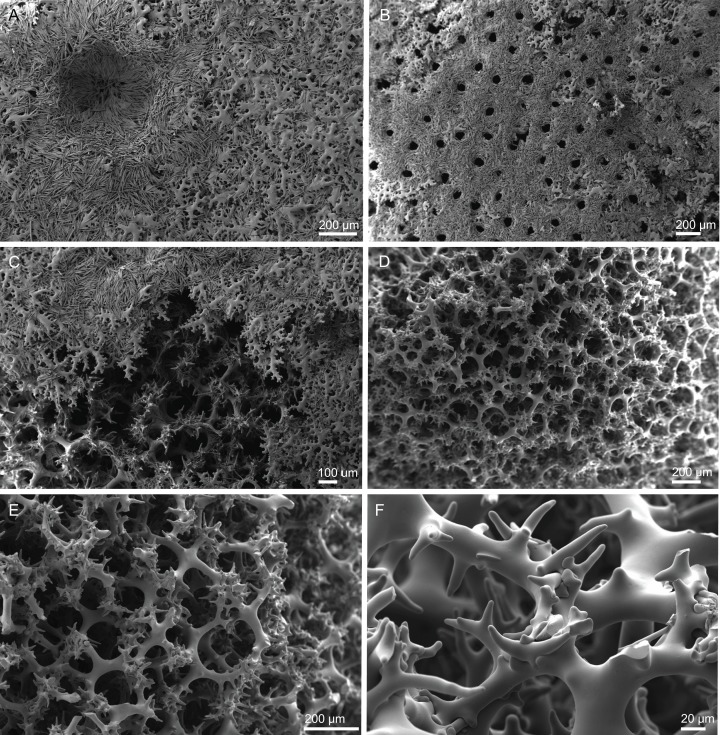
Surface and skeleton of *Macandrewia* cf. *azorica* Gray, 1859, specimen MNHN-IP-2008-220. (A) Upper/inner surface with large openings, (B) lower/outer surface with several small openings, (C) division between ectosome and choanosome: top of the image showing the ectosome formed by phyllotriaenes and microxeas, and the bottom showing the desmas, (D) choanosomal desmas, (E) choanosomal desmas resembling tetraclones, (F) detail of the sculpture of desmas and zygoses.

**Figure 19:**
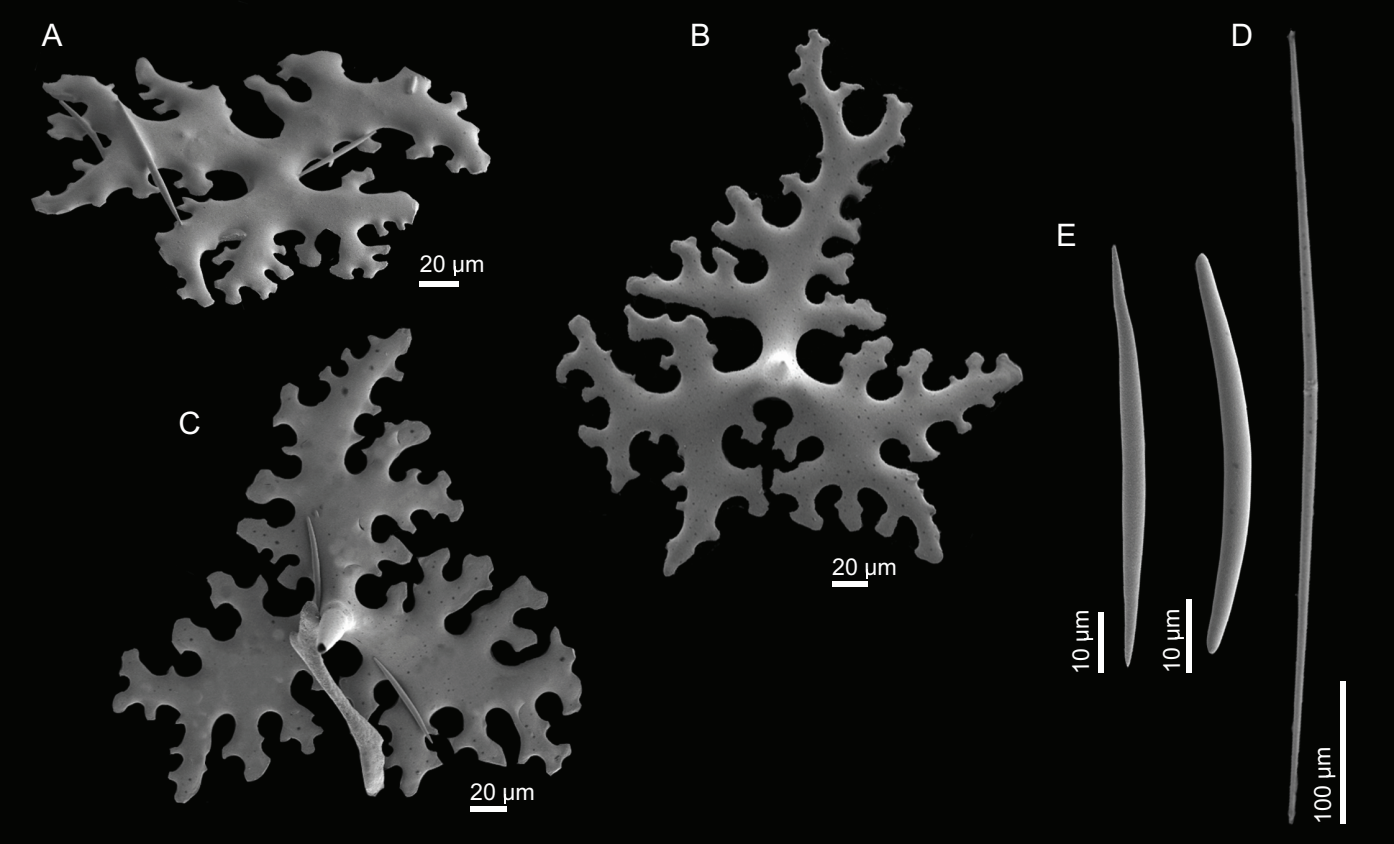
Spicules of *Macandrewia* cf. *azorica* Gray, 1859, specimen MNHN-IP-2008-220. (A)–(C) Phyllotriaenes with a very incised cladome, (D) oxeas, (E) microxeas.

Figures 2I, 18–19 and Table 3

**Material.** MNHN-IP-2008-217 (1993-02-03, Atlantis Seamount, beam trawl, DW263, 34°26′N, 30°32′W, 610 m), MNHN-IP-2008-220 (1993-02-03, Atlantis Seamount, epibenthic Warén dredge, DW258, 34°00′N, 30°12′W, 1,000 m), MNHN-IP-2008-225 (1993-02-06, Tyro Seamount, epibenthic Warén dredge, DW277, 34°00′N, 28°21′W, 1,000 m), MNHN-IP-2008-226 (1993-01, no data about station, 500 m), MNHN-IP-2008-229 (1993-01-06, Gran Canaria, epibenthic Warén dredge, DW129, 28°08′N, 15°52′W, 480 m), MNHN-IP-2008-249a (1993-01-06, Hyères Seamount, epibenthic Warén dredge, DW202, 31°16′N, 28°43′W, 640 m). All from *Seamount 2* campaign.

**Comparative material examined.** *M. azorica* (holotype BMNH 1851.7.28.16, S. Miguel island, Azores; HBOM 003:00784, Selvagens), *M. robusta* (MNHN-IP-2008-216, Hyères Seamount; MNHN-IP-2008-224, Hyères Seamount), *M. schusterae* sp. nov. (holotype MNHN-IP-2018-87 and paratype MNHN-IP-2018-90, Gorringe Seamount), *M. minima* sp. nov. (MNHN-IP-2008-222, Great Meteor Seamount).

**Description (MNHN-IP-2008-220).** Polymorphic sponges attached to the substrate by a thick pedicel/stem, 67 × 50 mm in size; lamellas are thin, rounded and undulate, 3–5 mm thick (Fig. 2I); inner surface (top) has openings visible to the naked eye, around 224 µm in size (Fig. 18A); outer surface is smooth with several little openings spread randomly through the entire sponge, 40–83 µm in size (Fig. 18B); colour is beige to light brown in ethanol.

**Skeleton.** Ectosome formed by a layer of overlapped phyllotriaenes covered by numerous microxeas (Figs. 18A–18C); small openings are surrounded by microxeas (Fig. 18A) whereas larger openings are delimited by both phyllotriaenes and microxeas (Fig. 18B); choanosomal skeleton formed by a regular and solid network of desmas with a triaenose crepsis, resembling tetraclone desmas (Figs. 18D and 18E), some oxeas and microxeas are spread in the interior of the sponge.

**Spicules (MNHN-IP-2008-220).**

1. **Desmas**, with a triaenose crepsis, compact, forming a regular mesh, resembling tetraclones; rays are smooth with branches, especially on the termination of the clone, measuring 212–281–343 × 16–34–51 µm in size; branches have blunt ends and their size is very variable, 34–54–74 × 5.9–8.3–11.5 µm in size (Figs. 18D–18F);
2. **Phyllotriaenes**, with particularly incised cladome with 194–267–333 µm in diameter, and a short conical-shaped rhabdome, 62–99–129 × 11.6–14.4–17.8 µm in size; cladomes are very variable, from a simple (Fig. 19A) to a very complex and incised shape (Figs. 19B and 19C);
3. **Oxeas**, smooth, slightly curved with pointed ends, 215–246–301 × 6.8–7.8–9.1 µm in size (Fig. 19D);
4. **Microxeas**, smooth, fusiform with blunt tips, slightly curved, very abundant, 33.3–55.0–83.6 × 2.5–3.9–5.1 µm in size (Fig. 19E).

**Distribution.** The specimens were found on the Atlantis Seamount between 420 and 610 m depth, and one specimen was collected in Gran Canaria at 480 m depth.

**Remarks**. Pisera & Lévi (2002d) re-described and illustrated the holotype of *M. azorica*, a specimen collected in the Azores archipelago. Since we also had access to the holotype of *M. azorica* we have made new measurements of the spicules, in order to fill the gaps of some spicule’s measurements missing in the redescription. The comparison of the holotype of *M. azorica* with the specimens collected during the campaigns *Seamount 1* and *2*, lead us to consider these specimens as *M*. cf. *azorica*. Although very similar in the habitus they differ from the holotype in two features: (1) desmas are considerably more robust and thicker, resembling tetraclones (MNHN-IP-2008-220: 16–34–51 µm width *vs* holotype BMNH 1851.7.28.16: 8.5–19.0–30.8 µm width), forming compact network, while in the redescription of the holotype, the desmas have a “variable morphology” resembling tetraclones or rhizoclones, with strongly branched clones at the tip, forming a complex and loose articulation (Pisera & Lévi, 2002e); (2) the size of the cladome of the phyllotriaenes (MNHN-IP-2008-220: 194–267–333 µm in diameter *vs* holotype BMNH 1851.7.28.16: 297–363–456 µm in diameter) and oxeas (MNHN-IP-2008-220: 215–246–301 µm length *vs* holotype BMNH 1851.7.28.16: 532–652–780 µm length) is considerably smaller (Table 3).

Nineteen large specimens were found in the same station in the Hyères seamount (station DW202), suggesting that the species may be forming a sponge ground in this area of the seamount.

***Macandrewia robusta*Topsent, 1904**

**Figure 20:**
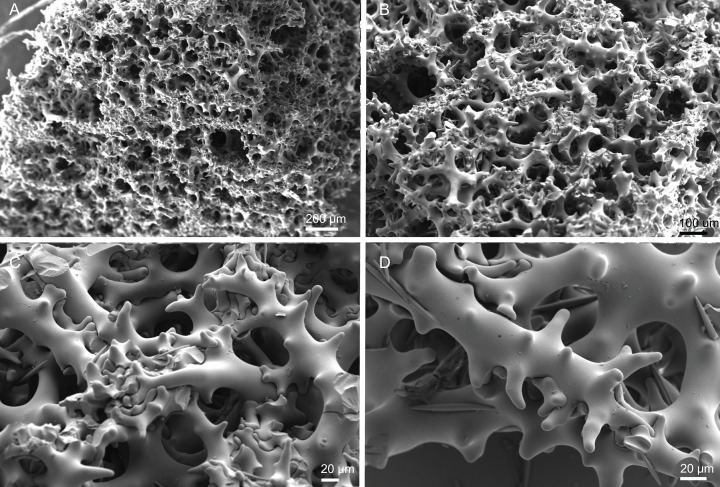
Skeleton of *Macandrewia robusta* Topsent, 1904, specimen MNHN-IP-2008-216. (A) Overview of choanosomal desmas, (B) desmas, (C) zygoses, (D) sculpture of the desmas.

**Figure 21:**
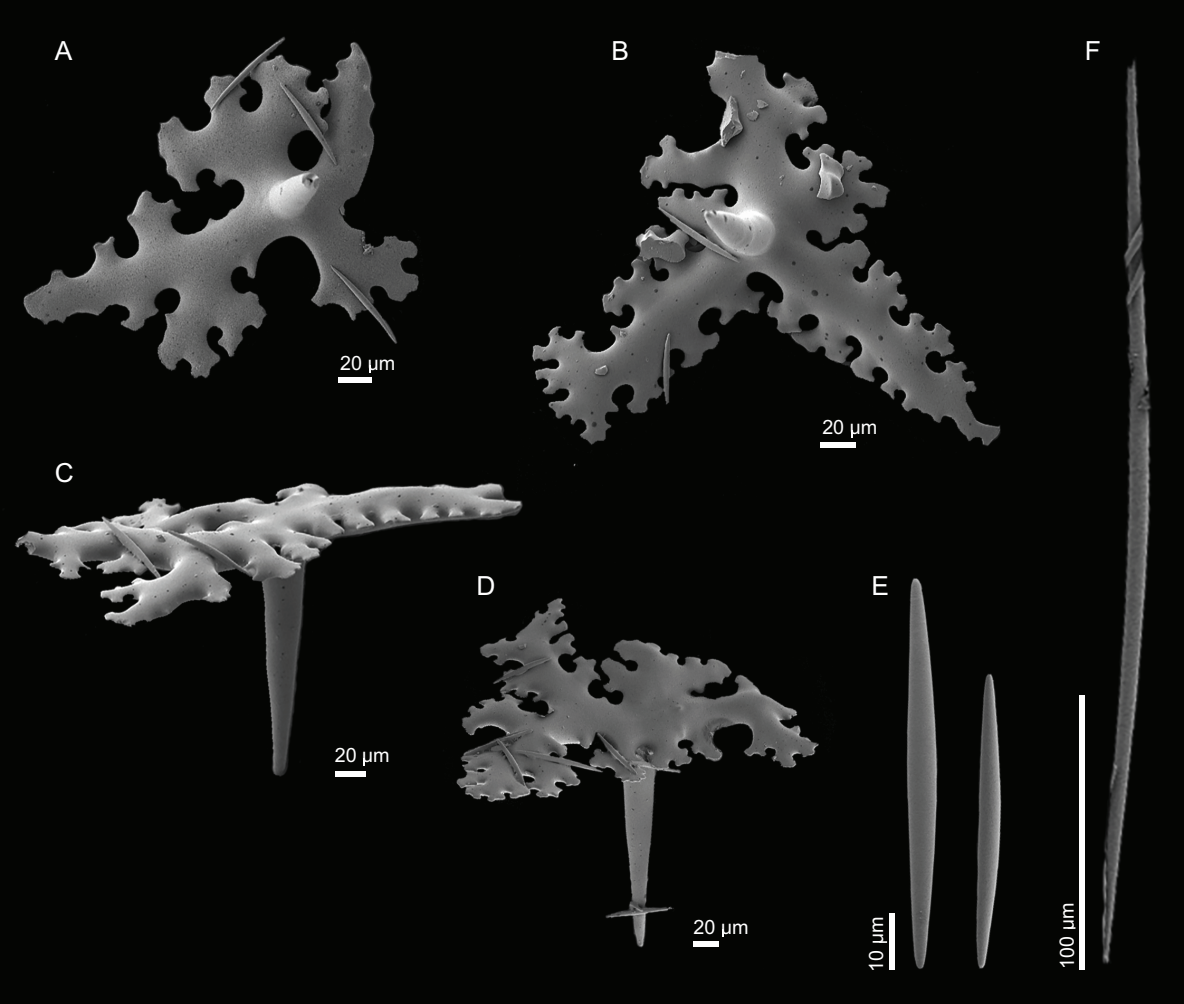
Spicules of *Macandrewia robusta* Topsent, 1904, specimen MNHN-IP-2008-216. (A)–(D) Phyllotriaenes, (E) microxeas, (F) oxea.

Figures 2J, 20–21 and Table 3

**Material.** MNHN-IP-2008-216, two specimens (1993-01-16, Hyères Seamount, epibenthic Warén dredge, DW184, 31°24′N, 28°52′W, 705 m), MNHN-IP-2008-224 two specimens (1993-01-16, Hyères Seamount, epibenthic Warén dredge, DW184, 31°24′N, 28°52′W, 705 m). All from *Seamount 2* campaign.

**Comparative material examined.** *M. azorica* (holotype BMNH 1851.7.28.16, S. Miguel island, Azores; HBOM 003:00784, Selvagens), *M*. cf. *azorica* (MNHN-IP-2008-217, Atlantis Seamount; MNHN-IP-2008-220, Atlantis Seamount; MNHN-IP-2008-225, Tyro Seamount; MNHN-IP-2008-226, no data; MNHN-IP-2008-229, Gran Canaria; MNHN-IP-2008-249a, Hyères Seamount), *M. schusterae* sp. nov. (holotype MNHN-IP-2018-87 and paratype MNHN-IP-2018-90, Gorringe Seamount), *M. minima* sp. nov. (MNHN-IP-2008-222, Great Meteor Seamount).

**Diagnosis.** Small ficiform to globular shape *Macandrewia* with a flattened top and a short and thick pedicel.

**Description (MNHN-IP-2008-216).** Small sponges with a ficiform to globular shape, 18–20 × 14–22 mm in size, attached to the substrate by a short and thick pedicel (8 mm in height and 16 mm width) (Fig. 2J); top of the sponge is flattened, smooth, where openings can be observed in small clusters leading to water canals giving a striated appearance to the sponge; openings and the subdermal water canals visible to the naked eye; lateral walls of the sponge are smooth with small openings spread evenly through this surface; in some individuals, the top or upper surface has a slight depression; colour varies from beige to light brown in alcohol.

**Skeleton.** Ectosome is composed of a layer of overlapped phyllotriaenes and numerous microxeas; these microxeas surround the openings radially; choanosomal skeleton formed by desmas, oxeas and dispersed microxeas; desmas form an irregular and very dense mesh (Fig. 20).

**Spicules (MNHN-IP-2008-216).**

1. **Desmas**, with a triaenose crepsis, compact, robust, with smooth clones that are very branched, 248–362 µm in size and 17–22–31 µm thick (Figs. 20A and 20B); clones have several short (18–41–75 µm), thick (7–10–12 µm) and blunt branches (Figs. 20D and 20E); the zygosis, that can be formed by numerous clones, is strong and complex (Fig. 20D).
2. **Phyllotriaenes**, very variable in shape, with a cladome particularly indented on the edges, 15–228–309 µm in diameter, with a conical rhabdome 46–91–141 × 13–19–25 µm in size (Figs. 21A–21D);
3. **Oxeas**, smooth with rounded tips, 203–329 × 7.2–8.2 µm thick (Fig. 21F).
4. **Microxeas**, smooth, with rounded extremities, slightly curved, 34.6–57.4–79.2 × 3.1–4.7–6.9 µm wide (Fig. 21E).

**Distribution.** These specimens were found on Hyères seamount at 705 m depth.

**Remarks.** In the specimens here examined, phyllotriaenes (165–230 µm *vs* 154–309; Table 3) and oxeas (330–400 *vs* 203–309; Table 3) are smaller when compared to previous records for the species (Topsent, 1904). However, *M. robusta* has a very distinct habitus in relation to the other *Macandrewia* described for the North Atlantic Ocean (Table 3). Its ficiform to globular shape, with a short and thick pedicel, contrasts with the cyathiform to flabellate shape with undulating rounded margins in *M. azorica*, the encrusting with standing trunks of *M. ramosa*, the foliate with thick lamellas in *M. schusterae* sp. nov., or the globular shape with a small pedicel as in *M. minima* sp. nov. (descriptions of the latter two below). Differences in spicule sizes were observed in another species analysed in this work as well as in other studies (see ‘Spicules dimensions’ section in the Discussion for further information regarding this topic).

Two specimens from the *Seamount 2* collection could not be confidently identified down to species level (MNHN-IP-2008-228 and MNHN-IP-2018-94). They are very small fragments, seemingly encrusting, and most likely it is a *Macandrewia* at an early stage of development. The spicules were measured and they fall within the size range found in *M. robusta*.

***Macandrewia schusterae* sp. nov.**

**Figure 22:**
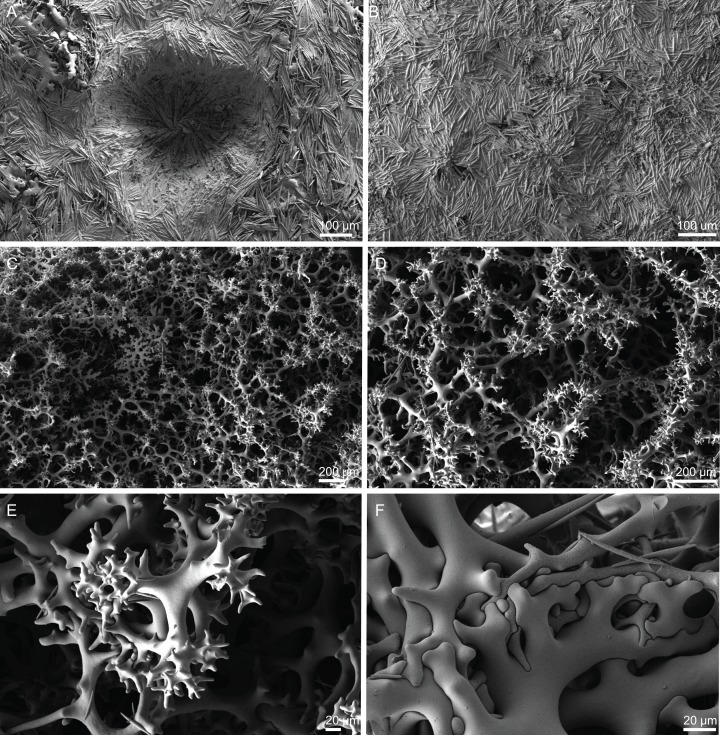
Surface and skeleton of *Macandrewia schusterae* sp. nov., holotype MNHN-IP-2018-87. (A) Internal surface with large openings, (B) exterior surface with small openings, (C) overview of choanosomal skeleton, (D) choanosomal desmas, (E) detail of the sculpture of desmas, (F) zygoses.

**Figure 23:**
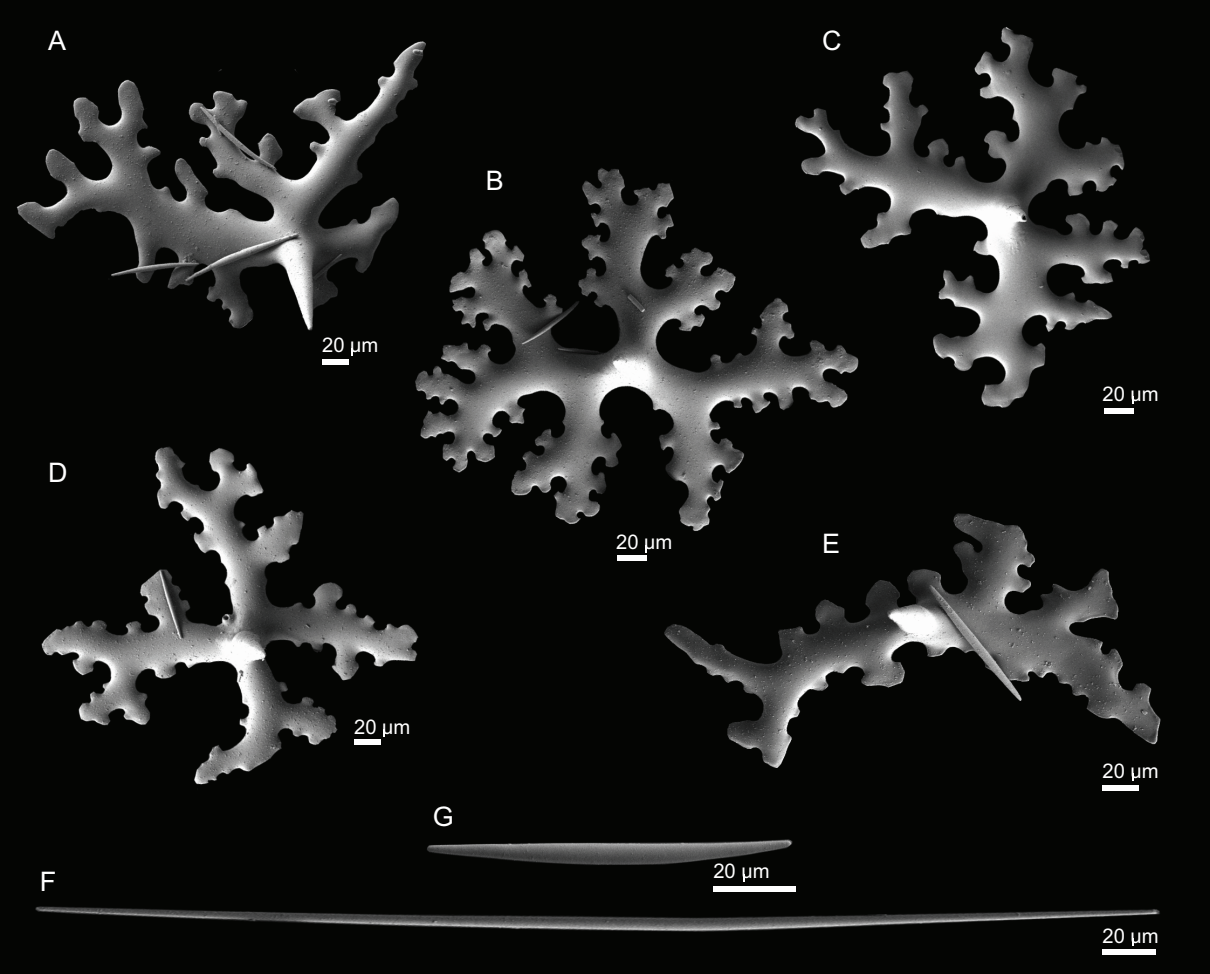
Spicules of *Macandrewia schusterae* sp. nov., holotype MNHN-IP-2018-87. (A)–(E) Phyllotriaenes, (F) oxea, (G) microxea.

Figures 2K, 22–23 and Table 3

urn:lsid:zoobank.org:act:2BA2C1EF-8FAB-4C91-89CB-DCB59DDA61EB

**Holotype**. MNHN-IP-2018-87 (1988-09-26, Gorringe Seamount, beam trawl, CP28, 36°28′N, 11°29’W, 605–675 m, *Seamount 1 campaign*).

**Paratype**. MNHN-IP-2018-88 (1988-09-26, Gorringe Seamount, beam trawl, CP28, 36°28’N, 11°29′W, 605–675 m, *Seamount 1* campaign).

**Other material.** MNHN-IP-2018-90, six specimens (1988-09-26, Gorringe Seamount, beam trawl, CP28, 36°28′N, 11°29′W, 605–675 m, *Seamount 1* campaign), MNHN-IP-2018-89 (1988-09-26, Gorringe Seamount, beam trawl, CP28, 36°28′N, 11°29′W, 605–675 m, *Seamount 1* campaign), MNHN-IP-2018-91 (1988-09-26, Gorringe Seamount, beam trawl, CP28, 36°28′N, 11°29′W, 605–675 m, *Seamount 1* campaign), MNHN-IP-2008-219 (1993-02-06, Tyro Seamount, epibenthic Warén dredge, DW279, 33°56′N, 28°24′W, 805 m, *Seamount 2* campaign), MNHN-IP-2008-230 (1993-02-01, Plato Seamount, epibenthic Warén dredge, DW246, 33°14′N, 29°36′W, 520 m, *Seamount 2* campaign).

**Comparative material examined.** *M. azorica* (holotype BMNH 1851.7.28.16, S. Miguel island, Azores; HBOM 003:00784, Selvagens), *M*. cf. *azorica* (MNHN-IP-2008-217, Atlantis Seamount; MNHN-IP-2008-220, Atlantis Seamount; MNHN-IP-2008-225, Tyro Seamount; MNHN-IP-2008-226, no data; MNHN-IP-2008-229, Gran Canaria; MNHN-IP-2008-249a, Hyères Seamount), *M. robusta* (MNHN-IP-2008-216, Hyéres Seamount; MNHN-IP-2008-224, Hyères Seamount), *M. minima* sp. nov. MNHN-IP-2008-222 (Great Meteor Seamount).

**Diagnosis.** Foliate to vase shaped *Macandrewia* with thick, irregular and undulated lamellas, with a small pedicel.

**Description (holotype MNHN-IP-2018-87).** Massive, foliate to vase shape with undulate lamellas, 94 mm high and 142 mm wide at the top and 45 mm wide at the base, usually attached to the substrate by a large pedicel; lamellas are generally irregular and contorted; walls are thick, 7–10 mm (Fig. 2K); interior surface with openings slightly elevated and evenly distributed, 278–378 µm in diameter (Fig. 22A); subdermal water canals are visible on the inner surface; external surface is smooth and covered by small openings, 29–98 µm in size (Fig. 22B); colour light brown to white in alcohol.

**Skeleton.** Ectosome has phyllotriaenes that are covered by numerous microxeas, surrounding the openings radially (Figs. 22A and 22B); choanosome has desmas, oxeas and dispersed microxeas; desmas are compact, irregular and create a dense network (Figs. 22C and 22D).

**Spicules (holotype MNHN-IP-2018-87).**

1. **Desmas**, with a triaenose crepsis, smooth, irregular, forming an intricate and complex net, 301–386–463 × 10.2–19.9–39.2 µm in size (Figs. 22C–22E); clones have the terminations splitting in several branches that are usually short, and blunt, 17–37–78 × 5–9–15 µm in size; zygoses is complex and solid (Fig. 22F).
2. **Phyllotriaenes**, cladome it is particularly incised on the edges, 177–304–420 µm in diameter; short rhabdome 67–119–178 × 13–21–26 µm in size (Figs. 23A–23E).
3. **Oxeas**, smooth with rounded tips, 263–437–620 × 8.1–12.4–16.0 µm in size (Fig. 23F).
4. **Microxeas**, smooth, with round edges, 43.8–67.9–95.2 × 2.5–4.3–7.7 µm size (Fig. 23G).

**Etymology.** Named after Dr. Astrid Schuster for her contributions in the field of molecular paleobiology of lithistid demosponges.

**Distribution.** This specimen was found on Gorringe, Tyro and Plato Seamounts between 520 and 805 m depth.

**Remarks.** *M. schusterae* sp. nov. is here proposed as a new species due to its particular habit, the sculpture of the desmas and size of the spicules. *M. schusterae* sp. nov. has a foliate shape with contorted lamellas, sometimes resembling a *Leiodermatium* sp., that contrasts with the ficiform to globular shape with a flattened top in *M. robusta*, the flabellate to undulate masses with thin lamellas in *M. azorica*, the ramose shape in *M. ramosa* Topsent, 1904 and the small ball shape in *M. minima* sp. nov. The desmas have a different sculpture compared to the other *Macandrewia* species, as the zygomes have extremely ramified long and thin branches, forming a very strong zygosis (Figs. 22C–22E). This new species also presents a relatively wide range of spicule sizes, mainly on phyllotrianes (cladome: 177–304–420 µm; rhabdome: 67–119–178 µm), oxeas (263–437–620 µm) and microxeas (43.8–67.9–95.2 µm), a feature that is not so common on the other species (Table 3).

***Macandrewia minima* sp. nov.**

**Figure 24:**
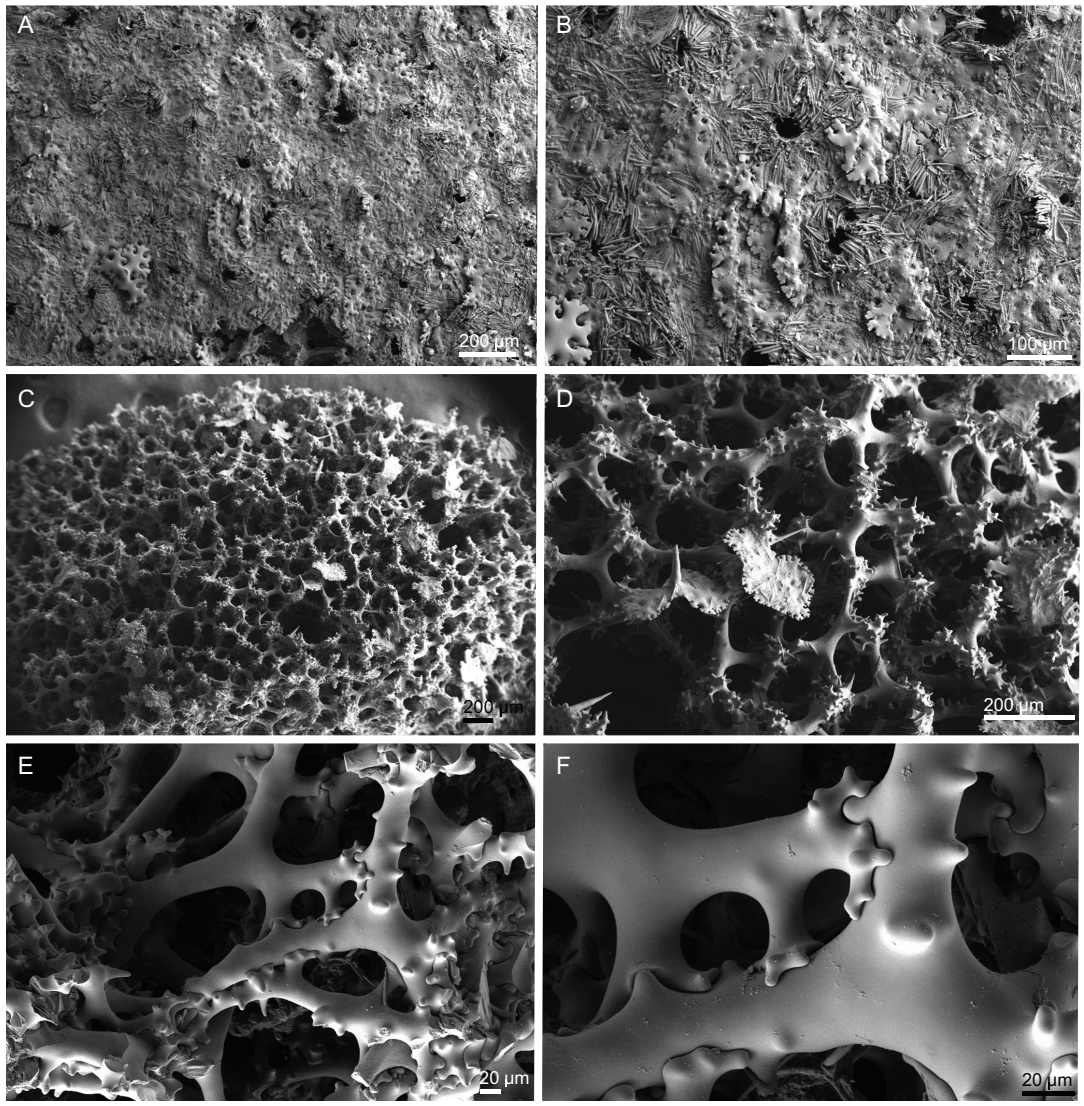
Surface and skeleton of *Macandrewia minima* sp. nov., holotype MNHN-IP-2008-222. (A) Surface, (B) close-up of the surface showing the microxeas surrounding the pores radially, (C) overview of desmas, (D) choanosomal desmas, (E) sculpture of desmas, (F) zygosis.

**Figure 25:**
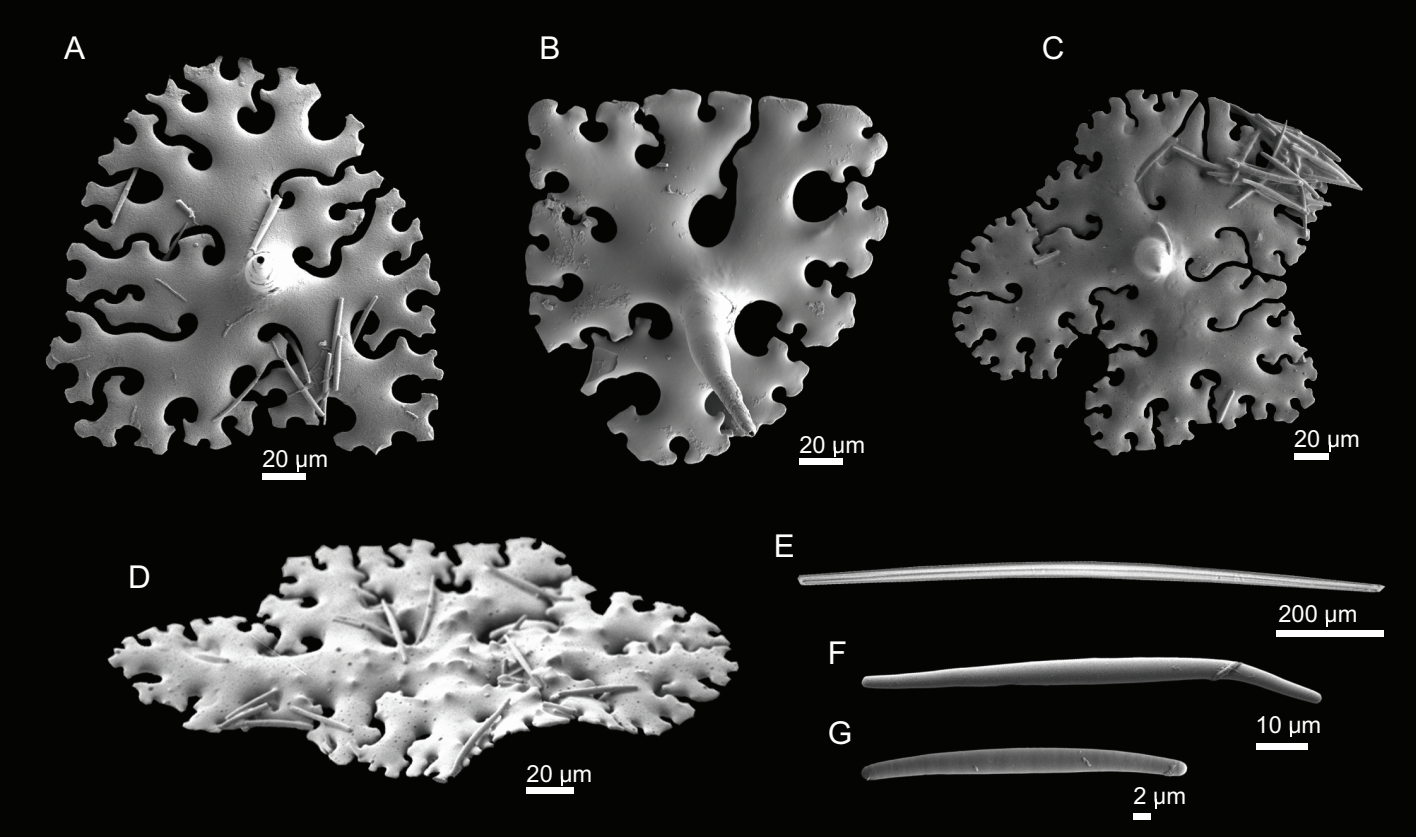
Spicules of *Macandrewia minima* sp. nov., holotype MNHN-IP-2008-222. (A)–(C) Bottom view of the cladomes of the phyllotriaenes, (D) top view of cladome showing the small protuberances, (E) oxea-tips are broken, (F) and (G) microxeas.

Figures 2L, 24–25 and Table 3

urn:lsid:zoobank.org:act:E405AE49-5636-4778-9B07-ED39E9EBB7BE

**Holotype**. MNHN-IP-2008-222 (1993-01-11, Great Meteor Seamount, epibenthic Warén dredge, DW148, 30°12′N, 28°25′W, 615 m, *Seamount 2* campaign).

**Comparative material.** *M. azorica* (holotype BMNH 1851.7.28.16, S. Miguel island, Azores; HBOM 003:00784, Selvagens), *M*. cf. *azorica* (MNHN-IP-2008-217, Atlantis Seamount; MNHN-IP-2008-220, Atlantis Seamount; MNHN-IP-2008-225, Tyro Seamount; MNHN-IP-2008-226, no data; MNHN-IP-2008-229, Gran Canaria; MNHN-IP-2008-249a, Hyères Seamount), *M. robusta* (MNHN-IP-2008-216, Hyéres Seamount; MNHN-IP-2008-224, Hyères Seamount), *M. schusterae* sp. nov. (holotype MNHN-IP-2018-87 and paratype MNHN-IP-2018-90, Gorringe Seamount).

**Diagnosis.** Small ball shaped *Macandrewia* with tuberculated phyllotriaenes.

**Description (holotype MNHN-IP-2008-222).** Small sponge of round-globular shape, 15–20 × 16–17 mm in size, with a very shorth and slender pedicel; surface is smooth with visible openings scattered on the top, 34–69 µm in diameter; colour beige to white in alcohol (Fig. 2L).

**Skeleton.** Ectosome has a layer of phyllotriaenes covered by large amounts of microxeas; microxeas surround the openings radially (Figs. 24A and 24B); choanosome has desmas, with a triaenose crepsis, forming a compact and irregular network (Fig. 24C); oxeas and microxeas are spread through the choanosome but in small amounts compared to the ectosome.

**Spicules (holotype MNHN-IP-2008-222).**

1. **Desmas**, with a triaenose crepsis, robust, usually smooth in the centre, but some branches/rugosities can also be observed, 268–318–348 µm in length and 7–29–50 µm thick (Figs. 24C–24E); clones extremities split in several small branches, 17–37–78 × 5–9–15 in size; zygosis is complex and strong giving a bulb appearance to this part of the desma (Figs. 24D–24F).
2. **Phyllotriaenes**, cladome generally more compact, with incised clades that are ornamented by tubercles, 136–222–284 µm in diameter; short rhabdome with a conical shape, 58–99–136 × 14–19–25 µm in size (Figs. 25A–25D).
3. **Oxeas**, slightly curved, 197–251–316 × 7.5–11.9–16.2 µm in size (Fig. 25E).
4. **Microxeas**, often curved, tips are blunt, 25.9–48.3–74.2 × 3.1–4.4–7.0 µm in size (Figs. 25F and 25G).

**Etymology.** From the Latin *minima* = small.

**Distribution.** Only known from its type locality, the Great Meteor Seamount at 615 m depth.

**Remarks.** *M. minima* sp. nov. differs from the other *Macandrewia* in the considerably smaller size of its spicules (see Table 3), its globular shape and in the characteristic tubercles of the phyllotriaenes (only observed in this species).

**Family Phymaraphiniidae Schrammen, 1924**

**Genus *Exsuperantia*Özdikmen, 2009**

**Synonymy.** *Rimella* Schmidt, 1879: 21 (preoccupied); *Racodiscula* sensu Topsent, 1928, 1904, 1892 (wrong genus identification).

**Diagnosis.** Clavate to columnar Phymaraphiniidae with phyllotriaenes as ectosomal spicules (Pisera & Lévi, 2002f).

**Definiton.** Clavate to columnar, globular knob-like small sponges. Desmas are triders with smooth and/or tuberculated tubercles. Other spicules are smooth phyllo- to discotriaenes and subtylostyles to tylotes as megascleres, and acanthomicroxeas, acanthorhabds and streptasters as microscleres (emended after Carvalho & Pisera, 2019; Pisera & Lévi, 2002e).

*Type species. Exsuperantia clava* (Schmidt, 1879) (type by monotypy).

***Exsuperantia archipelagus*** Carvalho & Pisera, 2019

**Figure 26:**
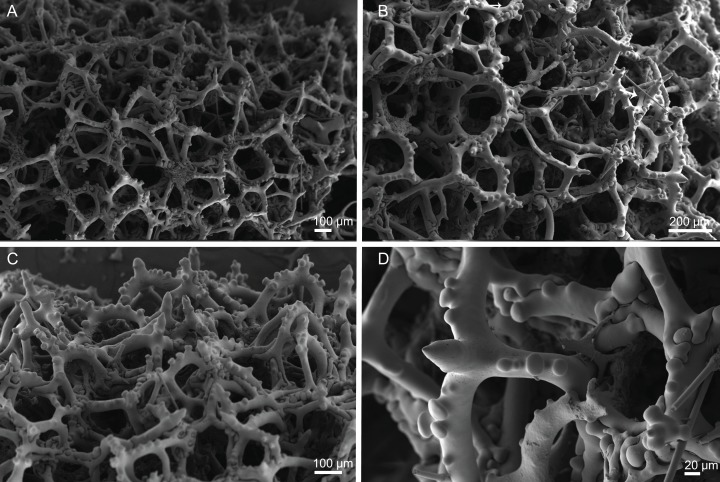
Skeleton of *Exsuperantia archipelagus* Carvalho & Pisera, 2019, specimen MNHN-IP-2008-196. (A) Overview of choanosomal triders, (B) subtylostyles crossing the skeleton, (C) detail of trider-type desmas, (D) zygosis and close up of a trider showing the desma ornamentation.

**Figure 27:**
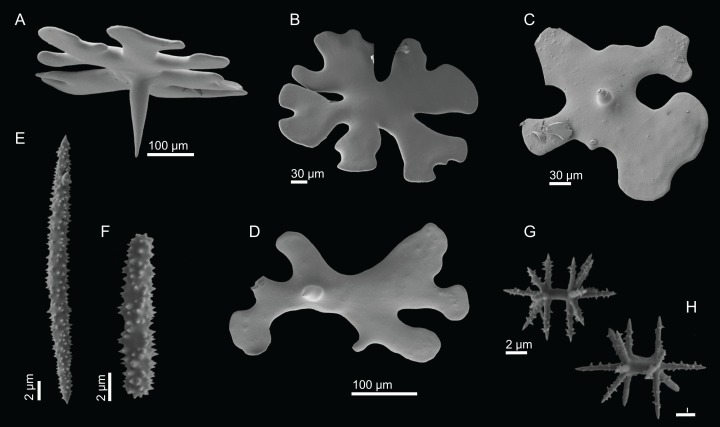
Spicules of *Exsuperantia archipelagus* Carvalho & Pisera, 2019, specimen MNHN-IP-2008-196. (A)–(D) Phyllotriaenes, (E) acanthomicroxeas, (F) acanthorhabds, (G) and (H) streptasters/amphiasters.

Figures 3A, 26–27 and Table 4

**Table 4:** Comparative table of external morphology and spicular micrometries of all *Exsuperantia* species recorded in the North Atlantic Ocean. Spicule measurements (*n* = 30 unless stated otherwise) are presented as minimum–mean–maximum. Data compiled from the original descriptions, or subsequent re-descriptions of type material (marked with numbers).

Species	Habitus	Size	Triders	Phylo- to discotriaenes	Subtylostyles to tylotes	Acanthomicroxeas	Acanthorhabds	Amphiasters	Locality
^1^*E. clava* (Schmidt, 1879) (Syntype MZUS PO146)	Cylindrical to clavate	Up to 30 mm long, 10 mm thick	230–320 µm in size	–	Present	Fusiform, occasionally centrotylotes	Present	Slender rays	Cuba (depth unknown)
^2^*E. archipelagus* Carvalho & Pisera, 2019 (Holotype MNHN DT-782/1)	Columnar to ficiform, with or without lateral protuberances or branches; surface is smooth, with marked water canals; colour beige to whitish	20–30 mm × 10–20 mm	409–693 × 52–98 µm in size	Phyllotriaenes. Cladome: 640–890 µm in diameter; rhabdome: 229–320 × 71 µm	Subtylostyles to tylostyles: 260–1114 × 3–38 µm in size	31–47 × 2.9–4.2 µm	18–24 × 2.3–4.1 µm	15–19 × 1.2–1.7 µm	Azores (168–594 m depth)
*E. archipelagus* (MNHN-IP-2008-196).	Columnar to ficiform in habitus, with or without lateral protuberance; water canals visible on the surface; colour beige	22–23 × 8–18 mm	260–362–464 (*n* = 7) × 15–29–44 µm thick (*n* = 15)	Phyllotriaenes. Cladome: 199–358–470 µm in diameter (=11); rhabdome: 140 × 34.4 µm (*n* = 1)	296–515–618 × 6.1–9.7–13.4 µm (*n* = 11)	18.6–25.0–44.0 × 1.4–2.2–3.7 µm	7.2–12.0–15.9 × 1.2–1.8–3.2 µm	5.3–8.6–15.0 µm (*n* = 15)	Hyères Seamount (310 m depth)
*E. levii* sp. nov. (Holotype MNHN-IP-2008-201)	Clusters of globular to ficiform knob-like short fingers		293–346–503 µm in size, 28–45–67 µm thick (*n* = 12)	Phyllo- to discotriaenes. Cladome: 143–299–486 diameter (*n* = 20); rhabdome: 25–73–130 × 10 –28–44 µm (*n* = 10)	234–307–436 µm × 8.6–9.8–11.3 µm (*n* = 6)	21.6–28.2–35.6 × 1.7–2.6–3.8 µm	10.3–14.1–19.3 × 1.9–2.7–3.5 µm (*n* = 25)	7.4–10.0–14.8 µm (*n* = 20).	Hyères Seamount (480 m depth)

**Notes:**

1 Pisera & Lévi (2002d).

2 Carvalho & Pisera (2019).

‘–’ no information/not mentioned.

**Synonymy.** *Racodiscula* sensu Topsent, 1892 (Topsent, 1904, 1928) (wrong generic assignment); *Exsuperantia* sp. Carvalho, Pomponi & Xavier (2015).

**Material.** MNHN-IP-2008-191 (1993-02-06, Tyro Seamount, epibenthic Warén dredge, st. DW277, 34°00′N, 28°21′W, 1,000 m), MNHN-IP-2008-192 (1993-02-03, Atlantis Seamount, epibenthic Warén dredge, st. DW265, 34°29′N, 30°36′W, 545 m), MNHN-IP-2008-195 (1993-02-02, Atlantis Seamount, beam trawl, st. CP257, 34°04′N, 30°15′W, 338 m), MNHN-IP-2008-196 (1993-01-17, Hyères Seamount, epibenthic Warén dredge, st. DW188, 31°30′N, 29°00′W, 310 m), MNHN-IP-2008-199 (1993-02-02, Atlantis Seamount, epibenthic Warén dredge, st. DW258, 34°00′N, 30°12′W, 420 m), MNHN-IP-2008-200 (1993-01-31, Plato Seamount, epibenthic Warén dredge, st. DW242, 33°12′N, 28°57′W, 710 m), MNHN-IP-2008-202 (1993-02-02, Atlantis Seamount, epibenthic Warén dredge, st. DW254, 34°05′N, 30°13′W, 480 m), MNHN-IP-2008-240 (1993-02-01, Plato Seamount, epibenthic Warén dredge, st. DW246, 33°14′N, 29°36′W, 520 m), MNHN-IP-2008-243 (1993-02-02, Atlantis Seamount, epibenthic Warén dredge, st. DW258, 34°00′N, 30°12′W, 420 m). All from the *Seamount 2* campaign.

**Comparative material examined.** *E. archipelagus* (holotype MNHN DT 782/1 Azores; paratype MNHN DT 782/2, Azores; paratype DOP 1976, Azores); *E. levii* sp. nov. (holotype MNHN-IP-2008-201, Hyères Seamount).

**Diagnosis.** Columnar to ficiform *Exsuperantia* with trider-type desmas that have smooth tubercles (few presenting rugosities).

**Description (MNHN-IP-2008-196).** Small phymarapiniid 22–23 × 8–18 mm in size, columnar to ficiform in habitus, with or without lateral protuberances (Fig. 3A); some specimens have a “V” shape morphology; surface is smooth with conspicuous subdermal water canals giving a striped appearance to the sponge; oscula or pores are not visible; colour beige in ethanol.

**Skeleton.** Ectosome is formed by a layer of phyllotriaenes covered by large amounts of microscleres: openings are surrounded by these microscleres; choanosomal skeleton is mainly built of trider-type desmas, that form a regular network with large spaces in between (Fig. 26); some subtylostyles (Fig. 26B) and microscleres are also present and spread through the skeleton.

**Spicules (MNHN-IP-2008-196).**

1. **Trider-type desmas**, smooth, very tuberculated, 261–342–419 × 23–30–44 µm in size (Figs. 26A–26C); tubercles are smooth, sometimes with rugosities, 7–10–11 µm diameter; tip of the trider is smooth and has a conical shape (Fig. 26D).
2. **Phyllotriaenes**, irregular, smooth cladome 412–450–493 µm in diameter (Figs. 27A–27D), long rhabdome, 43–75–126 µm in size, with pointed tip (Fig. 27A).
3. **Subtylostyles to tylotes**, smooth, 401–542–629 × 6.0–9.9–12.8 µm in size (Fig. 26B).
4. **Acanthomicroxeas**, slender, with sharp tips, 16.8–22.1–28.1 × 1.6–2.2–3.1 µm (Fig. 27E).
5. **Acanthorhabds**, thick with blunt ends, 9.8–12.7–17.5 × 1.2–2.0–2.8 µm (Fig. 27F).
6. **Amphiasters**, with several arms covered by spines, 5.0–6.6–8.6 µm long (Figs. 27G and 27H).

**Distribution.** *E. archipelagus* was found in Tyro, Hyères, Atlantis, and Plato Seamounts between 280 and 1,000 m depth and also in Gran Canaria island at 660 m depth.

**Remarks.** The size of the spicules measured in these specimens are considerable smaller when compared to the type material (Carvalho & Pisera, 2019) (Table 4), but see Discussion for more information regarding this topic.

***Exsuperantia levii* sp. nov.**

**Figure 28:**
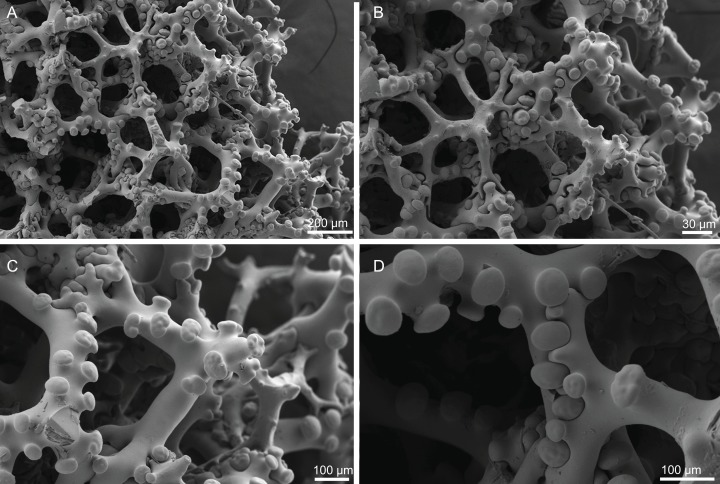
Skeleton of *Exsuperantia levii* sp. nov., specimen MNHN-IP-2008-201. (A) Outline of trider-type desmas, (B) triders, (C) zygosis, (D) detail of a trider desma.

**Figure 29:**
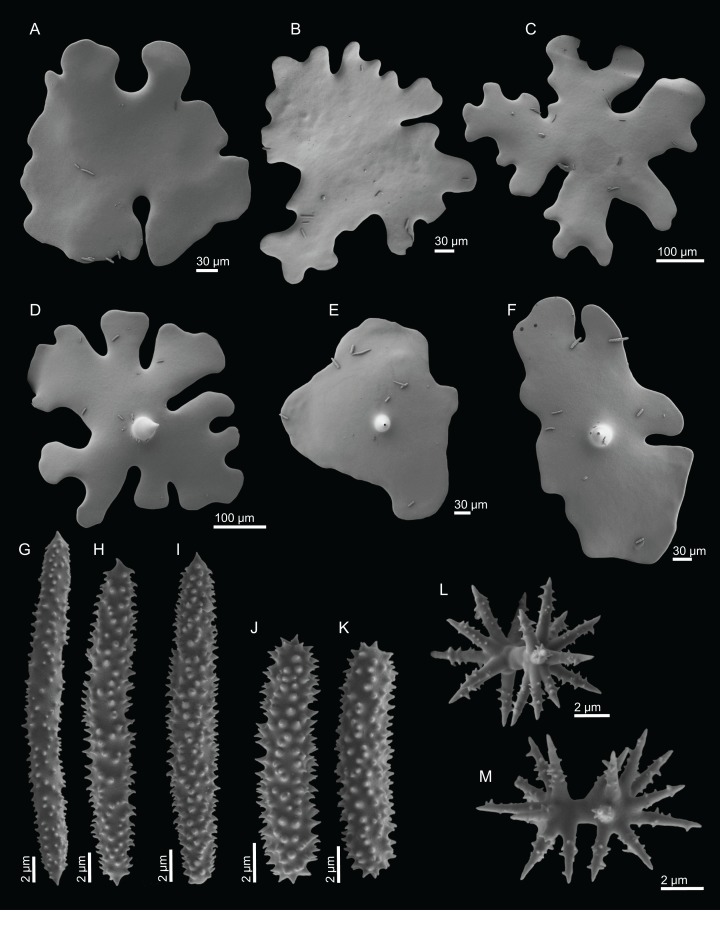
Spicules of *Exsuperantia levii* sp. nov., specimen IP-2008-201. (A)–(C) Top view of cladomes of phyllo- to discotriaenes, (D) and (E) bottom view of cladomes, (G)–(I) acanthomicroxeas, (J) and (K) acanthorhabds, (L) and (M) streptasters/amphiasters.

Figures 3B, 28–29 and Table 4

urn:lsid:zoobank.org:act:24B5934A-4767-4429-B172-A649C4CE0D83

**Holotype.** MNHN-IP-2008-201 (1993-01-16, Hyères Seamount, epibenthic Warén dredge, st. DW182, 31°23′N, 28°54′W, 480 m, *Seamount 2* campaign).

**Comparative material examined.** *E. archipelagus* (holotype MNHN DT 782/1 Azores; paratype MNHN DT 782/2, Azores; paratype DOP 1976, Azores).

**Diagnosis.** Clusters of globular to ficiform knob-like short fingers with apical osculum; phyllo- to discotriaenes as ectosomal megascleres.

**Description (holotype MNHN-IP-2008-201).** Clusters of globular to ficiform knob-like short fingers, 30 mm in length and 29 mm wide; oscula, approximately 2 mm in diameter, are located on the top of the knobs (Fig. 3B); surface is rugose with a striated appearance due to the visible subdermal water canals; colour is brown in ethanol.

**Skeleton.** Ectosome is composed by phyllo- to discotriaenes that are very variable in shape, and several microscleres; choanosomal skeleton has regular and articulated triders, forming an irregular and relatively loose network (Fig. 28); subtylostyles are present crossing the skeleton (Figs. 28A and 28B); microscleres are present and very abundant, except for streptasters that are less numerous.

**Spicules (holotype MNHN-IP-2008-201).**

1. **Trider-type desmas**, regular, smooth, 293–346–503 × 28–45–67 µm in size, with large and flattened tubercles that can be smooth or very tuberculated, 15.4–21.2–29.9 µm in diameter (Figs. 28A–28D).
2. **Phyllo- to discotriaenes**, smooth, cladome very variable in shape, 143–299–486 µm in diameter; rhabdome has a conical shape and a sharp tip, 25–73–130 µm × 10–28–44 µm in size (Figs. 29A–29F).
3. **Subtylostyles**, smooth, large, slightly curved, 234–307–436 × 8.6–9.8–11.3 µm in size (Figs. 28A and 28B).
4. **Acanthomicroxeas**, thin, slightly curved, with sharp tips, 21.5–26.2–31.6 × 1.8–2.9–4.1 µm (Figs. 29G and 29H); occasionally, these spicules are irregular, and exhibit one sharp and one blunt tip, resembling an intermediate stage between an acanthomicroxea and an acanthorhabd (Fig. 29I);
5. **Acanthorhabds**, small, robust, 9.3–15.1–22.5 × 1.6–2.8–3.8 µm in size (Figs. 29J and 29K).
6. **Amphiasters**, thin with spiny arms, 5.9–8.2–11.5 µm long (Figs. 29L and 29M).

**Distribution.** *Exsuperantia levii* sp. nov. is known from its type locality, the Hyères Seamount at 480 m depth.

**Etymology.** Named after Professor Claude Lévi from the Muséum National d’Histoire Naturelle Paris (MNHN) for his lifelong contribution to the taxonomy and systematics of Porifera, including lithistid sponges.

**Remarks.** Recently, a revision of the genus *Exsuperantia* allowed to clarify some taxonomic problems by establishing two species, *E. clava* (NWA) and *E. archipelagus* (NEA), that were previously considered a single species (Carvalho & Pisera, 2019). According to the authors, the main differences between these two species are the desmas morphology and ornamentation.

Here we propose *E. levii* sp. nov. as a new species, third of the genus, based not only on desmas morphology and ornamentation, but also on the habitus of this new species. The trider-type desmas on *E. levii* sp. nov. resemble the ones found in *E. clava*, i.e., the tubercles are ornamented and the tip of the trider has a tubercle, while in *E. archipelagus* it usually has a conical shape. In general, the size of the spicules of *E. levii* sp. nov. is smaller when compared to the holotype *E. archipelagus* (unfortunately the size of spicules of the *E. clava* is not known, with exception of the desmas, since the type material was deciduous and microscleres were not present (Pisera & Lévi, 2002f)), however, the most distinct feature is the habitus of *E. levii* sp. nov.: a cluster of globular knob-like fingers with large apical oscula on top, contrasting with the columnar to ficiform morphology of the other two species.

**Suborder Spirophorina Bergquist & Hogg, 1969**

**Family Azoricidae Sollas, 1888**

**Genus *Leiodermatium*Schmidt, 1870**

**Diagnosis.** Azoricidae with spiny rhizoclones and diactines as megascleres; ectosomal spicules and microscleres are absent (Pisera & Lévi, 2002i).

**Definition.** Lamellate, plate-like, foliose, vase- or ear-shaped Azoricidae; oscules are visible; choanosomal desmas are spiny rhizoclones; megascleres are diactines; microscleres are absent (Kelly, 2007; modified from Pisera & Lévi, 2002h).

**Type species.** *Leiodermatium lynceus* Schmidt, 1870.

***Leiodermatium lynceus*Schmidt, 1870**

**Figure 30:**
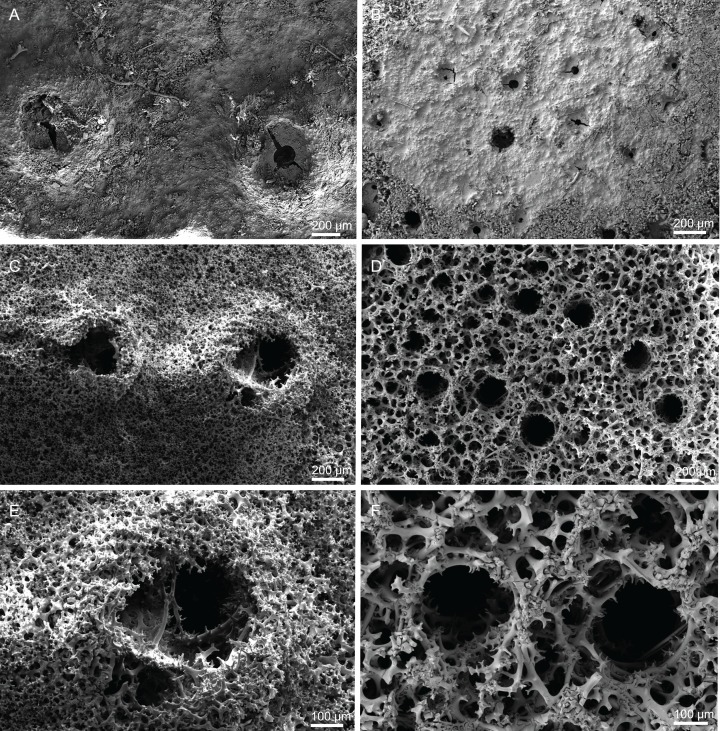
Surface and skeleton of *Leiodermatium lynceus* Schmidt, 1870, specimen MNHN-IP-2018-93. (A) Overview of the outer surface with larger and elevated oscula (surface not digested in nitric acid), (B) overview of the inner surface with smaller and depressed pores (surface not digested), (C) overview of the outer surface with larger and elevated pores (digested surface in nitric acid), (D) overview of the inner surface with smaller pores (digested surface), (E) detail of the oscula, (F) detail of the pores.

**Figure 31:**
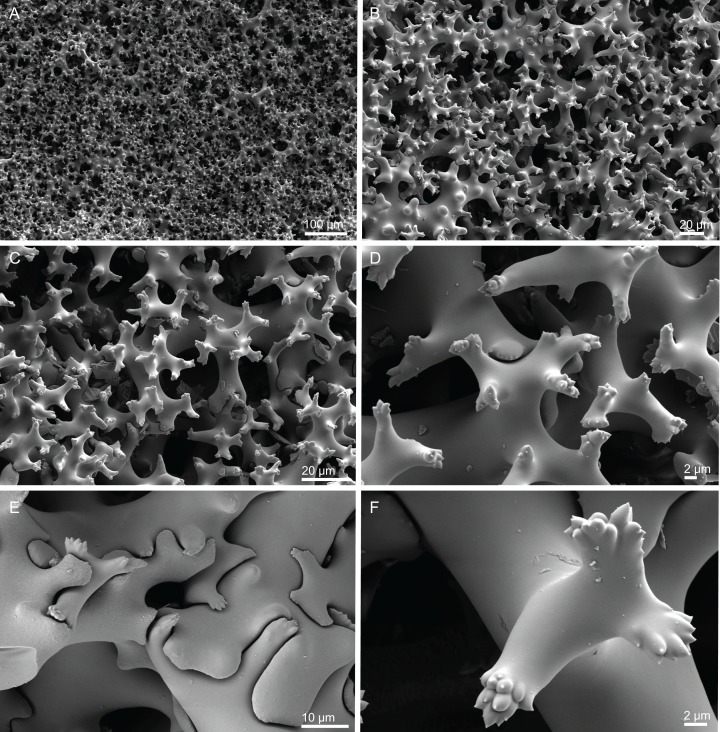
Skeleton of *Leiodermatium lynceus* Schmidt, 1870, specimen MNHN-IP-2018-93. (A) Overview of choanosomal desmas, (B) rhizoclone desmas forming a very compact mesh, (C) detail of rhizoclones, (D) ornamentation of rhizoclone desmas, (E) zygosis between several rays, (F) detail of the sculpture of the desma.

Figures 3C, 30–31 and Table 5

**Table 5:** Comparative table of external morphology and spicular micrometries of all *Leiodermatium* species recorded in the North Atlantic and Mediterranean Sea. Spicule measurements (*n* = 30 unless stated otherwise) are presented as minimum–mean–maximum. Data compiled from the original descriptions, or subsequent re-descriptions of type material (marked with numbers).

	Habitus	Size	Rhizoclones	Oxeas	Locality
^1^*Leiodermatium lynceus* Schmidt, 1870 (Holotype MZUS PO145)	Foliate or vase to ear-shape; outer surface with large and elevated oscules (500–750 µm) and inner surface with small pores (156–188 µm)	60 × 30 mm in size; walls are 3–4 mm thick	–	Not found in the holotype	Portugal (depth unknown)
*L. lynceus* Schmidt, 1870 **(**MNHN-IP-2008-93)	Foliate to undulate polymorphic masses, with large oscules in the outer surface (243–269 µm) of the sponge and small pores in the external one (68–145 µm); colour beige to brown	90–93 mm wide; walls 5–12 mm thick	156–179–223 µm (*n* = 6) long and 8.4–19.4–49.9 µm thick (*n* = 30)	up to 1mm long and 8.5–9.6–10.7 µm thick (*n* = 5)	Gorringe seamount (305–320 m depth)
^2^*Leiodermatium pfeifferae* (Carter, 1873) (unknown type)	Flattish, cabbage-like, infoliated, with branched sinuous laminae, vertically, widely separated, and proliferous; ostia (vents) are little raised on papillary eminences and scattered over the inner of the laminae; pores are on the outer laminae	360 mm in diameter and 280 mm vertical diameter; walls 6–17 mm thick	–	Fusiform, growing on the edge/margins of the specimen (measurements were not given)	Madeira (684 m depth)
*L. tuba* sp. nov. (Holotype MNHN-IP-2018-72)	Large sponges, lamellate vase to contorted walls, sometimes forming a tube, with smooth and similar surfaces; colour is beige to brown	138 mm long and 93 mm wide; walls 4–5 mm thick	141–173–211 (*n* = 4) × 12.1–18.7–31.0 µm (*n* = 19)	up to 1 mm long and 5.9–8.1–9.8 µm thick (*n* = 3)	Gorringe seamount (805–830 m depth)
*L. tuba* sp. nov. (Paratype MNHN-IP-2018-73)	Small fragment, of tubular shape, with thin walls; outer surface is smooth but with a stripe appearance due the water canals underneath the surface; inner surface has a white appearance given the numerous oxeas piercing the surface	65 mm long and 25 mm wide; walls are 5–6 mm thick			Gorringe seamount (805–830 m depth)
^3^*L. deciduum* (Schmidt, 1879) (Holotype MZUS PO167). *Incertae sedis*	Ear shaped, or irregular vase shaped sponge; upper side of chaonosomal skeleton, with numerous oscula, 500 µm in diameter; lower side of choanosome with numerous pores, 200–250 µm in diameter	35 mm high, 32 mm wide; walls 10 mm thick	–	–	Gulf of Mexico (183–1,472 m depth)

**Notes:**

1 Redescription in Pisera & Lévi (2002i).

2 Carter (1873).

3 Pisera & Lévi (2002d): where the authors state that *Poritella decidua* Schmidt, 1879 seems to be a synonym of Leiodermatium, but the specimens are considered *incertae sedis* due the bad condition of the material.

‘–’ indicates the information was not given in the description.

**Synonym.** *Azorica pfeifferae var. tenuilaminaris* Sollas, 1888 (genus transfer and junior synonym).

**Material examined.** MNHN-IP-2018-93 (1988-09-24, Gorringe Seamount, beam trawl, st. CP20, 36°33.7′N, 11°30.1′W, 305–320 m, *Seamount 1* campaign), MNHN-IP-2008-239 (1993-01-16, Hyères Seamount, epibenthic Warén dredge, st. DW182, 31°23′N, 28°54′W, 480 m, *Seamount 2* campaign).

**Comparative material examined.** *L. tuba* sp. nov. (holotype MNHN-IP-2018-72, Gorringe Seamount; paratype MNHN-IP-2018-73, Gorringe Seamount).

**Diagnosis.** Foliate to undulate polymorphic masses, with large openings in the outer surface of the sponge and small openings in the inner surface.

**Description (MNHN-IP-2018-93).** Large foliate to undulate irregular masses, with thick lamellas, 5–12 mm, that in some cases can form cups/funnels (Fig. 3C); inner and outer surfaces are different from each other, and it is possible to distinguished at naked eye; outer surface has larger openings slightly elevated from the surface, 243–269 µm in diameter, (Figs. 3C, 30A and 30C) while the inner surface is smooth with small openings, 68–145 µm in diameter, evenly distributed (Figs. 3C, 30B and 30D); both surfaces are heavily protruded by long oxeas; colour varies from beige to brown in ethanol.

**Skeleton.** A very intricate, irregular and dense mesh of rhizoclones desmas extremely branched and spiny (Figs. 31A–31C); the body of this sponge is mainly built of desmas, giving them a stony consistency; near the openings and water canals, the arms of the desmas are more elongated; large oxeas cross the skeleton and perforate the surface; no microscleres.

**Spicules (MNHN-IP-2018-93).**

1. **Rhizoclones**, very spiny arms with multifurcating spines tips, 156–179–223 µm long and 8.4–19.4–49.9 µm thick (Figs. 31A–31F); zygosis is complex and robust (Fig. 31E).
2. **Oxeas**, smooth, straight or curved, up to 1 mm long and 8.5–9.6–10.7 µm thick.

**Distribution.** These specimens were found on the Gorringe and Hyères Seamounts, between 305 and 480 m depth.

**Remarks.** Within Tetractinellida, the genus *Leiodermatium* is particularly difficult from a taxonomic standpoint, given the few characters available to distinguish and describe the different species. In the North Atlantic, only two species have been described to date—*L. lynceus* Schmidt, 1870 and *L. pfeifferae* (Carter, 1873); the former from specimens collected off the coast of Portugal, and the later from Madeira island i.e. both from the NEA but unknown depths. Later, Carter (1876) formally explained the differences between these two species: (1) *L. lynceus* has large oscula located on outer surface while in *L. pfeifferae* they are on the inner surface; (2) *L. pfeifferae* has numerous fusiform oxeas on the edge of the laminae, while *L. lynceus* has “isolated acerates” (Schmidt, 1870) (however they were not found in the redescription of the holotype *L. lynceus* (Pisera & Lévi, 2002i)). Another important detail, is the difference between the thickness of the laminae on both species, *L. lynceus* has thinner (3–4 mm) laminae compared to *L. pfeifferae* (6–17 mm; see Table 5).

In addition to these two currently recognized species, *Poritella deciduum* (Schmidt, 1879), was also assigned to this genus (Lendenfeld, 1903) but this allocation is considered questionable (Pisera & Lévi, 2002i). Also, Sollas (1888) reported a number of varieties of *L. pfeifferae* from the material collected in the course of the Challenger expedition in the Atlantic, viz. *A. pfeifferae tenuilaminaris* (Bahia, Brazil, unknown depth) and *A. pfeifferae tenuilaminaris osculis disjunctis* (Bermuda, 795–1965 m depth). However, the material was deciduous and therefore the descriptions are incomplete (see also review in Kelly, 2007). Records of *L. lynceus* and *L. pfeifferae* for the western Atlantic (e.g. Van Soest & Stentoft, 1988) need to be carefully re-assessed, as they may represent different and likely undescribed species given that several putatively new *Leiodermatium* species have been reported for the tropical western Atlantic (Schuster et al., 2019) but still lack formal description. Topsent (1892) reports one specimen of *Azorica pfeifferae* for the Azores (st. 234, 454 m depth) with a strong blue coloration. However, from the illustration provided, it appears that the specimen has elevated openings on the external surface, thereby conforming to *L. lynceus*.

The specimens analysed in this study are very similar to the holotype of *L. lynceus* regarding the morphology, surfaces and the ornamentation of the desmas. The only difference lays on the size of the openings: the holotype has large oscula on the outer surface, 500–750 µm in diameter, while in our specimen oscula are 243–269 µm in diameter; the same happens in relation to the pores of the inner surface of the holotype, which are 156–188 µm in diameter, against 68–145 µm in our specimen (Table 5).

***Leiodermatium tuba* sp. nov.**

**Figure 32:**
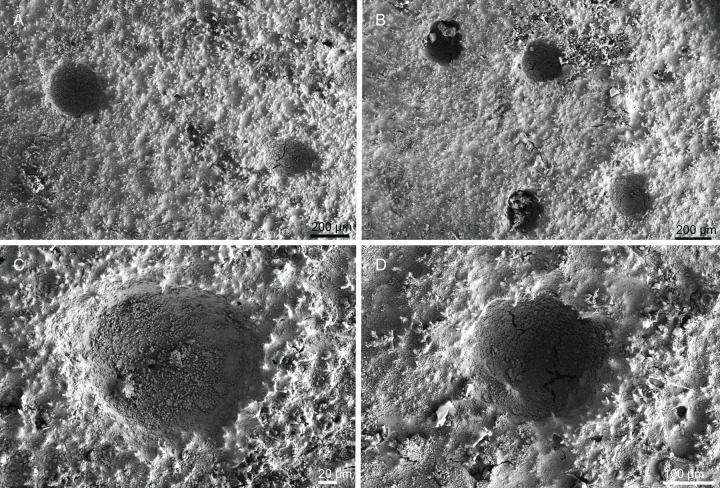
Surface of *Leiodermatium tuba* sp. nov., holotype MNHN-IP-2018-72. (A) Outer surface with depressed and slightly larger pores, (B) inner surface, with small and depressed pores, (C) detail of a pore from the outer surface, (D) detail of a pore from the inner surface.

**Figure 33:**
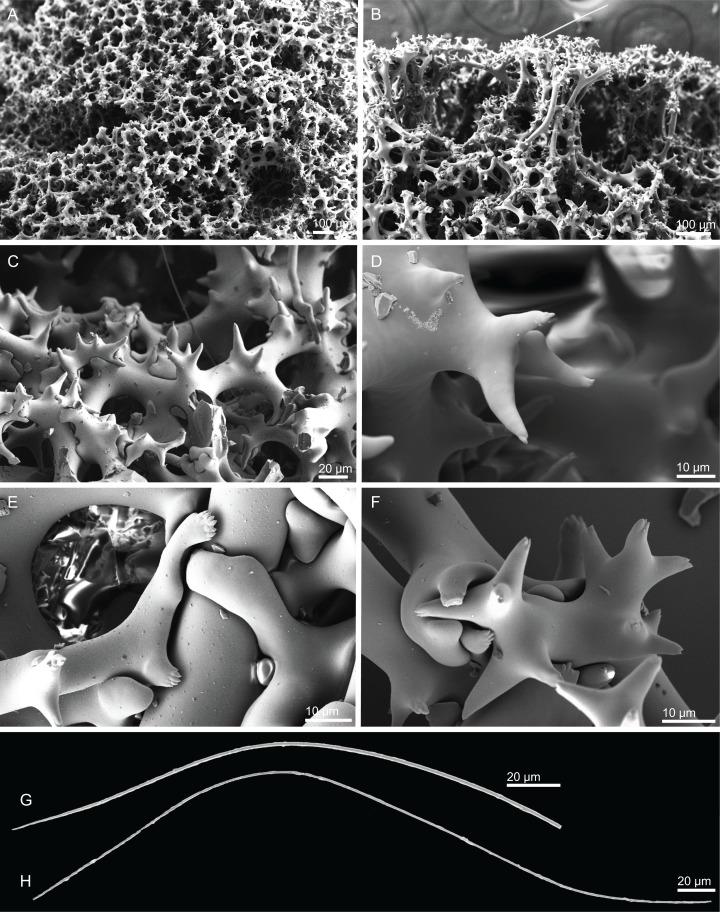
Skeleton and spicules of *Leiodermatium tuba* sp. nov., holotype MNHN-IP-2018-72. (A) Overview of choanosomal desmas showing the water cannals, (B) rhizoclones desmas, (C) ornamentation of the rays of the desmas, (D) close up on the ornamentation of the desmas, (E) zygosis, (F) zygosis and sculpture of rhizoclones, (G) and (H) oxeas.

Figures 3D, 32–33 and Table 5

urn:lsid:zoobank.org:act:041DAB82-B538-4EB9-A43A-1E3E79B67CF8

**Holotype.** MNHN-IP-2018-72 (1988-09-23, Gorringe Seamount, beam trawl, st. CP11, 36°26.4′N, 11°40.2′W, 805–830 m, *Seamount 1* campaign).

**Paratype.** MNHN-IP-2018-73 (1988-09-23, Gorringe Seamount, beam trawl, st. CP11, 36°26.4′N, 11°40.2′W, 805–830 m, *Seamount 1* campaign).

**Other material.** MNHN-IP-2018-74 (1988-09-25, Gorringe Seamount, epibenthic Warén dredge, st. DW25, 36°49.7′N, 11°03.3′W, 970–1,035 m, *Seamount 1* campaign); MNHN-IP-2018-75 (1988-09-23, Gorringe Seamount, beam trawl, st. CP11, 36°26.4′N, 11°40.2′W, 805–830 m, *Seamount 1* campaign); MNHN-IP-2018-76 (1988-09-23, Gorringe Seamount, beam trawl, st. CP11, 36°26.4′N, 11°40.2′W, 805–830 m, *Seamount 1* campaign); MNHN-IP-2008-235 (1993-01-31, Plato Seamount, epibenthic Warén dredge, st. DW242, 33°12′N, 28°57′W, 710 m, *Seamount 2* campaign); MNHN-IP-2008-237 (1993-02-03, Atlantis Seamount, epibenthic Warén dredge, st. DW265, 34°29′N, 30°36′W, 545 m, *Seamount 2* campaign); MNHN-IP-2008-249b (1993-01-19, Hyères Seamount, epibenthic Warén dredge, st. DW202, 31°16′N, 28°43′W, 640 m, *Seamount 2* campaign); MNHN-IP-2008-253 (1993-01-11, Great Meteor Seamount, epibenthic Warén dredge, st. DW159, 29°44′N, 28°20′W, 330 m, *Seamount 2* campaign); MNHN-IP-2008-255 (1993-01-06, Gran Canaria, epibenthic Warén dredge, st. DW130, 28°09′N, 15°53′W, 660 m, *Seamount 2* campaign).

**Comparative material examined.** *L. lynceus* (MNHN-IP-2018-93, Gorringe Seamount; MNHN-IP-2008-239, Hyères Seamount).

**Diagnosis.** Massive lamellate vase to contorted walls, sometimes forming a cone, with smooth and similar surfaces.

**Description (holotype MNHN-IP-2018-72).** Lamellate vase with contorted thin walls, 4–5 mm, occasionally forming a cone (Fig. 3D); this specimen consists of three fragments, the largest one is 138 mm long and 93 mm wide; surfaces are identical when observed with the naked-eye, given they are both smooth, but some differences can be noticed when observed under the stereomicroscope: outer surface has slightly larger depressed openings, 266–322 µm in diameter, (Figs. 32A and 32C) and a striated appearance due to the water canals underneath the surface; inner surface (Figs. 32B and 32D) has a whitish appearance caused by the presence of numerous oxeas covering the smaller depressed openings; openings are 186–261 µm in diameter; specimen coloration varies from light beige to brown in ethanol.

**Skeleton.** There is no clear distinction between the ectosome and choanosome since there is no special arrangement of spicules or different spicules in the ectosome; choanosomal skeleton is composed by very spiny rhizoclones desmas, forming a complex, branching and compact network (Figs. 33A and 33B); other megascleres are oxeas across the skeleton; microscleres are not present.

**Spicules (holotype MNHN-IP-2018-72).**

1. **Rhizoclones**, spiny, 141–173–211 × 12.1–18.7–31.0 µm in size, with single to multifurcate spiny tips (Figs. 33A–33F); zygoses are strong where several clones can articulate with each other, making the skeleton very dense and robust (Figs. 33E and 33F).
2. **Oxeas**, thin, curved, up to 1 mm long and 5.9–8.1–9.8 µm thick (Figs. 33G and 33H).

**Etymology.** From the Latin *tubae*= trumpet; since some lamellas in this species have a conical shape resembling a trumpet.

**Distribution.** The type locality is the Gorringe Seamount at 805–830 m depth. Other specimens were found in Plato, Hyères, Atlantis and Gorringe Seamounts between 545 and 1,035 m, and in Gran Canaria at 660 m.

**Remarks.** *L. tuba* sp. nov. exhibits a distinct external morphology and surface ornamentation compared to the other two *Leiodermatium* species recorded for the North Atlantic, i.e. *L. lynceus* and *L. pfeifferae*. Firstly, in *L. tuba* sp. nov. both surfaces look similar at the naked eye (smooth and with slightly depressed openings; Fig. 32) whereas in *L. lynceus* and *L. pfeifferae*, the openings are elevated (depending on the surface) and this is a very distinctive feature (see above remarks under *L. lynceus*). Additionally, the inner surface of *L. tuba* sp. nov. is pierced by numerous oxeas providing a whitish colour to this surface. Pisera & Lévi (2002h) discussed the possibility of a new species of *Leiodermatium* being reported as *L. lynceus* due to the absence of larger oscules on the outer side. However, it is not clear from their account to which specimens they were referring to nor their characteristics. Perhaps they conform to *L. tuba* sp. nov. here described.

Another important observation is the bathymetric range where the *Leiodermatium* spp. were collected in this study. *L. tuba* sp. nov. was usually found deeper (330–830 m depth) than *L. lynceus* (305–320 m depth) (see “Diversity” section and Supplemental Material S1).

**Family SIPHONIDIIDAE Lendenfeld, 1903**

**Genus *Siphonidium*Schmidt, 1879**

**Synonymy.** *Siphonidiella* Burton, 1928 (junior synonym), *Tremaulidium* Schmidt, 1879 (junior synonym).

**Diagnosis.** Siphonidiidae with fistules; choanosmal megascleres are rhizoclones desmas, exotylostyles and/or styles (emended after Pisera & Lévi (2002f)).

**Definition.** Polymorphic Siphonidiidae, encrusting, massive irregular, hemispherical or irregularly cylindrical to club-shape with fistules; without special ectosomal spicules; rhizoclone desmas, exotylostyles and styles as choanosomal spicules (emended after Pisera & Lévi (2002f)).

**Type species.** *Leiodermatium ramosum* Schmidt, 1870 (type by original designation).

***Siphonidium elongatus* sp. nov.**

**Figure 34:**
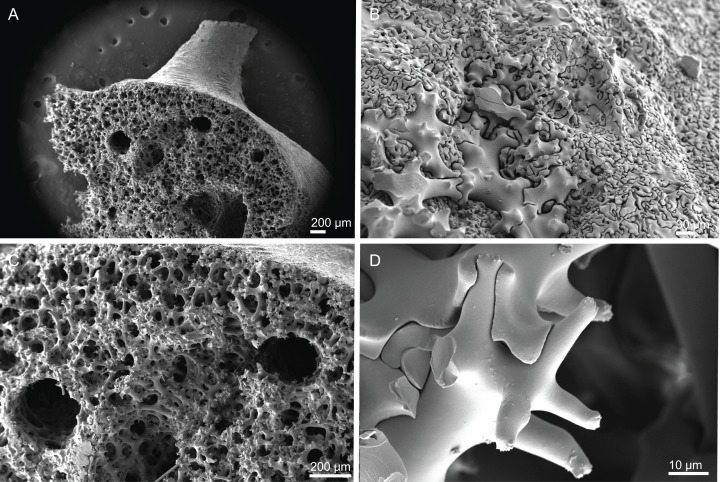
Surface and skeleton of *Siphonidium elongatus* sp. nov., holotype MNHN-IP-2008-236. (A) Overview of a fragment of the specimen showing a fistule, (B) surface of the sponge composed of modified desmas, (C) overview of the chonosomal desmas showing the water canals in the rhizoclones, (D) zygosis showing the ornamentation of desmas tips.

**Figure 35:**
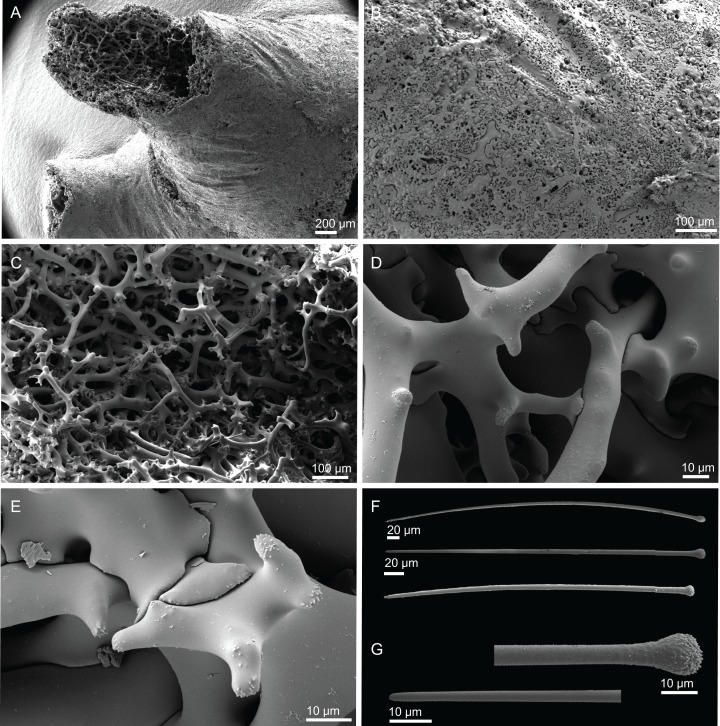
Surface, skeleton and spicules of *Siphonidium elongatus* sp. nov., holotype MNHN-IP-2008-236. (A) Overview of a fistule, (B) close up of the modified desmas from the surface of the fistule, (C) loose rhizoclones of the fistule, (D) detail of the desmas, (E) close up of the ornamentation of the desmas of the fistules, (F) exotylostyles, (G) detail of the spiky pin-shaped head and tip of the exotylostyles.

Figures 3E, 34–35 and Table 6

**Table 6:** Comparative table of external morphology and spicular micrometries of all *Siphonidium* species recorded in the North Atlantic Ocean and Mediterranean Sea. Spicule measurements (*n* = 30 unless stated otherwise) are presented as minimum–mean–maximum. Data compiled from the original descriptions, or subsequent re-descriptions of type material (marked with numbers).

	Habitus	Size	Rhizoclones	Exotylostyles	Strongyles	Locality
^1^*Siphonidium ramosum* (Schmidt, 1879) (Holotype MCZ 6321, 6322)	Small, irregular massive to cylindrical, with numerous small fistules	20–55 mm high, 10 mm wide; fistules are 1–2 mm in diameter	Massive rhizoclones, strongly tuberculated, 180–220 µm in size	With spinose heads: 160–220 µm × 2–3 µm	Not present	Florida (depth unkonw)
^2^*Siphonidium ramosum* (Schmidt, 1879)	–	–	–	800–1,000 µm long, 4–6 µm thick	–	Azores (349–793 m)
^3^*Siphonidium dubium* Lévi, 1959 (Holotype)	Massive and hard sponge with a large base, that its subdivided into three lobes 2 cm long barely separated, that ended on a flat surface; surface reticulated and covered by numerous pores; ostia, 1–1.5 cm in diameter; colour light beige when alive and dark brown in ethanol	–	Compact	Not present	Abundant, grouped perpendicular to the surface, tip slightly rugose and rounded, 600–800 µm	Principe, Gulf of Guinea (50 m depth)
*Siphonidium geminum* (Schmidt, 1879) (Holotype MNHN DT 2194)	Flat and irregular incrusting basis bearing simple or bifurcate cone shaped prolongations with round ends; surface is covered by a finely corrugated cuticula	–	Irregular, like “three-roots” and later they are bumpy and hard	–	Not present	Gulf of Mexico (240 m depth)
*Siphonidium elongatus* sp. nov. (Holotype MNHN-IP-2008-236)	Cylindrical to arborescent, sometimes bulb shape; surface is smooth and exhibits fistules that are often closed but may be open; colour is beige to brown	33–49 × 2–9 mm in size	123–197–267 × 10.4–23.5–40.3 µm; desmas branches, 13.8–30.9–88.2 µm long	173–363–504 × 2.9–5.1–6.6 µm (*n* = 4)	Not present	Atlantis seamount (545 m depth)
*Siphonidium elongatus* sp. nov. (Paratype MNHN-IP-2018-79)	Cylindrical, elongated with several fistules; colour is brown	54 × 4–5 mm in size	129–210–326 × 12.1–22.1–34.0 µm; desmas branches, 16.8–45.4–83.7 µm long	248–393 × 6.5–12.4 µm (*n* = 3)	Not present	Gorringe seamount (605–675 m depth)

**Notes:**

1 Redescription in Pisera & Lévi (2002g).

2 Topsent (1904).

3 Schmidt (1879).

urn:lsid:zoobank.org:act:26B193F9-2588-4479-ACB2-27AD1945DEE4

**Holotype.** MNHN-IP-2008-236 (1993-02-03, Atlantis Seamount, epibenthic Warén dredge, st. DW265, 34°29′N, 30°36′W, 545 m, *Seamount 2* campaign).

**Paratype**. MNHN-IP-2018-79 (1988-09-26, Gorringe Seamount, beam trawl, st. CP28, 36°38′N, 11°29.8′W, 605–675 m, *Seamount 1* campaign).

**Other material.** MNHN-IP-2008-232 (1993-01-06, Gran Canaria, epibenthic Warén dredge, st. DW128, 28°08′N, 15°52′W, 470 m, *Seamount 2* campaign), MNHN-IP-2008-245 (1993-01-16, Hyères seamount, epibenthic Warén dredge, st. DW182, 31°23′N, 28°54′W, 480 m, *Seamount 2* campaign), MNHN-IP-2008-256 (no data), MNHN-IP-2018-80 (no data), MNHN-IP-2018-81 (1988-09-26, Gorringe Seamount, beam trawl, st. CP28, 36°34.9′N, 11°28.4′W, 605–675 m, *Seamount 1* campaign), MNHN-IP-2018-78 (1988-09-24, Gorringe seamount, epibenthic Warén dredge, st. DW21, 36°34.9′N, 11°28.4′W, 460–480 m, *Seamount 1* campaign), MNHN-IP-2018-82 (1988-10-08, Lion seamount, epibenthic Warén dredge, st. DW63, 35°1.4′N, 15°34.4′W, 630 m, *Seamount 1* campaign), MNHN-IP-2018-83 (1988-09-26, Gorringe seamount, epibenthic Warén dredge, st. CP28, 36°38.0′N, 11°29.8′W, 605–675 m, *Seamount 1* campaign).

**Diagnosis.** Polymorphic sponge, cylindrical to arborescent, with several fistules; rhizoclones with slim arms ornamented with microspines along the edges; exotylostyles to styles as other choanosomal megascleres.

**Description (holotype MNHN-IP-2008-236).** Polymorphic sponge, cylindrical to arborescent Siphonidiidae, sometimes of bulb shape, attached by the base to the substrate; small, 33–49 mm high, thin, 2–9 mm wide (but can be 14 mm wide); surface is smooth and exhibits fistules spread through the sponge pointed in several directions, 1–8 mm long and 1–4 thick (Fig. 3E); fistules are usually close-ended, but when open, it is possible to see the subdermal water canals emerging from the interior of the sponge; extremely hard sponge (stony consistency); colour varies from beige to brown in ethanol.

**Skeleton.** No clear distinction of the spicules between the ectosome and choanosome, with exception of the desmas of the surface that are different from the interior of the skeleton: a layer of flattened, fused and modified desmas, resembling a puzzle, constitutes the surface of the sponge (Figs. 34B and 35B); these modified desmas, resembling a shield, contribute to the hardness of this species; some wrinkles can also be observed on the surface of the sponge (Fig. 35A); choanosome is formed by an extremely dense, compact and irregular net of rhizoclone desmas, exotylostyles and rarely styles, crossing through the skeleton; several water canals can be observed in a cross section of the sponge, as large holes (Figs. 34A and 34C) surrounded by the desmas that here are slightly more elongated (Fig. 34C); desmas from the fistules are different from the ones in the ‘body’ of the sponge, i.e., usually the desmas of the fistules are longer and looser (Fig. 35C) while in the ‘body’ they are very dense and compact (Fig. 34C).

**Spicules (holotype MNHN-IP-2008-236).**

1. **Rhizoclone desmas**, extremely dense especially near the surface, 123–197–267 μm long and 10.4–23.5–40.3 μm wide (Figs. 34A–34C); clones are smooth with several finger-like branches, that can be smooth or ornamented with microspines on the tips, 13.8–30.9–88.2 μm long (Figs. 34D, 35D and 35E); zygoses can be formed by several rays or just some, but it is always solid and complex (Figs. 34F and 35E);
2. **Exotylostyles**, pin-shaped, with spiny heads and pointed tips, straight or slightly curved, not very abundant, 173–363–504 μm in length and 2.9–5.1–6.6 μm in width (Figs. 35F and 35G); some exotylostyles look underdeveloped and resemble styles.

**Distribution.** *Siphonidium elongatus* sp. nov. was found in the Atlantis, Hyéres, Lion, and Gorringe seamounts, and in Gran Canaria, between 470 and 675 m depth

**Etymology.** From the latin *elongatus* = elongated, due to an elongated shape of the desmas, especially those composing the fistules.

**Remarks.** Three species of *Siphonidium* have been described in the Atlantic Ocean, and only one, *S. ramosum*, has been reported for both sides of the North Atlantic (Schmidt, 1879; Topsent, 1928, 1904, 1892; Van Soest, 2017; Van Soest & Stentoft, 1988) and Mediterranean Sea (Longo, Mastrototaro & Corriero, 2005; Vacelet, 1969; Zibrowius & Taviani, 2005). With the redescription of *S. ramosum* in (Pisera & Lévi, 2002g), a detailed account of the external morphology and spicules was given, allowing a better definition of the species. Despite the relatively similar habitus of *S. ramosum* and *S. elongatus* sp. nov., the main difference between these two species relies on the desmas morphology and ornamentation: *S. elongatus* sp. nov. has very spiny rhizoclones with slim arms ornamented with microspines on the edges, contrasting with the tuberculated rhizoclones of *S. ramosum*. Another distinct feature, is the presence of styles in *S. elongatus* sp. nov. (even though they are rare) that were never mentioned in the redescription of *S. ramosum*. Furthermore, when *S. elongatus* sp. nov. is compared with the other North Atlantic species, its external morphology and spicules differ: *S. dubium* Lévi, 1959 is a massive sponge with a large base, subdivided into three lobes and the only one within the genus with strongyles; *S. geminum* (Schmidt, 1879) has a flat and irregular incrusting base with simple or bifurcated cone shape.

Topsent (1904) presented a small description of *S. ramosum* from several specimens found in the Azores. In his account, the shape and the ornamentation of the desmas are not explicitly described or illustrated, but the spicules sizes are given and are much larger than the ones described by Schmidt (1879) from material collected in the Gulf of Mexico (Table 6). The spicules sizes in *S. elongatus* sp. nov. are more similar to the ones in *S. ramosum* described by Schmidt than to the one described by Topsent. It was previously stated by Van Soest (2017), that the *S. ramosum* reported from the Azores, is most likely a different species due to the difference in the spicules sizes when compared to the type material. A revision of Topsent’s material would be required to clarify this question.

**Order BUBARIDA Morrow & Cárdenas, 2015**

**Family DESMANTHIDAE Topsent, 1893**

**Genus *Petromica*Topsent, 1898**

**Synonymy.** *Monanthus* Kirkpatrick, 1903 (junior synonym).

**Diagnosis.** Massive, encrusting or globular shape Desmanthidae with desmas branching in various planes forming a loosely articulated or non-articulated choanosomal skeletal structure. (List-Armitage & Hooper, 2002; Pisera & Lévi, 2002h).

**Definition.** Massive, encrusting or globular in shape, with or without fistule-like papillae. Surface smooth, hispid, conules can be present. Compressible to rigid, or soft to fragile sponges. Acrepid or monocrepid smooth desmas, branched in several planes. Desmas can be isolated, non-articulated, fused, or dispersed in the ectosome and choanosome; zygomes vary from simple to complex; zygosis when present, is rarely fully articulated in the skeleton turning into a loose skeleton. Other megascleres are oxeas, where the tips can vary from sharp to blunt. Microscleres not present (List-Armitage & Hooper, 2002; Muricy et al., 2001; Pisera & Lévi, 2002h)

**Type species.** *Petromica* (*Petromica*) *grimaldii* Topsent, 1898 (type by monotype).

**Subgenus *Petromica*Topsent, 1898**

**Diagnosis.** Firm and rigid sponge, with or without papillae, with acrepid or monocrepid desmas that can form a loose or well-formed skeleton. Oxeas present and variable in size (List-Armitage & Hooper, 2002).

***Petromica* (*Petromica*) *grimaldii*Topsent, 1898**

**Figure 36:**
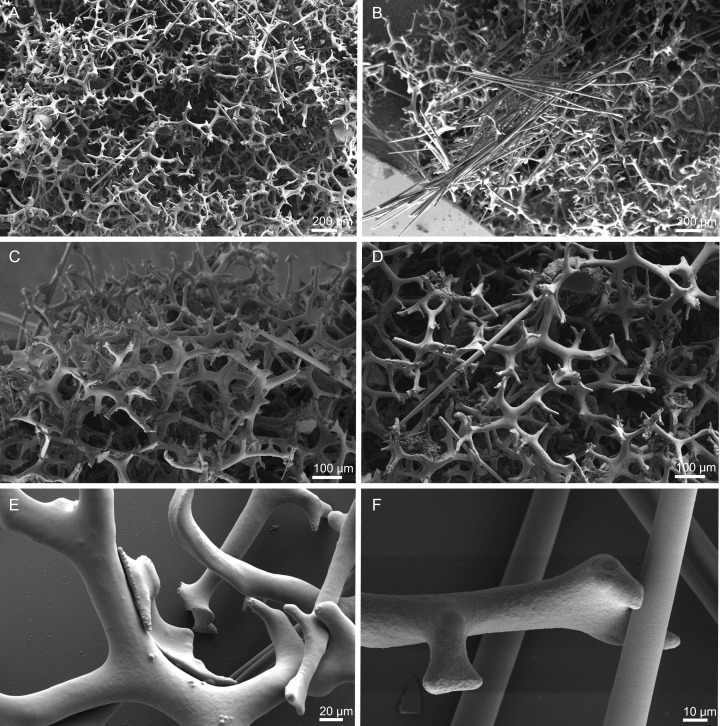
Skeleton of *Petromica (Petromica) grimaldii* Topsent, 1898, specimen MNHN-IP-2008-92. (A) Overview of monocrepid desmas, (B) bundles of anisoxeas and strongyloxeas, (C) monocrepid desmas, (D) detail of anisoxeas and strongyloxeas crossing the desmas, (E) zygosis, (F) zygosis between a desma and an oxea.

**Figure 37:**
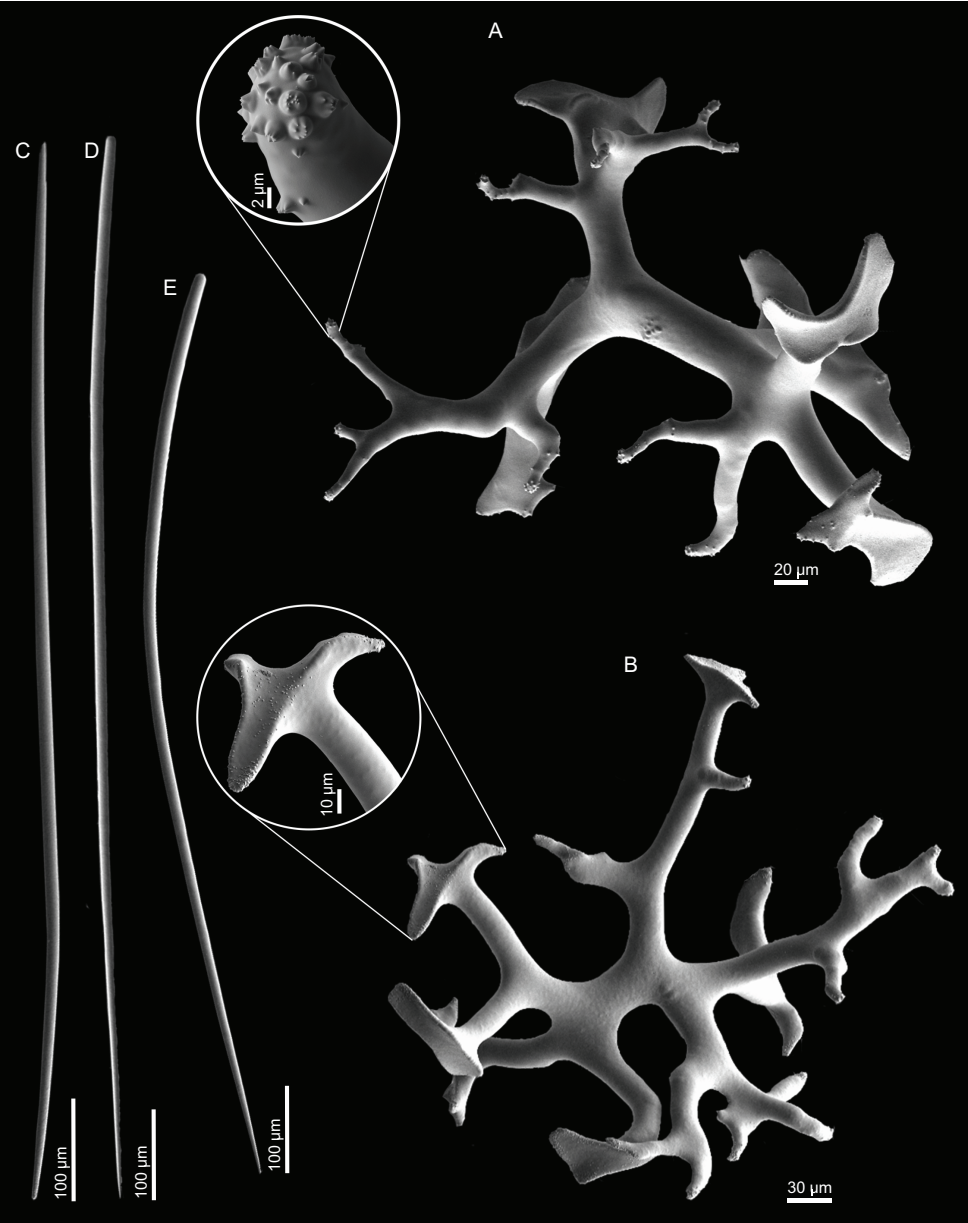
Spicules of *Petromica (Petromica) grimaldii* Topsent, 1898, specimen MNHN-IP-2008-92. (A) Monocrepid desma pointing a detail on the ornamentation of the tips of the desma, (B) monocrepid desma showing a close up of the zygome, (C) Anisoxea, (D) and (E) strongyloxeas.

Figures 3F, 36–37 and Table 7

**Table 7:** Comparative table of external morphology and spicular micrometries of all *Petromica* species recorded in the North Atlantic Ocean. Spicule measurements (*n* = 30 unless stated otherwise) are presented as minimum–mean–maximum. Data compiled from the original descriptions, or subsequent re-descriptions of type material (marked with numbers).

	Habitus	Size	Monocrepid desmas	Anisoxeas	Strongyloxeas	Locality
^1^*Petromica* (*Chaladesma*) ciocalyptoides (Van Soest & Zea, 1986) (Holotype RMNH 1309)	Basal mass buried in the sand, with proeminent tapering fistules; colour pale yellow-orange alive and white in alcohol	1 cm thick; fistules are large, 100 mm long, 3–9 mm thick	Smooth, long clads, 600–700 µm, clads up to 300 µm, epirhabd 180–300 × 10–28 µm; no zygosis	–	Sometimes modified to styles, 378–592 × 5–22 µm	Saba Bank, Colombia (34 m depth)
^2^*Petromica* (*Chaladesma*) *citrina* Muricy, Hajdu, Minervino, Madeira & Peixinho, 2001 (Holotype MNRJ 580)	Thickly encrusting to massive, irregular, with small cone-shaped or digitiorm surface projections and large papillae (1–9); colour alive bright orange-yellow, pale yellow in ethanol	Base: 30–90 × 16–60 mm wide, 4–20 mm thick	180–337–620 µm, epirhab 40–87.4–190 × 9.8–32.6 µm; cladii 50–126–300 µm. long	–	Usually both extremities are acerate, but occasionally stylote or strongylote can be present, 320–527–780 × 3.2–26 µm	São Sebastião island, Brazil (25 m depth)
^3^*Petromica* (*Petromica*) *grimaldii* Topsent, 1898 **(**Holotype MNHN DT 850)	Massive sponge, large in the base and slimer on top, covered with conules	Very variable, large specimens up to 40 mm high and 30 mm diameter	Up to 570 µm in size	956–1250 × 23–30 µm	700–1280 × 23 µm	Azores (200–599 m depth)
*Petromica* (*Petromica*) *grimaldii* (MNHN-IP-2008-92)	Massive sponge with a soft and rugose surface; papillae absent	Small, 21 mm height and 14 mm width	347–499–652 µm × 8–22–80 µm	890–1,213–1,376 × 14.5–22.5–28.9 µm	541–1,122–1,561 × 13.1–21.1–33.2 µm (*n* = 29)	Gorringe Seamount (255–265 m depth)

**Notes:**

1 Van Soest & Zea (1986).

2 Muricy et al. (2001).

3 Redescription in Pisera & Lévi (2002e).

**Synonym.** *Petromica grimaldii* Topsent, 1898 accepted, alternate representation (subgenus assignment).

**Material.** MNHN-IP-2018-92 (1998-09-24, Gorringe Seamount, epibenthic Warén dredge, st. DW16, 36°31.1′N, 11°32.5′W, 255–265 m, *Seamount 1* campaign).

**Diagnosis.** Small sponge with a conulose surface and no papillae; desmas are monocrepid.

**Description (MNHN-IP-2018-92).** Fragile, soft, massive sponge with a soft and conulose surface; small, 21 mm height and 14 mm width; colour white in ethanol (Fig. 3F); specimen in poor condition.

**Skeleton.** No clear distinction between ectosome and choanosome; skeleton is composed of smooth monocrepid desmas (Figs. 36A–36D), poorly articulated (Fig. 36E), forming a loose, confuse and irregular skeleton; other megascleres are anisoxeas and strongyloxeas, rarely tylostyles, usually arranged in bundles (Fig. 36B); it is also possible to observe a zygosis between the desmas and the oxeas (Fig. 36F); microscleres are absent.

**Spicules (MNHN-IP-2018-92).**

1. **Monocrepid desmas**, smooth, with branches in several planes, except for the tips which can have some ornamentation with a spiny appearance (Figs. 36 and 37A), 347–499–652 × 8–22–80 µm in size; tips are 45–82–147 × 10.6–15.2–30.8 µm in size; zygomes are spiny, mainly in the inner part, about 57–118–207 µm in size (Fig. 37B);
2. **Anisoxeas**, very abundant, fusiform, smooth, with acerate tips, 890–1,213–1,376 µm long and 14.5–22.5–28.9 µm thick (Fig. 37C);
3. **Strongyloxeas**, smooth, can be straight or curved, with one acerate tip and one blunt tip, 541–1,122–1561 µm long and 13.1–21.1–33.2 µm wide (Figs. 37D and 37E).

**Distribution.** This specimen was found on the Gorringe seamount between 255 and 265 m depth.

**Remarks.** *Petromica* is a widely distributed genus, and so far, eight species have been described. In the North Atlantic, three species have been reported, *P. (Chaladesma) ciocalyptoides* and *P. (Chaladesma) citrina* to the NWA and *P*. (*Petromica*) *grimaldii* from the NEA and MED (Table 7). *P*. (*P*.) *grimaldii* was first described from the Azores archipelago by Topsent (1898) where it was found to be a very common sponge, collected throughout the archipelago between 200 and 914 m depth (Topsent, 1928, 1904, 1898). This species has been also reported from the MED (Boury-Esnault, Pansini & Uriz, 1994; Pulitzer-Finali, 1972) and since microspine desmas’ terminations were absent, *P*. (*P*.) *massalis* Dendy, 1905 (a species from the Indian Ocean) and *P*. (*P*.) *grimaldii* were synonymized (Pulitzer-Finali, 1972). According to Muricy et al. (2001), these microspines are not present in all desmas in the same specimen and they can be rare. Therefore, the absence of microspines in the desmas is not enough to distinguish one species from another. A more detailed examination of the specimens from the MED would be necessary to allow to clarify this uncertainty (Muricy et al., 2001) and make sure the *Petromica* found in MED are in fact *P*. (*P*.) *grimaldii*. In the specimen examined in this study spicules sizes are very similar to those of the holotype (from the Azores) and the microspines in the termination of the desmas are present and very evident (Fig. 37).

## Diversity

**Figure 38:**
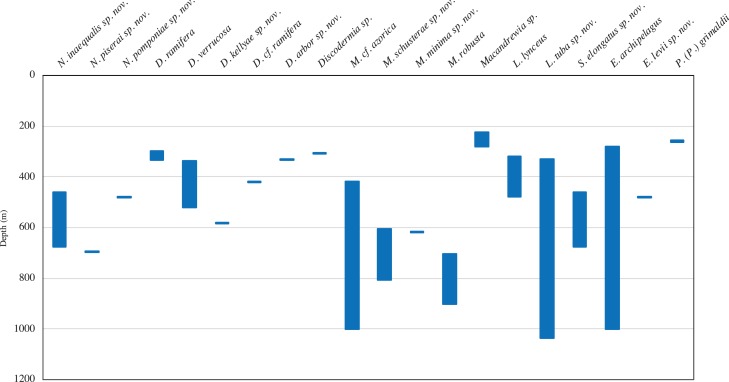
Bathymetric distribution of the lithistid demosponges collected during the *Seamount 1* and *Seamount 2* expeditions on the Northeast Atlantic seamounts.

The specimens described in the present work constitute the first records of lithistid demosponges for these two groups of NEA seamounts, except for *Exsuperantia archipelagus*. The Meteor seamount group harbours a more diverse lithistid fauna, 15 species, compared to the Lusitanian seamount group, where six species are recorded (Table 8). At a smaller scale, the Hyères seamount is the most diverse where eight species, namely *N. pomponiae* sp. nov., *M*. cf. *azorica*, *M. robusta*., *E. archipelagus*, *E. levii* sp. nov., *L. lynceus*, *L. tuba* sp. nov. and *S. elongatus* sp. nov. were found, followed by the Gorringe and Atlantis (six species), Plato and Great Meteor (five species), Tyro (three species) and Lion seamount (one species). Two specimens were found on the Antialtair and Ampère seamount (one on each) but it was not possible to identify them down to species level because they were small and incrusting specimens, possibly young individuals of *M. robusta*. The majority of the species have a restricted distribution and are found only in one or two seamounts, except *E. archipelagus, M*. cf. *azorica, M. schusterae* sp. nov., *L. tuba* sp. nov. and *S. elongatus* sp. nov., that are distributed between three to five different seamounts. Four species were also sampled in Gran Canaria, *M*. cf. *azorica* (480 m depth), *E. archipelagus* (660 m depth), *L. tuba* sp. nov. (660 m depth) and *S. elongatus* sp. nov. (470 m depth), the two latter representing the first records for the Canary Islands. New bathymetric records were also reported for three species, viz. *M. robusta* (705 m), *D. ramifera* (300–420 m), *D. verrucosa* (338–520 m) (Fig. 38).

**Table 8:** Overall distribution of lithistid demosponges in the Northeast Atlantic and Mediterranean Sea. Species found in this study (•) and records from the literature (○). Newly described species are highlighted in bold.

	Seamounts									Oceanic archipelagos				Continental shelf/slope		
**Species**	ATR	ATL	TYR	PLT	HYR	MET	LIO	GOR	AMP	AZO	MAD	SEL	CAN	PT	MED	MOR
CORALLISTIDAE Sollas, 1888																
*Corallistes elegantior* Schmidt, 1870														○		
*Corallistes masoni* (Bowerbank, 1869)											○		○			
*Isabella harborbranchi* Carvalho, Pomponi & Xavier, 2015													○			
*Neophrissospongia endoumensis* Pisera & Vacelet, 2011															○	
*Neophrissospongia nana* Manconi & Serusi, 2008															○	
*Neophrissospongia nolitangere* (Schmidt, 1870)										○	○	○	○		○	
*Neophrissospongia radjae* Pisera & Vacelet, 2011															○	
*Neoschrammeniella bowerbankii* (Johnson, 1863)											○				○	
***Neoschrammeniella inaequalis* sp. nov.**								•								
***Neoschrammeniella piserai* sp. nov.**				•												
***Neoschrammeniella pomponiae* sp. nov.**					•											
*Neoschrammeniella* sp.								•								
THEONELLIDAE Lendenfeld, 1903																
***Discodermia arbor* sp. nov.**						•										
***Discodermia kellyae* sp. nov.**				•												
*Discodermia polydiscus* (Bowerbank, 1869)													○	○		
*Discodermia polymorpha* Pisera & Vacelet, 2011															○	
*Discodermia ramifera* Topsent, 1892						•				○						
*Discodermia* cf. *ramifera*		•														
*Discodermia* sp.						•										
*Discodermia verrucosa* Topsent, 1928		•				•				○	○		○			
*Theonella annulata* Lendenfeld, 1907																○
MACANDREWIIDAE Schrammen, 1924																
*Macandrewia azorica* Gray, 1859										○		○	○			
*Macandrewia* cf. *azorica*		•	•		•								•			
***Macandrewia schusterae* sp. nov.**			•	•				•								
***Macandrewia minima* sp. nov.**						•										
*Macandrewia ramosa* Topsent, 1904										○						
*Macandrewia robusta* Topsent, 1904					•					○						
*Macandrewia* sp.	•								•							
PHYMARAPHINIIDAE Schrammen, 1924																
*Exsuperantia archipelagus* Carvalho and Pisera, 2018		•	•	•	•					○	○		•			
***Exsuperantia levii* sp. nov.**					•											
AZORICIDAE Sollas, 1888																
*Leiodermatium lynceus* Schmidt, 1870					•			•		○	○		○			○*
*Leiodermatium pfeifferae* (Carter, 1876)										○	○				○	
***Leiodermatium tuba* sp. nov.**		•		•	•	•		•					•			
SCLERITODERMIDAE Sollas, 1888																
*Aciculites mediterranea* Manconi, Serusi & Pisera, 2006															○	
*Microscleroderma lamina* Perez et al., 2004															○	
SIPHONIDIIDAE Lendenfeld, 1903																
*Gastrophanella phoeniciensis* Perez et al., 2004															○	
***Siphonidium elongatus* sp. nov.**		•			•		•	•					•			
*Siphonidium ramosum* (Schmidt, 1870)										?					○	
DESMANTHIDAE Topsent, 1893																
*Desmanthus incrustans* (Topsent, 1889)															○	
*Petromica* (*Petromica*) *grimaldii* Topsent, 1898								•		○					○	
*Sulcastrella tenens* (Vacelet, 1969)															○	
Total of species/taxa 36	1	6	3	5	8	5	1	6	1	11	7	2	10	2	15	1

**Notes:**

AMP, Ampere seamount; ATL, Atlantis seamount; ATR, Antialtair seamount; AZO, Azores; CAN, Canaries; GOR, Gorringe seamount; HYR, Hyères seamount; LIO, Lion; MAD, Madeira; MED, Mediterranean Sea; MET, Great Meteor seamount; MOR, Morocco; PLT, Plato seamount; PT, Portugal; SEL, Selvagens; TYR, Tyro seamount.

Sources of the literature records: Bowerbank (1869); Carter (1876); Carvalho, Pomponi & Xavier (2015); Carvalho & Pisera (2019); Cruz (2002); du Bocage (1869); Gray (1859); Johnson (1863); Lendenfeld (1907); Longo, Mastrototaro & Corriero (2005); Magnino et al. (1999); Maldonado et al. (2015); Manconi, Serusi & Pisera (2006); Manconi & Serusi (2008); Perez et al. (2004); Pisera & Vacelet (2011); Pulitzer-Finali (1972); Schmidt (1870); Topsent (1889, 1892, 1898, 1904, 1928); Vacelet (1969).

* Var *tenuilaminare* (Topsent, 1928).

?The assignment of the specimens examined by Topsent in Azores need to be revised in order to clarify if it is in fact *S. ramosum*.

Some of the examined material was of very small size and/or in poor condition, which hampered its identification to lower taxonomic levels. These specimens were therefore not identified and are not included in this manuscript (see Supplemental Material).

## Discussion

### Diversity and biogeographic patterns

With the present work, we describe for the first time the lithistid fauna of two seamount groups of the NEA, the Great Meteor and the Lusitanian seamounts. All of the 17 species here reported constitute new records for these seamounts and ten are new to science. The only exception is *E. archipelagus* previously reported for the Great Meteor Seamount as *Exsuperantia* sp. (Cárdenas et al., 2011). These 10 newly described species add to the 17 species previously reported for the NEA, representing an increase of approximately 60% of the lithistid diversity of this area. These findings show how understudied the fauna of these ecosystems is and suggests that additional species are likely to be found as survey efforts increase. It also concurs with previous studies made for other invertebrate groups based on material collected from the same seamounts where several new species were described (Berning, Harmelin & Bader, 2017; Cárdenas et al., 2018; George & Schminke, 2002; Gofas, 2007; Souto, Berning & Ostrovsky, 2016). The Great Meteor group, appears to harbour a more diverse lithistid fauna, with a total of 15 species (nine new to science), whereas in the Lusitanian group, six species were recorded (four new to science). Interestingly, only a relatively small proportion of the lithistid species known from the NEA (7 out of 17) were found during the present study. Finally, the finding of 19 large specimens of *M*. cf. *azorica* in the same station in the Hyères Seamount (st. DW202), suggests that this species may occur in relatively larger densities, possibly forming a sponge ground in this area. However, this would require verification with other sampling and observation tools such as remotely operated or autonomous underwater vehicles (ROV/AUV). Such finding would add on to the aggregations dominated by *Leiodermatium pfeifferae*, recently reported on three seamounts in the Western Mediterranean Sea (Maldonado et al., 2015), which suggests that some extant lithistids may still form highly structured habitats comparable to the Mesozoic reefs (Maldonado et al., 2015; Reid, 1967),

Several paradigms in seamount ecology, including the seamount endemism hypothesis, have been heavily debated in recent years, with some authors considering seamounts as places of high endemism (de Forges, Koslow & Poore, 2000), while others attributed the observed patterns to sample bias (Samadi et al., 2006; see also McClain, 2007; Rowden et al., 2010). In our study, the majority of the species (*Neoschrammeniella inaequalis* sp. nov., *N. piserai* sp. nov., *N. pomponiae* sp. nov., *Discodermia. arbor* sp. nov., *D. kellyae* sp. nov., *D. ramifera, Macandrewia minima* sp. nov., *M. robusta, Leiodermatium lynceus* and *Exsuperantia levii* sp. nov.) were only found on one of the seamounts. These findings concur with a study on lithistids of the Norfolk Ridge (New Caledonia) where the authors reported 16 species (seven new to science, including a new genus) with the half of the species (eight) restricted to one seamount (Schlacher-Hoenlinger, Pisera & Hooper, 2005). On the other hand, five species, *M*. cf. *azorica, M. schusterae* sp. nov*., S. elongatus* sp. nov., *L. tuba sp. nov*. and *E. archipelagus*, have a wider distribution (found in three to five seamounts), and the latter three are shared between the two seamount groups. The differences in diversity and distribution found in our study may be a result of uneven sampling effort between the different seamounts (between 2 and 35 stations) and the two seamount groups (92 stations in *Seamount 1* vs 131 stations in *Seamount 2*).

When examined at a larger scale, seamounts share most species with the Azores and Canary archipelagos, with seven (*D. ramifera*, *D. verrucosa*, *M. azorica*, *M. robusta*, *E. archipelagus*, *L. lynceus*, *P*. (*P*.) *grimaldii*) and six species (*D. verrucosa*, *M. azorica*, *E. archipelagus*, *L. lynceus*, *L. tuba* sp. nov. and *S. elongatus* sp. nov.) shared, respectively. Given the relative proximity between localities and the oceanographic setting, it would be expected that the Azores would share more species with the Great Meteor group, instead of the Canaries, Madeira, Selvagens and the continental shelf of the Lusitanian group (Fig. 1). However, this is not observed in our study as only two species (*D. ramifera* and *M. robusta*) are exclusively shared between the Azores and the Meteor Seamount group. One species (*L. lynceus*) is common to Azores, Madeira, Canaries and the two groups of seamounts, and two species (*M. azorica* and *D. verrucosa*) are shared between the Meteor group and the oceanic islands. All the species found in the Lusitanian group are shared with the archipelagos and/or the Meteor Seamount, with only one exception, *N. inaequalis* sp. nov. that is exclusively known from the Gorringe Seamount. However, none of the species reported from the Portuguese (*Corallistes elegantior* Schmidt, 1870) and Moroccan continental shelves (*Theonella annulata* Lendenfeld, 1907) were found to occur in the Lusitanian seamounts group. It should be noted that the description of *C. elegantior* is vague and does not provide a detailed characterization of all spicules. Moreover, this species was never observed since its description by Schmidt (1870) in Portugal or in the surrounded areas, thus it should be considered a *taxon inquirendum*. *Neophrissospongia nolitangere* Pisera & Vacelet, 2011 a species reported from all oceanic islands (Carvalho, Pomponi & Xavier, 2015; Cruz, 2002; Topsent, 1904) and the Mediterranean sea (Manconi, 2011; Pisera & Vacelet, 2011) and *Corallistes masoni* Bowerbank, 1869 reported from Madeira (Bowerbank, 1869; Carvalho, Pomponi & Xavier, 2015) and Canary Islands, were also not found in this study. If we compare the diversity between NEA and the Mediterranean Sea, only five species, viz. *N. nolitangere*, *Neoschrammeniella bowerbankii* (Johnson, 1863), *L. lynceus*, *L. pfeifferae* (Carter, 1876) and *Siphonidium ramosum* (Schmidt, 1870) out of 36, are shared between these two areas. Finally, whether some of the species here described for the first time are shared with the Northwest Atlantic and/or the Caribbean Sea also remains to be assessed, since the lithistid fauna of these areas is known to be far more diverse than currently reported but awaits formal description (A. Pisera, 2018, personal communication; Schuster et al., 2019). Therefore, and given the still limited and uneven sampling of the various areas, we refrain from considering the species herein described endemic to these seamounts or seamount groups.

Future studies employing a more comprehensive sampling design and modern technologies would be required to test the extent to which an interplay between intrinsic (dispersal potential) and extrinsic (seamount age, isolation and area) factors underpin and shape the observed diversity and endemism patterns of the fauna of these seamounts.

### Spicules dimensions

Several morphological features are used in taxonomy and classification of Porifera and among them, the skeletal elements (spicules, fibres) and their arrangement are the most used. This is mainly due to historical reasons, since specimens would be sent for taxonomic assignment, sometime after collection and preservation, and usually having lost some of its live characteristics such as colour or consistency (Bergquist, 1970). Spicules sizes, which occur over a relatively large range are also important for species determination (Bergquist, 1970), altough some studies have shown that biophysical environmental conditions and life cycle can lead to some intraspecific varibaility (Bavestrello, Bonito & Sarà, 1993; Cárdenas & Rapp, 2013; Mercurio et al., 2000). In the case of lithistids sponges, the identification is mainly based on the shape and development of desmas and other accompanying spicules (Bergquist, 1970; Lévi, 1991).

Whether spicule size is as relevant for lithistids as in other taxonomic groups remains to be assessed. However, in the material examined in our study, we have found some differences in the size of the spicules for some species in comparison with the type material. Examples include *D. ramifera, D. verrucosa, M*. cf. *azorica, M. robusta*, *E. archipelagus* and *P. (P). grimaldii*. Specimens of *D. ramifera* and *D. verrucosa* despite being slightly larger than the holotypes and having been sampled at similar depths, present smaller cladomes of the discotriaenes, as well as their acanthomicroxeas and acanthorhabds (Table 2). In the case of *M*. cf. *azorica* and *M. robusta* the same pattern repeats, with exception of the microxeas on both specimens analysed here which are larger than those in the respective holotypes (Table 3). Finally, in *E. archipelagus* all the spicules are smaller than those in the holotype, even though the specimen itself has nearly the same size as the type material (Table 4). *P. (P.) grimaldii* is the only one that has slightly larger spicules compared with the type material (Table 7). These variations were also found in other deep water tetractinellids and were assumed to be related to the depth and/or silica concentration, where deeper specimens have larger spicules due to the availability of silica in the water (Cárdenas & Rapp, 2013). However, one cannot find a correlation with the depth since: (1) *D. ramifera* and *D. verrucosa* were sampled at similar depths as the holotypes, (2) the depth at which the type material of *M. azorica* was sampled is unknow preventing us to make any assumption, (3) *M. robusta* was found at shallower depths in the Hyères seamount and yet its spicules were in general smaller, (4) *P*. (*P*.) *grimaldii* was found within the same depth range as the holotype and has larger spicules, thus the depth seems to not be related with the size of the spicules. The amount of silica in the water does not seem to be related either since these two groups of seamounts have many lithistids, and they possibly require large amounts of silica to build their skeleton. Another explanation is that lithistids are very efficient at removing the silica from the water thus, not requiring large amounts of this element (Alvarez et al., 2017; Maldonado et al., 2015). Since there is no data regarding the biogeochemical parameters of the water column upon the time of collection of the material, it remains unclear if the cause of this variation are abiotic factors or intraspecific variation due to distinctive geographical area, as it was also observed in other astrophorins (Van Soest, Beglinger & de Voogd, 2010) including lithistids (Pisera & Vacelet, 2011).

## Conclusions and Identification Key

The discovery of ten new lithistid species in the NE Atlantic seamounts and the additional record of another seven species, emphasises how diverse these ecosystems are and how our knowledge on the diversity of this group of sponges is still limited. Whether the patterns of distribution here reported are due to sampling bias, or true cases of endemism, requires further investigation.

The factors behind the variability on the spicules sizes, found in some species compared to those of the holotypes, remain unclear and more studies are needed in order to shed light on the factors behind this variability. This is particularly important on the field of sponge taxonomy since spicules are a key element for their identification. Future expeditions to these seamounts, with the use of ROVs, will allow us to have a better picture of this diversity and confirm if there are sponge grounds dominated by lithistids in the area.

An identification key of all lithistid species reported to date for the NE Atlantic and Mediterranean Sea is presented below (Table 9).

**Table 9:** Identification key for lithistid demosponges from the Northeast Atlantic Ocean and Mediterranean Sea.

1.	Desmas are dicranoclones	2. Corallistidae
	Desmas are tetraclones, ectosomal spicules are phyllotriaenes or discotriaenes	8. Theonellidae
	Desmas have a triaenose crepsis, rarely monaxial crepis	11. Macandrewiidae
	Demas are triders	13. Phymaraphiniidae
	Desmas are rhizoclones, no ectosomal spicules, microscleres absent, raphides may be present	15. Azoricidae
	Desmas are rhizoclones, ectosomal spicules, if present, are rhabds or oxeas, sigmaspires may be present	17. Scleritodermidae
	Desmas are rhizoclones, no ectosomal spicules, exotylostyles present	20. Siphonidiidae
	Demas are monaxial or probably monaxial but not rhizoclones, styles are present	23. Desmanthidae
2.	Dichotriaenes with spines and tubercles on the top of the cladome, microscleres are streptasters/amphiasters	3. *Neophrissospongia*
	Dichotriaenes are smooth	4.
3.	Cup-shaped, dichotriaenes with very massive, thick and irregular cladomes; dicranoclones extremely tuberculated with a central core; spinose microstyles	*N. endoumensis*
	Encrusting thick plate with rounded margins, two types of dichotriaenes: with few tubercles or smooth; triaenes with few tubercles present, but rare; styles/sub-tylostyles	*N. nana*
	Ear- or cup-shaped when young to large to flabellate masses when old; dicranoclones very tuberculated; spinose microtylostyles	*N. nolitangere*
	Clavate in habitus with a narrow and central spongocoel; dicranoclones have sparsely distributed round tubercles; spinose microstylostyles	*N. radjae*
4.	One type of microscleres, spirasters with pointed arms	5. *Corallistes*
	Desmas have a root/vine-like appearance, microscleres are two types of microacanthoxeas, spirasters and streptasters	6. *Isabella*
	Two types of microsclers (metasters and spirasters), oxeas usually present	7. *Neoschrammeniella*
5.	No proper description has been given to this species in the original description and there are no more records of this species. The type material should be re-examined	*C. elegantior*
	Sinuously fan-shaped with rounded and thin walls; microscleres are spirasters with long and thin arms	*C. masoni*
6.	Irregular rounded sponge of dark purple-brown colour; ectosomal spicules are irregular dichotriaenes, short- and long-shafted triaenes; two types of long oxeas (type I: long and thick with blunt tips; type II long, thin, curved with acerate tips)	*I. harborbranchi*
7.	Cup-shaped to contorted lamellate masses with thick walls; smooth surface; several thin oxeas in the inner surface; dicranoclones have irregular and high tubercles, that can be subdivided into several smaller tubercles	*N. bowerbankii*
	Cup- to flattened cup-shaped with a concave center and rounded edges; smooth surfaces; dichotriaenes are very variable in shape and size; long-shafted triaenes can be present; oxeas are large and thin; dicranoclones of vine-like appearance, with some tubercles that are smooth or rugose	*N. inaequalis*
	Large cup-rectangular in shape with smooth surfaces; dicranoclones are irregular, compact, usually smooth, with few tubercles that are usually smooth; no oxeas; some microscleres are irregular, resembling irregular rhabds with spiny tips	*N. piserai*
	Cup-rounded in shape with a small pedicel; surfaces are crumble and hispid; oxeas are long with sharp tips; dicranoclones are compact, densely covered by numerous and ornamented tubercles	*N. pomponiae*
8.	Ectosomal spicules are discotriaenes, desmas are tetraclones, oxeas usually present, microscleres are acanthoxeas and acanthorhabds	9. *Discodermia*
	Ectosomal spicules are phyllotriaenes to discotriaenes, microscleres are acanthorhabds	10. *Theonella*
9.	Tree-like shaped, with a long stem smooth surface with some rugosities/protuberances; discotriaenes of “square” to “circular” shape or with “idented” cladomes; oxeas not present; tetraclones very tuberculated near the surface and smoother in the inner part of the sponge.	*D. arbor*
	Massive, irregular in shape, with large protuberances of round shape; rugose surface; discotriaenes very variable in the shape of the cladomes varying from oval to indented, and size of rhabdomes; strongyles with one tip rounded and the other sharp	*D. kellyae*
	Small irregular mushroom shaped, with a concave upper side, a short stem and smooth surface; discotriaenes with a round to oval cladome; tetraclones with smooth rays and strongly branched and tuberculated zygomes; oxeas	*D. polydiscus*
	Small, polymorphic, varying from spherical to irregular masses with protuberances, attached by a short pedicel; smooth surface; discotriaenes have very variable cladomes, from circular and concave to oval with irregular margins; tetraclones are smooth and irregular; oxeas not present	*D. polymorpha*
	Small, elongated and branched with a smooth surface; discotriaenes have a round/oval to irregular and indented cladome; oxeas; tetraclones have smooth rays and tuberculated zygoses, that are usually smooth	*D. ramifera*
	Cup-shaped to spherical polymorphic, with several round protuberances; discotriaenes are round/oval, smooth, often indented; oxeas; tetraclones are large, robust densely covered by tubercles	*D. verrucosa*
10.	Tetraclones are tuberculated but sometimes smooth in the center; phyllotriaenes have a simple or bifurcated cladome with rounded edges, and a short rhabdome*	*T. annulata*
11.	Dentate ectosomal phyllotriaenes/discotriaenes, smooth oxeas, microscleres are smooth microxeas.	12. *Macandrewia*
12.	Cyathiform to flabellate, with undulating rounded margins and a short stem; outer surface is smooth with small pores and inner surface is smooth but the oscules have slightly raised margins; desmas are smooth, either resembling tetraclones or rhizoclones, very branched at the end	*M. azorica*
	Small round-globular shaped, with a very short and slender pedicel and smooth surface; phyllotriaenes have incised and tuberculated cladomes; desmas with triaenose crepsis, usually smooth but some rugosities can be present	*M. minima*
	Sponge with a vast base where it stands two or more truncks of cylindrical shape, with the top divided into short and obtuse branches	*M. ramosa*
	Ficiform to globular in shape, with a thick and short pedicel; top of the sponge can be curved or slightly depressed; monocrepid desmas are smooth, with short and thick tubercles	*M. robusta*
	Foliate to vase shape, with thick and contorted lamellas and a small pedicel; phyllotriaenes with incised cladomes on the edges; desmas with a triaenose crepsis, smooth, irregular, with the several short and blunt branches at the end	*M. schusterae*
13.	Clavate or globular knob-like shaped, ectosomal spicules are phyllo- to discotriaenes; subtylostyles to tylotes; microscleres are acanthorhabds, acanthomicroxeas and steptasters/amphiasters	14. *Exsuperantia*
14.	Columnar to ficiform, sometimes with lateral protuberances/branches; smooth surface ectosomal spicules are phyllotriaenes.	*E. archipelagus*
	Clusters of globular to ficiform knob-like short fingers with an apical osculum; surface is rugose; ectosomal spicules are phyllo- to discotriaenes	*E. levii*
15.	Foliate or vase shaped in habitus, long oxeas, no microscleres	16. *Leiodermatium*
16.	Narrow ear-shaped or cylindrical sponge with deeply incised rounded margin; outer surface with oscules located on top of small elevations; inner surface with densely distributed pores	*L. lynceus*
	Flattish, cabbage-like, infoliated, with branched sinuous laminae; outer surface has pores and inner surface has scattered ostia slightly raised on papillary eminences; rhizoclones with branched arms	*L. pfeifferae*
	Massive lamellate vase to contorted thin walls, sometimes forming a cone; surfaces are smooth and similar at naked eye; outer surface has slightly depressed openings while the inner surface has several small openings and numerous oxeas	*L. tuba*
17.	Ectosomal spicules are acanthorhabds/strongyles, no microscleres	18. *Aciculites*
	Vase or foliate in shape, ectosomal spicules absent, microscleres present	19. *Microscleroderma*
18.	Massive cerebellum-like, sub-oval with a wide base; surface is smooth with subdermal canals covered by a dermal membrane; inhalant areas are irregularly distributed in depressed concavities, while exhalant areas are elevated; anisostrongyle to tylostrongyles, usually with a spinose/rugose head	*A. mediterranea*
19.	Irregular mass of contorted, irregularly undulating lamellae; two types of oxeas: thick, straight with acerate tips or thin, hair-like oxeas rarely straight; sigmaspires are C- or S-shaped with short spines	*M. lamina*
20.	With long fistules, exotylostyles with ornamented heads	21. *Siphonidium*
	No fisutles, deep and narrow atrial cavity, ear- or vase-shaped	22. *Gastrophanella*
21.	Small, irregularly massive to cylindrical in shape with numerous small fistules; desmas are tuberculated	*S. ramosum*
	Polymorphic, cilindrical to arborescent, sometimes bulb-shaped with numerous fistules; very spiny rhizoclones with slim arms ornamented with microspines in the edges; styles present (rare)	*S. elongatus*
22.	Irregular pear-shaped with lateral depressed shallow concavities and an osculum on the top; monaxons with a slightly protruding head	*G. phoeniciensis*
23.	Massive in habitus, desmas branching in various planes	24. *Petromica*
	Encrusting in habitus, no microscleres	25.
24.	Massive with conical form; irregular surface with conules and dispersed pores; desmas are poorly articulated with the tips divided into massive low spines; anisoxeas to styles (or strongyloxeas) and anisorhabds	*P. (Petromica) grimaldii*
25.	Desmas of the outer layer are trider-like	26. *Desmanthus*
	Desmas hook-like, with pointed spines	27. *Sulcastrella*
26.	Encrsuting with a hispid outer surface; desmas are of two types: trider-like, tri- to tetrapodial, branched in the upper parts and tuberculated in the surface; styles are long	*D. incrustans*
27.	Small circular encrusting sponge with a hispid surface; monocrepid desmas are irregular, strongly tuberculated in some places and form a solid basal crust; styles are slightly curved	*S. tenens*

**Notes:**

* The original description of *T. annulata* was based on fragmented material and detailed information regarding the species’ habitus and spicules are missing.

Sources: Carter (1873), Carvalho & Pisera (2019), Lendenfeld (1907), Manconi, Serusi & Pisera (2006), Manconi & Serusi (2008), Perez et al. (2004), Pisera & Lévi (2002a), Pisera & Lévi (2002d), Pisera & Lévi (2002f), Pisera & Lévi (2002g), Pisera & Lévi (2002h), Pisera & Lévi (2002i), Pisera & Vacelet (2011), Topsent (1904) and Vacelet (1969).

## Supplemental Information

10.7717/peerj.8703/supp-1 Supplemental Information 1 List of specimens analysed in this study with detailed information of the stations in which they were sampled. All specimens are deposited at MNHN Paris.

## Abbreviations

AZO: Azores
CAN: Canaries
MAD: Madeira
MED: Mediterranean Sea
NEA: Northeast Atlantic Ocean
NWA: Northwest Atlantic Ocean
PT: Portugal
SEL: Selvagens
CP: beam trawl
DE: epibenthic dredge
DW: Warén dredge
DOP: Department of Oceanography and Fisheries of the University of Azores
HBOI: Harbour Branch Oceanographic Institute, Fort Pierce, FL, U.S.A
MNHN: Muséum National d’Histoire Naturelle of Paris, France
RMNH: National Museum of Natural History Naturalis, Leiden, The Netherlands

## Additional Information and Declarations

### Data Availability

The following information was supplied regarding data availability: Data is available at PANGAEA: Carvalho, Francisca C; Cárdenas, Paco; Rios, Pilar; Rapp, Hans Tore; Xavier, Joana R (2018): Lithistids from the Seamounts of the Northeast Atlantic Ocean. PANGAEA, DOI 10.1594/PANGAEA.896492. Specimens examined in this study are stored in the ’zoothèque’ of the Muséum National d’Histoire Naturelle of Paris, France (MNHN). Specimen numbers and deposition information are available as a Supplemental File.

### New Species Registration

The following information was supplied regarding the registration of a newly described species: Publication LSID: urn:lsid:zoobank.org:pub:A0DA0236-4579-47A4-8BE4-E68803C2EC8F. *Neoschrammeniella inaequalis* sp. nov.: Urn:lsid:zoobank.org:act:8A516D9B-5351-47AF-8EC2-7EBC44166D35. *Neoschrammeniella piserai* sp. nov.: Urn:lsid:zoobank.org:act:77F1F52E-28C9-43C0-A501-1ADAD03241A5. *Neoschrammeniella pomponiae* sp. nov.: urn:lsid:zoobank.org:act:2AA76193-B27E-491E-8E50-FE591786FA26. *Discodermia arbor* sp. nov.: Urn:lsid:zoobank.org:act:7A732A92-8D8B-4D73-97B1-CD53E9494121. *Discodermia kellyae* sp. nov.: urn:lsid:zoobank.org:act:E7A06142-4AF7-404E-B369-B30240ADE5F4. *Macandrewia schusterae* sp. nov.: urn:lsid:zoobank.org:act:2BA2C1EF-8FAB-4C91-89CB-DCB59DDA61EB. *Macandrewia minima* sp. nov.: urn:lsid:zoobank.org:act:E405AE49-5636-4778-9B07-ED39E9EBB7BE. *Exsuperantia levii* sp. nov.: urn:lsid:zoobank.org:act:24B5934A-4767-4429-B172-A649C4CE0D83. *Leiodermatium tuba* sp. nov.: urn:lsid:zoobank.org:act:041DAB82-B538-4EB9-A43A-1E3E79B67CF8. *Siphonidium elongatus* sp. nov.: urn:lsid:zoobank.org:act:26B193F9-2588-4479-ACB2-27AD1945DEE4.
