# Supplementary material for: Rock sponges (lithistid Demospongiae) of the Northeast Atlantic seamounts, with description of ten new species
Source: PeerJ. 2020 Apr 7;8:e8703. doi: 10.7717/peerj.8703 (PMC7147441; doi:10.7717/peerj.8703)
Supplement: Supplemental Information 1 [file peerj-08-8703-s001.docx]

| **Supplementary Material 1:**  List of specimens analysed in this study with detailed information of the stations in which they were sampled. All specimens are deposited in the MNHN Paris. | | | | | | | | | | | | |
| --- | --- | --- | --- | --- | --- | --- | --- | --- | --- | --- | --- | --- |
| **Species** | **Voucher number** | **Nº of specimens** | **Locality** | **Station** | **Latitude** | **Longitude** | | **Depth (m)** | **Collection date** | **Vessel** | **Collection mode** | **Campaign** |
| *Neoschrammeniella inaequalis* **sp. nov.** | MNHN-IP-2018-86 | 1 | Gorringe | DW21 | 36º34.9 | | 11º28.4 | 460–480 | 24/09/1988 | Noroît | DW | Seamount 1 |
|  | MNHN-IP-2018-84 | 1 | Gorringe | CP28 | 36º38.0 | | 11º29.8 | 605–675 | 26/09/1988 | Noroît | CP | Seamount 1 |
|  | MNHN-IP-2018-85 | 1 | Gorringe | CP28 | 36º38.0 | | 11º29.8 | 605–675 | 26/09/1988 | Noroît | CP | Seamount 1 |
| *Neoschrammeniella piserai* **sp. nov.** | MNHN-IP-2008-234 | 1 | Plato | DW241 | 33°12’N | | 28°59’W | 695 | 31/01/1993 | Suroît | DW | Seamount 2 |
| *Neoschrammeniella* *pomponiae* **sp. nov.** | MNHN-IP-2008-233 | 1 | Hyères | DW182 | 31°23’N | | 28°54’W | 480 | 16/01/1993 | Suroît | DW | Seamount 2 |
| *Neoschrammeniella* sp. (fragment) | MNHN-IP-2018-95 | 1 | Gorringe | DW27 | 36º37.0 | | 11º29.4 | 580–610 | 26/09/1988 | Noroît | DW | Seamount 1 |
| *Discodermia ramifera* Topsent, 1892 | MNHN-IP-2008-204 | 1 | Great Meteor | CP138 | 30°02’N | | 28°29’W | 300 | 09/01/1993 | Suroît | CP | Seamount 2 |
|  | MNHN-IP-2008-207 | 1 | Great Meteor | DE140 | 30°01’N | | 28°28’W | 308 | 10/01/1993 | Suroît | DE | Seamount 2 |
|  | MNHN-IP-2008-213 | 1 | Great Meteor | CP156 | 29°56’N | | 28°24’W | 320 | 11/01/1993 | Suroît | CP | Seamount 2 |
|  | MNHN-IP-2008-214 | 1 | Great Meteor | CP144 | 30°10’N | | 28°29’W | 335 | 10/01/1993 | Suroît | CP | Seamount 2 |
| *Discodermia* cf*. ramifera* Topsent, 1892 | MNHN-IP-2008-210 | 1 | Atlantis | DW258 | 34°00'N | | 30°12'W | 420 | 02/02/1993 | Suroît | DW | Seamount 2 |
|  | MNHN-IP-2008-205 | 2 | Atlantis | CP257 | 34°04'N | | 30°15'W | 338 | 02/02/1993 | Suroît | CP | Seamount 2 |
|  | MNHN-IP-2008-206 | 1 | Plato | DW246 | 33°14'N | | 29°36'W | 520 | 01/02/1993 | Suroît | DW | Seamount 2 |
| *Discodermia arbor* **sp. nov.** | MNHN-IP-2008-211 | 1 | Great Meteor | DW159 | 29°44'N | | 28°20'W | 330 | 11/01/1993 | Suroît | DW | Seamount 2 |
| *Discodermia kellyae* **sp. nov.** | MNHN-IP-2008-208 | 1 | Plato | DW247 | 33°14'N | | 29°35'W | 580 | 01/02/1993 | Suroît | DW | Seamount 2 |
| *Discodermia* sp. (deciduous specimen) | MNHN-IP-2008-212 | 7 | Great Meteor | DW139 | 30°01'N | | 28°28'W | 304 | 10/01/1993 | Suroît | DW | Seamount 2 |
| *Macandrewia* cf*. azorica* Gray, 1859 | MNHN-IP-2008-217 | 1 | Atlantis | DW263 | 34°26’N | | 30°32’W | 610 | 03/02/1993 | Suroît | DW | Seamount 2 |
|  | MNHN-IP-2008-220 | 1 | Atlantis | DW258 | 34°00’N | | 30°12’W | 420 | 03/02/1993 | Suroît | DW | Seamount 2 |
|  | MNHN-IP-2008-225 | 5 | Tyro | DW277 | 34°00'N | | 28°21'W | 1000 | 06/02/1993 | Suroît | DW | Seamount 2 |
|  | MNHN-IP-2008-226 | 1 | no data | no data | no data | | no data | 500 | Jan-93 | Suroît | no data | Seamount 2 |
|  | MNHN-IP-2008-229 | 1 | Gran Canaria | DW129 | 28°08’N | | 15°52’W | 480 | 06/01/1993 | Suroît | DW | Seamount 2 |
|  | MNHN-IP-2008-249a | 19 | Hyères | DW202 | 31°16'N | | 28°43'W | 640 | 19/02/1993 | Suroît | DW | Seamount 2 |
| *Macandrewia schusterae* **sp. nov.** | MNHN-IP-2018-87 | 1 | Gorringe | CP28 | 36°28’N | | 11°29’W | 605–675 | 26/09/1988 | Noroît | CP | Seamount 1 |
|  | MNHN-IP-2018-90 | 6 | Gorringe | CP28 | 36°28’N | | 11°29’W | 606–675 | 26/09/1988 | Noroît | CP | Seamount 1 |
|  | MNHN-IP-2018-88 | 1 | Gorringe | CP28 | 36°28’N | | 11°29’W | 605–675 | 26/09/1988 | Noroît | CP | Seamount 1 |
|  | MNHN-IP-2018-91 | 1 | Gorringe | CP28 | 36°28’N | | 11°29’W | 605–675 | 26/09/1988 | Noroît | CP | Seamount 1 |
|  | MNHN-IP-2008-219 | 1 | Tyro | DW279 | 33°56'N | | 28°24'W | 805 | 06/02/1993 | Suroît | DW | Seamount 2 |
|  | MNHN-IP-2008-230 | 4 | Plato | DW246 | 33°14'N | | 29°36'W | 520 | 01/02/1993 | Suroît | DW | Seamount 2 |
| *Macandrewia minima* **sp. nov.** | MNHN-IP-2008-222 | 2 | Great Meteor | DW148 | 30°12’N | | 28°25’W | 615 | 11/01/1993 | Suroît | DW | Seamount 2 |
| *Macandrewia robusta* Topsent, 1904 | MNHN-IP-2008-216 | 25 | Hyères | DW184 | 31°24’N | | 28°52’W | 705 | 16/01/1993 | Suroît | DW | Seamount 2 |
|  | MNHN-IP-2008-224 | 2 | Hyères | DW184 | 31°24'N | | 28°52'W | 705 | 16/02/1993 | Suroît | DW | Seamount 2 |
| *Macandrewia* sp. | MNHN-IP-2008-228 | 1 | Antialtair | DW281 | 43°35'N | | 22°26'W | 900 | 09/02/1993 | Suroît | DW | Seamount 2 |
|  | MNHN-IP-2018-94 | 1 | Ampére | CP99 | 35°04’'N | | 12º55W | 225–280 | 12/10/1988 | Noroît | CP | Seamount 1 |
| *Leiodermatium lynceus* Schmidt, 1870 | MNHN-IP-2018-93 | 1 | Gorringe | CP20 | 36º33.7 | | 11º30W | 305–320 | 24/09/1988 | Noroît | CP | Seamount 1 |
|  | MNHN-IP-2008-239 | 1 | Hyères | DW182 | 31°23'N | | 28°54'W | 480 | 16/01/1993 | Suroît | DW | Seamount 2 |
| *Leiodermatium tuba* **sp. nov.** | MNHN-IP-2018-74 | 1 | Gorringe | DW25 | 36º49.7 | | 11º03.3 | 970–1035 | 24/09/1988 | Noroît | CP | Seamount 1 |
|  | MNHN-IP-2018-75 | 1 | Gorringe | CP11 | 36º26.4 | | 11º40.2 | 805–830 | 23/09/1988 | Noroît | CP | Seamount 1 |
|  | MNHN-IP-2018-76 | 1 | Gorringe | CP11 | 36º26.4 | | 11º40.2 | 805–830 | 23/09/1988 | Noroît | CP | Seamount 1 |
|  | MNHN-IP-2018-73 | 1 | Gorringe | CP11 | 36º26.4 | | 11º40.2 | 805–830 | 23/09/1988 | Noroît | CP | Seamount 1 |
|  | MNHN-IP-2018-72 | 1 | Gorringe | CP11 | 36º26.4 | | 11º40.2 | 805–830 | 23/09/1988 | Noroît | CP | Seamount 1 |
|  | MNHN-IP-2008-235 | 1 | Plato | DW242 | 33º12'N | | 28º57'W | 710 | 31/01/1993 | Suroît | DW | Seamount 2 |
|  | MNHN-IP-2008-237 | 1 | Atlantis | DW265 | 34°29'N | | 30°36'W | 545 | 03/02/1988 | Suroît | DW | Seamount 2 |
|  | MNHN-IP-2008-249b | 1 | Hyères | DW202 | 31°16'N | | 28°43'W | 640 | 19/02/1993 | Suroît | DW | Seamount 2 |
|  | MNHN-IP-2008-255 | 1 | Gran Canaria | DW130 | 28°09'N | | 15°53'W | 660 | 06/01/1993 | Suroît | DW | Seamount 2 |
|  | MNHN-IP-2008-253 | 1 | Great Meteor | DW159 | 29°44'N | | 28°20'W | 330 | 11/01/1993 | Suroît | DW | Seamount 2 |
| *Siphonidium elongatus* **sp. nov.** | MNHN-IP-2008-232 | 1 | Gran Canaria | DW128 | 28°08'N | | 15°52'W | 470 | 03/01/1993 | Suroît | DW | Seamount 2 |
|  | MNHN-IP-2008-236 | 1 | Atlantis | DW265 | 34°29'N | | 30°36'W | 545 | 03/02/1993 | Suroît | DW | Seamount 2 |
|  | MNHN-IP-2008-245 | 1 | Hyères | DW182 | 31°23'N | | 28°54'W | 480 | 16/01/1993 | Suroît | DW | Seamount 2 |
|  | MNHN-IP-2008-256 | 1 | no data | no data | no data | | no data | no data | no data | Suroît | DW | Seamount 2 |
|  | MNHN-IP-2018-80 | 1 | no data | no data | no data | | no data | no data | no data | Noroît | no data | Seamount 1 |
|  | MNHN-IP-2018-79 | 1 | Gorringe | CP28 | 36º38.0 | | 11º29.8 | 605–675 | 26/09/1988 | Noroît | CP | Seamount 1 |
|  | MNHN-IP-2018-81 | 1 | Gorringe | CP28 | 36º38.0 | | 11º29.8 | 605–675 | 26/09/1988 | Noroît | CP | Seamount 1 |
|  | MNHN-IP-2018-77 | 1 | Gorringe | DW21 | 36º34.9 | | 11º28.4 | 460–480 | 24/09/1988 | Noroît | DW | Seamount 1 |
|  | MNHN-IP-2018-78 | 1 | Gorringe | DW21 | 36º34.9 | | 11º28.4 | 460–480 | 24/09/1988 | Noroît | DW | Seamount 1 |
|  | MNHN-IP-2018-82 | 13 | Lion | DW63 | 35º15.4 | | 15º34.6 | 630 | 08/10/1988 | Noroît | DW | Seamount 1 |
|  | MNHN-IP-2018-83 | >50 | Gorringe | CP28 | 36º38.0 | | 11º29.8 | 605–675 | 26/09/1988 | Noroît | CP | Seamount 1 |
| *Exsuperantia archipelagus* Carvalho and Pisera, 2019 | MNHN-IP-2008-191 | 4 | Tyro | DW277 | 34°00'N | | 28°21'W | 1000 | 06/02/1993 | Suroît | DW | Seamount 2 |
|  | MNHN-IP-2008-192 | 6 | Atlantis | DW265 | 34°29'N | | 30°36'W | 545 | 03/02/1993 | Suroît | DW | Seamount 2 |
|  | MNHN-IP-2008-193 | 1 | Hyères | DW203 | 31°10'N | | 28°44'W | 845 | 19/01/1993 | Suroît | DW | Seamount 2 |
|  | MNHN-IP-2008-195 | 4 | Atlantis | CP257 | 34°04'N | | 30°15'W | 338 | 02/02/1993 | Suroît | DW | Seamount 2 |
|  | MNHN-IP-2008-196 | 6 | Hyères | DW188 | 31°30'N | | 29°00'W | 310 | 17/01/1993 | Suroît | DW | Seamount 2 |
|  | MNHN-IP-2008-197 | 4 | Atlantis | DW274 | 34°05'N | | 30°14'W | 280 | 05/02/1993 | Suroît | DW | Seamount 2 |
|  | MNHN-IP-2008-198 | 3 | Gran Canaria | DW130 | 28°09'N | | 15°53'W | 660 | 06/01/1993 | Suroît | DW | Seamount 2 |
|  | MNHN-IP-2008-199 | 1 | Atlantis | DW258 | 34°00'N | | 30°12'W | 420 | 02/02/1993 | Suroît | DW | Seamount 2 |
|  | MNHN-IP-2008-200 | 2 | Plato | DW242 | 33°12'N | | 28°57'W | 710 | 31/01/1993 | Suroît | DW | Seamount 2 |
|  | MNHN-IP-2008-202 | 2 | Atlantis | DW254 | 34°05'N | | 30°13'W | 280 | 02/02/1993 | Suroît | DW | Seamount 2 |
|  | MNHN-IP-2008-240 | 4 | Plato | DW246 | 33°14'N | | 29°36'W | 520 | 01/02/1993 | Suroît | DW | Seamount 2 |
|  | MNHN-IP-2008-243 | 1 | Atlantis | DW258 | 34°00'N | | 30°12'W | 420 | 02/02/1993 | Suroît | DW | Seamount 2 |
| *Exsuperantia levii* **sp. nov.** | MNHN-IP-2008-201 | 5 | Hyères | DW182 | 31°23'N | | 28°54'W | 480 | 16/01/1993 | Suroît | DW | Seamount 2 |
| *Petromica (P.) grimaldii* Topsent, 1898 | MNHN-IP-2018-92 | 1 | Gorringe | DW16 | 36º31.1 | | 11º32.5 | 255–265 | 24/09/1988 | Noroît | DW | Seamount 1 |
| Abbreviations used: CP- beam trawl; DE- epibenthic dredge; DW- epibenthic Warén dredge; MNHN- Muséum Nationale d'Histoire Naturelle, Paris, France. Several specimens collected in the stations CP28, CP99, DW21 and CP12 from *Seamount 1* and DW128, DW130, DW136, DW143, DW146, DW147, DW148, DW152, DW166, DW226, DW237, DW242, DW247, DW248, DW249, DW258 from *Seamount 2,* were deciduous and/or dead skeletons, and could not be confidently identified (not included here). | | | | | | | | | | | | |
|  | | | | | | | | | | | | |
|  | | | | | | | | | | | | |
